# Transcriptomic and metabolic studies on the role of inorganic and organic iodine compounds in lettuce plants

**DOI:** 10.1038/s41598-023-34873-7

**Published:** 2023-05-25

**Authors:** Sylwester Smoleń, Małgorzata Czernicka, Kinga Kęska-Izworska, Iwona Kowalska, Dariusz Grzebelus, Joanna Pitala, Mariya Halka, Łukasz Skoczylas, Małgorzata Tabaszewska, Marta Liszka-Skoczylas, Marlena Grzanka, Iwona Ledwożyw-Smoleń, Aneta Koronowicz, Joanna Krzemińska, Olga Sularz, Daniel Kiełbasa, Jakub Neupauer, Peter Kováčik

**Affiliations:** 1 Department of Plant Biology and Biotechnology, Faculty of Biotechnology and Horticulture, University of Agriculture in Krakow, Al. Mickiewicza 21, 31-120 Krakow, Poland; 2 Laboratory of Mass Spectrometry, Faculty of Biotechnology and Horticulture, University of Agriculture in Krakow, Al. Mickiewicza 21, 31-120 Krakow, Poland; 3 Department of Plant Product Technology and Nutrition Hygiene, Faculty of Food Technology, University of Agriculture in Krakow, Al. Mickiewicza 21, 31-120 Krakow, Poland; 4 Department of Engineering and Machinery for Food Industry, Faculty of Food Technology, University of Agriculture in Krakow, Al. Mickiewicza 21, 31-120 Krakow, Poland; 5 Department of Human Nutrition and Dietetics, Faculty of Food Technology, University of Agriculture in Krakow, Al. Mickiewicza 21, 31-120 Krakow, Poland; 6 Department of Agrochemistry and Plant Nutrition, Slovak University of Agriculture in Nitra, Tr. A. Hlinku 2, 949 01 Nitra, Slovakia

**Keywords:** Agricultural genetics, Plant physiology

## Abstract

Iodine (I) is considered a beneficial element or even micronutrient for plants. The aim of this study was to determine the molecular and physiological processes of uptake, transport, and metabolism of I applied to lettuce plants. KIO_3_, KIO_3_ + salicylic acid, 5-iodosalicylic acid and 3,5-diiodosalicylic acid were applied. RNA-sequencing was executed using 18 cDNA libraries constructed separately for leaves and roots from KIO_3_, SA and control plants. De novo transcriptome assembly generated 1937.76 million sequence reads resulting in 27,163 transcripts with N50 of 1638 bp. 329 differentially expressed genes (DEGs) in roots were detected after application of KIO_3_, out of which 252 genes were up-regulated, and 77 were down-regulated. In leaves, 9 genes revealed differential expression pattern. DEGs analysis indicated its involvement in such metabolic pathways and processes as: chloride transmembrane transport, phenylpropanoid metabolism, positive regulation of defense response and leaf abscission, and also ubiquinone and other terpenoid-quinone biosynthesis, protein processing in endoplasmic reticulum, circadian rhythm including flowering induction as well as a putative PDTHA (i.e. Plant Derived Thyroid Hormone Analogs) metabolic pathway. qRT-PCR of selected genes suggested their participation in the transport and metabolism of iodine compounds, biosynthesis of primary and secondary metabolites, PDTHA pathway and flowering induction.

## Introduction

The view on iodine importance to plants has changed over several decades. In the past I was considered a superfluous or harmful element to plants. Currently, it is assigned to the micronutrients^1^ or to beneficial elements^2,3^. The use of more and more advanced methods of instrumental analysis allows investigating metabolic pathways of iodine in plants^4^, which produces better results when combined with biotechnological and bioinformatics approach^1^. Microarray- and RT-qPCR-based studies on gene expression revealed the role of iodine in maize, wheat, tomato and *Arabidopsis thaliana*^1^. These authors demonstrated the beneficial effect of iodine (applied in micromolar doses of KI, NaI or KIO_3_) on *A. thaliana*. Iodine specifically regulated the expression of several genes e.g. involved in response of plants to stress factors. Proteomic data shown by Kiferle et al.^1^ allowed to determine that protein-bound iodine was present in chloroplasts. These authors indicated that in the roots, however, the iodinated proteins are related to the action of various peroxidases—these results suggest the functional involvement of iodine in plant nutrition^1^. To date, there are no reports on the transcriptomic analysis of lettuce plants treated with exogenous iodine. Smoleń et al.^4^ found that the vanadium-dependent haloperoxidase (vHPO) enzyme functionally related to the *per64-like* gene was likely involved in the process of iodine uptake by lettuce, and expression of the gene in the roots was higher than in the leaves.

PDTHAs [i.e. Plant-derived Thyroid Hormone Analogs] e.g. thyroxine (T4) and triiodothyronine (T3) are one of the least known plant metabolites described only in a few works so far. Fowden^5^ provided the basic information on PDTHA synthesis in plants after application of radioactive iodine. There are hypotheses on the possible regulatory role (or substrate function) of certain isoflavones in the metabolism of the PDTHA group in plants^6^. Garipova et al.^7^ revealed the presence of an analogue of 3,5,3′-triiodothyronine using immunoenzyme assay in the cells of several plants species and also suggested that exogenous T4 may be involved in gene regulation in cells of higher plants, similarly to that in animal cells. However, the available literature does not provide the evidence of the structure and function of T3 or T4 receptors in plant cells. Our previous research^4^ showed that 3,5-diiodosalicylic acid (3,5-diISA) and 5-iodosalicylic acid (5-ISA) are involved in the synthesis of PDTHAs with the process likely taking place in roots rather than leaves of lettuce plants. However, that study did not identify genes or enzymes involved in the process.

Salicylic acid (SA) is a plant growth regulator^8^ that is involved, among others, in the defense response during pathogenesis^9,10^ as well as in regulation of selected post-harvest physiological processes^11^. Exogenous SA may induce various effects including flowering stimulation and acceleration of vegetative growth^12^. SA has been shown to increase the phenolics content in plants as an effect of salinity stress^13^. What is more, interactions between SA and other plant phytohormones [such as auxins, jasmonic acid, abscisic acid or ethylene] in different abiotic and biotic stresses are also well recognized^8,14^.

Many studies using transcriptomic analysis have helped to determine the important role of SA in the molecular and biochemical response of plants to various stress factors^14,15^. However, it is not known whether and to what extent the exogenous and endogenous SA affect molecular processes related to the uptake and metabolism of iodine in plants. There is also no data on whether iodine-dependent regulation of plant metabolism and physiology is common or differ from SA-dependent processes. The study of these relationships at the transcriptome level, in conjunction with the analysis of the chemical composition, may allow to determine the physiological role of iodine in plants, including processes related to the synthesis and metabolism of PDTHAs.

Despite significant advances regarding basic iodine-dependent processes^1^, still many aspects related to the action and function of iodine in plants have not been discovered. The aim of the research was to determine—using transcriptomic and metabolomic approaches—the molecular processes that takes place in lettuce plants in response to exogenously applied iodine and SA. It was also aimed to evaluate the difference in plant response to inorganic and organic forms of iodine at a molecular level. The organic iodine compounds used in the current study, i.e. 5-iodosalicylic acid (5-ISA) and 3,5-diiodosalicylic acid (3,5-diISA) were selected basing on the previous works already confirming its efficient uptake and distribution within a plant^4,16^.

## Results

### RNA sequencing, de novo transcriptome assembly and functional annotation

Results of high throughput sequencing of 18 paired-end cDNA libraries from three independent biological samples of roots and leaves of plants treated with SA, KIO_3_ as well as control plants (Ctrl) are summarized in Table 1. After filtration of low-quality and adapter sequences, the number of high quality reads varied from 89,848,092 to 109,380,098 per sample. In NCBI SRA database the data were stored under BioProject PRJNA891733. De novo transcriptome was assembled and finally included 27,163 transcripts corresponding to 20,837 genes (Fig. S1A) with average length of 1916 bp (Fig. S1A,B). Functional annotation of clustered transcripts was blast-searched against NCBI, Pfam, eggnog, GO and KEGG databases (e-value < 1e − 5). 25,590 (94%) transcripts were successfully annotated in at least one database and 15,482 (57%) were annotated in all of databases. Furthermore 75% of annotated transcripts had a significant level of sequence similarity to *Arabidopsis thaliana*, 4% to *Oryza sativa* and 2% to *Lactuca sativa* (Fig. S1C,D).

**Table 1:** Summary of the RNA-seq data of *Lactuca sativa* L.

ORGAN	Treatment	Label in file name	Biological replicate	Total number of reads	Clean reads
ROOT	Ctrl	R-Ctrl_1	1	109,569,102	100,877,492 (92.07%)
		R-Ctrl_2	2	117,553,724	109,380,098 (93.05%)
		R-Ctrl_3	3	103,567,622	96,764,516 (93.43%)
	SA	R-SA_1	1	110,881,600	95,065,698 (85.74%)
		R-SA_2	2	108,465,218	95,923,638 (88.44%)
		R-SA_3	3	105,396,182	96,059,048 (91.14%)
	KIO_3_	R-KIO_3__1	1	110,054,860	98,489,530 (89.49%)
		R-KIO_3__2	2	109,044,694	96,437,318 (88.44%)
		R-KIO_3__3	3	103,696,860	92,758,078 (89.45%)
LEAF	Ctrl	L-Ctrl_1	1	112,009,180	97,559,258 (87.10%)
		L-Ctrl_2	2	106,402,450	92,771,352 (87.19%)
		L-Ctrl_3	3	102,658,510	92,119,194 (89.73%)
	SA	L-SA_1	1	108,363,458	96,990,090 (89.50%)
		L-SA_2	2	108,598,606	97,017,644 (89.34%)
		L-SA_3	3	101,874,256	91,293,230 (89.61%)
	KIO_3_	L-KIO_3__1	1	110,799,542	99,391,832 (89.70%)
		L-KIO_3__2	2	100,812,628	89,848,092 (89.12%)
		L-KIO_3__3	3	108,011,244	97,700,612 (90.45%)

### DEGs in response to KIO_3_ and SA treatment

**Figure 1:**
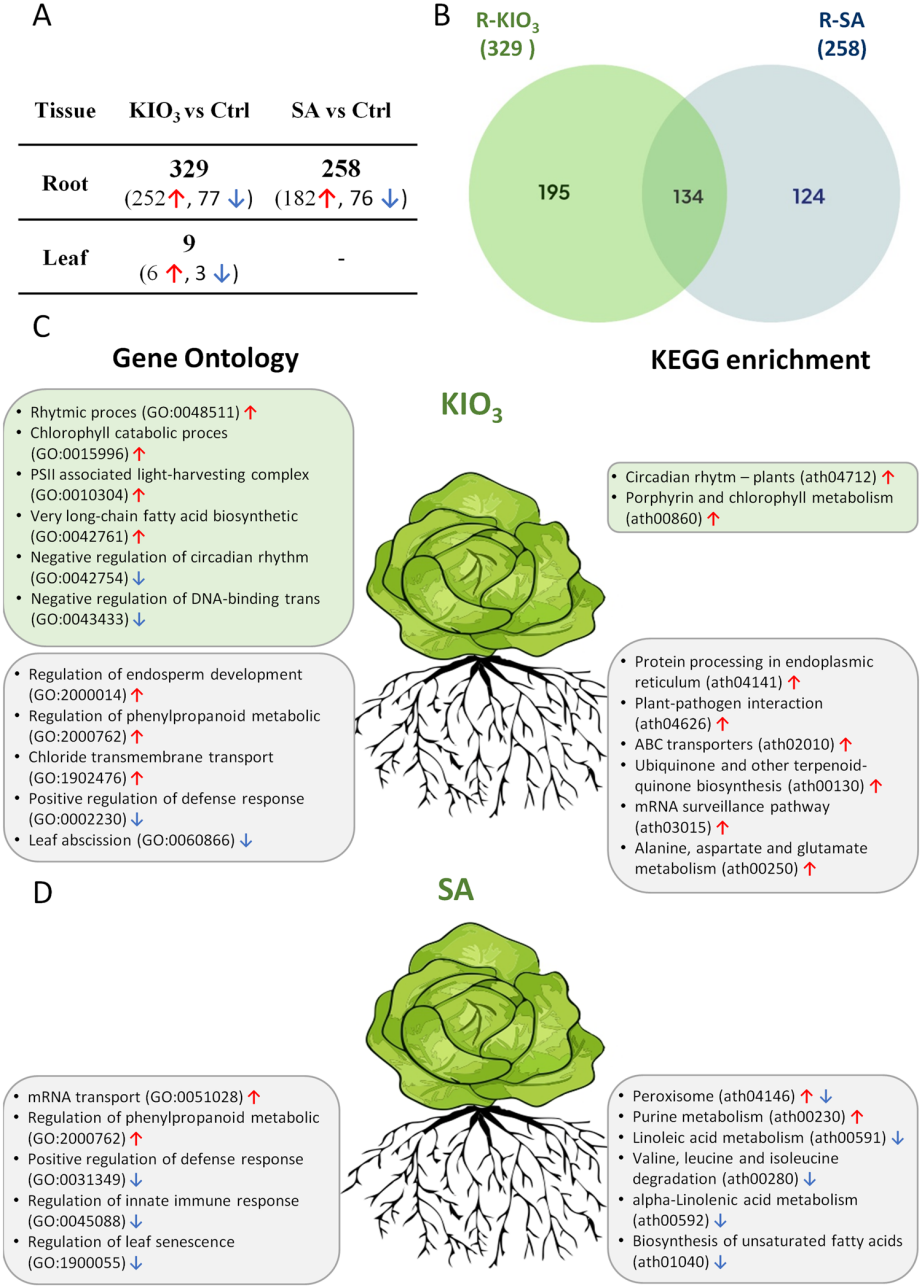
(**A**) Numbers of differentially expressed genes (DEGs) in lettuce roots and leaves supplemented with KIO_3_ and SA as compared to the control (Ctrl) plants (FDR < 0.05); (**B**) Venn diagram showing common and specific DEGs regulated in lettuce roots (R) supplemented with KIO_3_ and SA as compared to those grown in control conditions; (**C**) DEG enrichment based on gene ontology (GO) and Kyoto Encyclopedia of Genes and Genomes (KEGG) pathway systems identified in leaves and roots of KIO_3_- (**C**) and SA-treated (**D**) lettuce plants. Red and blue arrows indicate up- and down-regulation of genes in the pathway, respectively.

329 differentially expressed genes (DEGs) were identified in lettuce roots grown in the presence of KIO_3_. Of those, 252 and 77 were up- and down-regulated, respectively (Fig. 1A,B; Supplementary data S1). They were further analyzed in order to attribute them to pathways using KEGG and Gene Ontology databases (Fig. 1C,D; Table S1). Genes involved in processing endoplasmic reticulum (ath04141), plant-pathogen interactions (ath04626), as well as those coding for: ABC transporters (ath02010), ubiquinone and other terpenoid-quinone biosynthesis-related proteins (ath00130), proteins from the mRNA surveillance pathway (ath03015) and alanine, aspartate and glutamate metabolism (ath00250) were up-regulated in the roots. The up-regulated lettuce genes were assigned to such processes as: regulation of endosperm development (GO:2000014), phenylpropanoid metabolic process (GO:2000762) and chloride transmembrane transport (GO:1902476). Genes down-regulated in the roots of lettuce under KIO_3_ were involved in the processes of positive regulation of defense response (GO:0002230) and leaf abscission (GO:0060866). In the lettuce leaves, only nine genes were differentially expressed (Fig. 1A,B; Supplementary data S2).

Application of SA to the medium affected the expression of 258 genes roots in lettuce, while no DEGs were detected in the leaves (Fig. 1A,B; Supplementary data S3). In the roots, 182 and 76 genes were up- and down-regulated, respectively. The following GO terms were enriched: enhanced mRNA transport (GO:0051028), regulation of phenylpropanoid metabolic process (GO:2000762), inhibited regulation of defense response (GO:0031349), innate immune response (GO:0045088). The KEGG pathway analysis revealed that DEGs were involved in linoleic acid and alpha-linolenic acid metabolism (ath00591, ath00592), valine, leucine and isoleucine degradation (ath00280) and biosynthesis of unsaturated fatty acids (ath01040) (Table S1).

**Figure 2:**
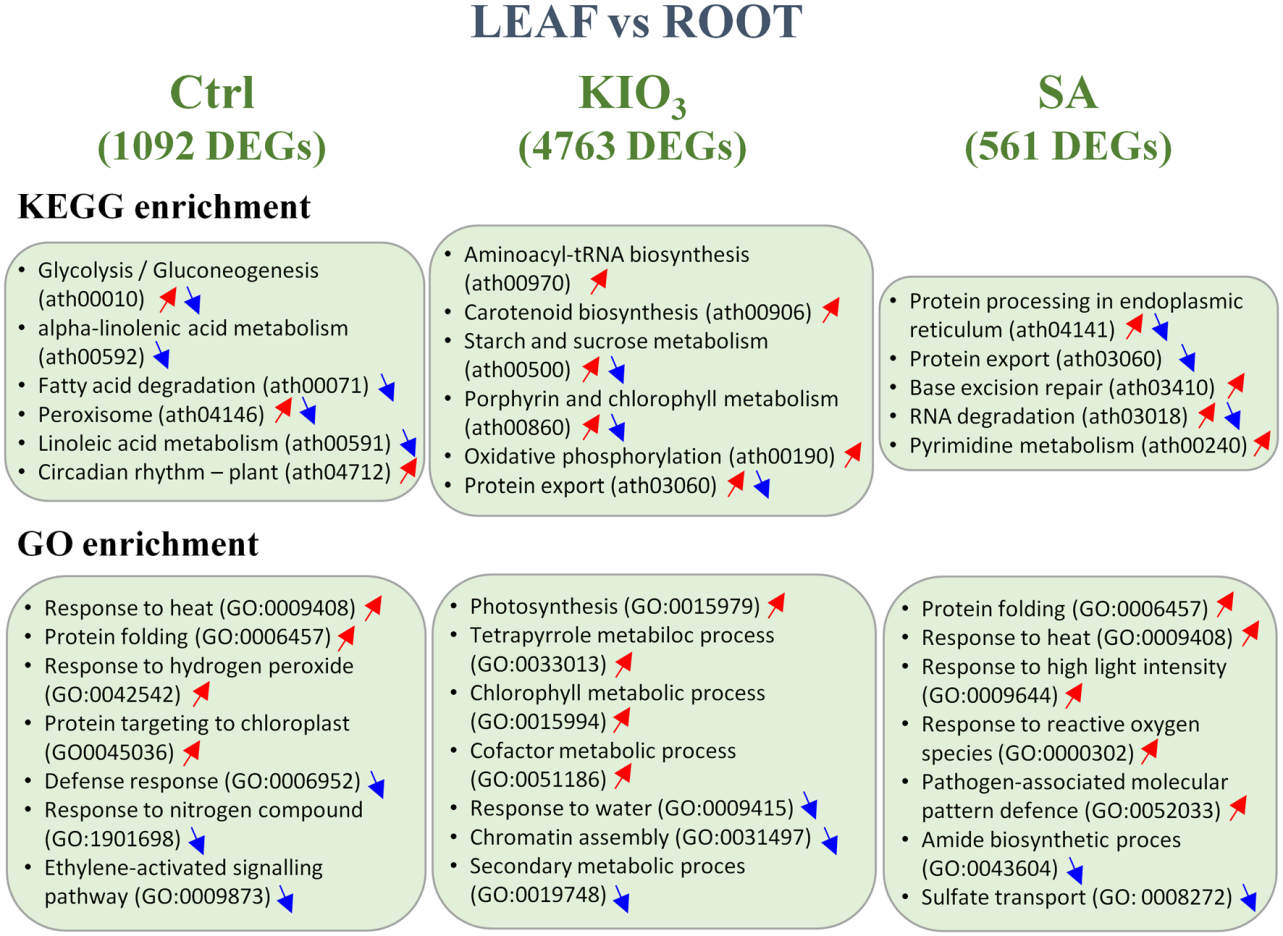
Differentially expressed genes (DEGs) between leaves and roots of *L. sativa* in control plants (Ctrl) as compared to those supplemented with KIO_3_ and SA. In each combination, DEG enrichment was based on Kyoto Encyclopedia of Genes and Genomes (KEGG) pathway system.

Cross-reference of DEGs detected in the roots of lettuce plants treated with SA and KIO_3_ revealed 134 commonly regulated DEGs as well as 124 and 195 DEGs differentially expressed only under SA or KIO_3_ treatment, respectively (Fig. 1B). To identify genes possibly regulating the distinct distribution of KIO_3_ and SA, we identified DEGs by comparing their expression in leaves and roots (Supplementary Data S5–S7) and performed KEGG and GO enrichment analyses (Fig. 2, Table S1). The number of DEGs was the highest under KIO_3_ treatment (4763) (Supplementary Data S6). As a result of the KIO_3_ application, aminoacyl tRNA biosynthesis, carotenoid biosynthesis, starch and sucrose metabolism, chlorophyll metabolism, oxidative phosphorylation, tetrapyrrole metabolic process, chromatin assembly and cofactor metabolic process were the pathways comprising the most differentially expressed genes between leaves and roots (Fig. 2). Following the SA application, the enriched pathways included protein folding and export, pyrimidine metabolism, amide biosynthetic process, sulfate transport, and pathogen-associated molecular defense (Fig. 2).

### Quantitative real-time qPCR of DEGs

**Figure 3:**
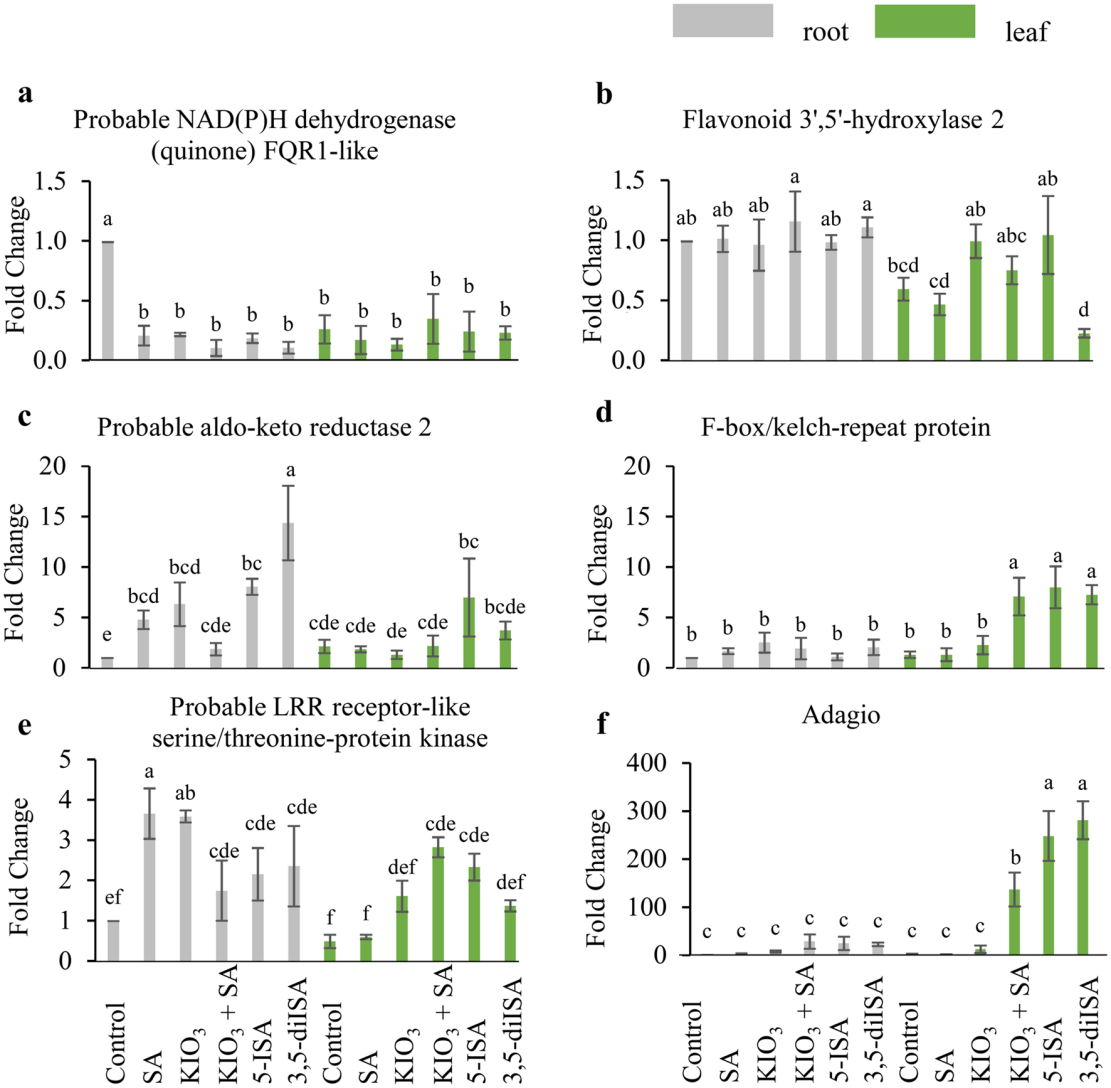
Expression profiles of probable NAD(P)H dehydrogenase (quinone) FQR1-like (**a**), flavonoid 3′5′-hydroxylase 2 (**b**), probable aldo–keto reductase 2 (**c**), F-box/kelch-repeat protein (**d**), probable LRR receptor-like serine/threonine-protein kinase (**e**) and Adagio protein 3 (**f**) genes in root and leaf tissues of *L. sativa* L. under control, SA, KIO_3_, KIO_3_ + SA, 5-ISA and 3,5-diISA. Data are expressed as the mean ± SD (standard deviation) of four independent biological replicates and three technical replications with *p* < 0.05 (Tukey’s post-hoc test). The same letters indicate no statistical differences between treatments.

Based on the results of the differential expression analysis, 6 genes potentially involved in iodine uptake, transport and metabolism were selected. A* probable NAD(P)H dehydrogenase (quinone) FQR1-like* gene was down-regulated in all experimental groups, as compared to that in the roots of lettuce plants grown in the control conditions (Fig. 3A), whereas down-regulation of *flavonoid 3',5'-hydroxylase 2* was detected only in roots of the control plants, SA and 3,5-diISA experimental groups (Fig. 3B). In the case of *probable aldo–keto reductase 2*, the highest up-regulation was observed in the roots of lettuce plants treated with 3,5-diISA (Fig. 3C). Application of KIO_3_ and SA separately caused up-regulation of *probable aldo–keto reductase 2* in the roots, however simultaneous application of those compounds did not modify the expression level of that gene. In lettuce leaves, enhanced expression of *F-box/kelch-repeat protein* was revealed only in plants treated with KIO_3_ + SA, 5-ISA, 3,5-diISA, whereas in the roots its expression level was not altered (Fig. 3D). A *probable LRR receptor-like serine/threonine protein kinase* was up-regulated in both lettuce roots and leaves from all treatments, except in leaves from plants treated with SA (Fig. 3E). Up-regulation of the *Adagio* gene was observed in leaves of KIO_3_ + SA, 5-ISA and 3,5-diISA treated lettuce plants (Fig. 3F).

### Biomass, uptake and distribution of iodine including organic iodine compounds

The 3,5-diISA, 5-ISA and KIO_3_ + SA application, as compared to the control as well as separate applications of KIO_3_ and SA, caused a reduction in plant growth (Table S2). This was accompanied by the highest accumulation of total iodine in roots, leaves and secretions collected as a result of root pressure [RootSec] of plants grown on the medium supplemented with 3,5-diISA and 5-ISA (Table 2). Trace content of IO_3_^−^ (as compared to I^−^) was found in RootSec, roots and leaves, except for its increased accumulation in the leaves and roots of plants treated with 5-ISA. Application of 3,5-diISA and 5-ISA resulted in a significant increase in the accumulation of these iodosalicylates in RootSec, roots and leaves. At the same time, the content of I^−^ increased several times compared to the control or fertilization of plants only with KIO_3_. These results indicate that exogenous 3,5-diISA and 5-ISA are transported from the roots to leaves and that they undergo catabolism processes leading to the formation of inorganic iodine I^−^ and IO_3_^−^. This is also supported by an increased accumulation of SA in roots and leaves of plants grown in the presence of 3,5-diISA and 5-ISA (Table 2).

**Table 2:** Concentrations of iodine (total concentrations), iodides (I^−^), iodates (IO_3_^−^), salicylic acid (SA), 5-iodosalicylic acid (5-ISA), 3,5-diiodosalicylic acid (3,5-diISA), and chlorides (Cl^−^) in leaves and roots of lettuce as well as root secretions (RootSec).

Part of plant/RootSec*	Treatment	Iodine (total) (mg kg^−1^ D.W.)	Iodides (I^−^) (mg kg^−1^ D.W.)	Iodates (IO_3_^−^) (mg kg^−1^ D.W.)	SA (mg kg^−1^ D.W.)	5I-SA (mg kg^−1^ D.W.)	3,5diI-SA (mg kg^−1^ D.W.)	Chlorides/Cl^−^/(mg kg^−1^ D.W.)
			I^−^	IO_3_^−^				Cl^−^
		Leaves						
Leaves	Control	3.04a ± 0.405	2.338b ± 0.0049	0.0273b ± 0.00073	0.520a ± 0.0500	0.047b ± 0.0088	0.044a ± 0.0085	3 162.9ab ± 370.29
	SA	1.47a ± 0.143	0.970a ± 0.0069	0.0290c ± 0.00095	0.323a ± 0.0170	0.006a ± 0.0006	0.028a ± 0.0090	3 899.8c ± 158.95
	KIO_3_	18.24bc ± 0.911	15.063d ± 0.0423	0.0148a ± 0.00070	0.455a ± 0.0284	0.021ab ± 0.0027	0.195b ± 0.0687	3 046.4ab ± 348.52
	KIO_3_ + SA	24.32c ± 2.635	17.491e ± 0.1403	0.0140a ± 0.00000	0.407a ± 0.0404	0.009a ± 0.0004	0.024a ± 0.0069	2 876.9a ± 123.72
	5-Iodosalicylic acid	138.41d ± 3.641	97.065f. ± 0.1893	0.0488d ± 0.00050	1.096b ± 0.0809	1.557d ± 0.0855	0.014a ± 0.0033	3 424.0b ± 205.39
	3,5-Diiodosalicylic acid	14.84b ± 2.349	8.625c ± 0.0443	< LOQ	1.037b ± 0.1189	0.385c ± 0.0179	12.679c ± 2.9463	2 951.3a ± 143.89
		Roots						
Roots	Control	19.9a ± 6.78	7.70a ± 0.019	0.878d ± 0.0007	1.45a ± 0.324	0.031a ± 0.0034	0.745a ± 0.2391	760.9b ± 102.20
	SA	30.9a ± 10.76	0.16b ± 0.002	0.014b ± 0.0001	2.05a ± 0.643	0.029a ± 0.0009	0.469a ± 0.1061	734.9b ± 132.03
	KIO_3_	67.1a ± 9.77	10.87c ± 0.013	0.991a ± 0.0006	1.99a ± 0.515	0.027a ± 0.0039	0.321a ± 0.0504	603.5ab ± 116.94
	KIO_3_ + SA	93.6a ± 16.75	34.99d ± 0.405	0.244c ± 0.0037	3.03a ± 0.990	0.030a ± 0.0060	0.068a ± 0.0069	530.3a ± 86.98
	5-Iodosalicylic acid	699.1c ± 47.63	72.58e ± 0.076	7.780e ± 0.0428	10.83b ± 3.889	10.957b ± 2.7679	11.983b ± 4.3176	772.3b ± 167.92
	3,5-Diiodosalicylic acid	520.5b ± 123.35	8.12a ± 0.018	0.959a ± 0.0000	14.20b ± 4.213	2.531a ± 0.6405	595.896c ± 99.7866	452.8a ± 46.02

		RootSec*						
		Iodine (total) (µg I dm^−3^)	Iodides (I^−^) (µg I dm^−3^)	Iodates (IO_3_^−^) (µg I dm^−3^)	SA (µg dm^−3^)	5I-SA (µg dm^−3^)	3,5diI-SA (µg dm^−3^)	Chlorides/Cl^−^/ (µg dm^−3^)
RootSec	Control	No data	16.1a ± 0.25	1.08b ± 0.07	638.6b ± 10.0	8.4a ± 0.82	< LOQ	No data
	SA	No data	10.4a ± 0.37	0.74a ± 0.15	214.6a ± 18.3	8.3a ± 0.57	< LOQ	No data
	KIO_3_	No data	184.3b ± 3.69	2.02c ± 0.05	2 163.0d ± 100.0	8.7a ± 0.73	< LOQ	No data
	KIO_3_ + SA	No data	215.2bc ± 19.76	2.94d ± 0.71	118.2a ± 18.9	7.2a ± 0.24	< LOQ	No data
	5-iodosalicylic acid	No data	1789.9d ± 18.29	0.70a ± 0.11	740.6c ± 4.0	386.1c ± 11.45	< LOQ	No data
	3,5-diiodosalicylic acid	No data	291.2c ± 23.36	0.67a ± 0.02	258.4a ± 51.1	58.6b ± 0.67	63.9 ± 6.07	No data

*RootSec—Results of the determination of individual compounds in secretions collected as a result of root pressure– this is in white secretion on the surface of the root neck after cutting the heads (lettuce leaves). < LOQ—Below limit of quantification (LOQ). Means in the column followed by different letters differ significantly at P < 0.05 (n = 8; means from two cultivation cycles).

Application of 3,5-diISA, 5-ISA and KIO_3_ had no negative effect on the uptake and accumulation of Cl^−^/chloride ion/ (Table 2). Only after SA application was the leaf content of Cl^−^ higher than in the control. In addition, exogenous 5-ISA caused the highest accumulation of several organic iodine metabolites in the roots, namely: 2-IBeA/2-iodobenzoic acid/ (Table 3), 5,7-diiodo-8-quinolinol (Table S3) as well as T4 in RootSec (Table 3).

**Table 3:** Concentrations of benzoic acid (BeA), 2-iodobenzoic acid (2-IBeA), 4-iodobenzoic acid (4-IBeA), 2,3,5-triiodobenzoic acid (2,3,5-triIBeA), iodotyrosine (I-Tyr), triiodothyronine (T3), reversed triiodothyronine (rT3) and thyroxine T4 in leaves and roots of lettuce as well as root secretions (RootSec).

Part of plant/RootSec*	Treatment	BeA (mg kg^−1^ D.W.)	2I-BeA (mg kg^−1^ D.W.)	4I-BeA (mg kg^−1^ D.W.)	2,3,5-triIBeA (mg kg^−1^ D.W.)	Iodotyrosine (mg kg^−1^ D.W.)	3,3′,5-triiodothyronine /T3/ (mg kg^−1^ D.W.)	3,3′,5′-Triiodothyronine /rT3/ (µg dm^−3^)	Thyroxine T4 (mg kg^−1^ D.W.)
							
		Leaves							
Leaves	Control	6.69a ± 1.611	0.281b ± 0.0806	0.0245a ± 0.00858	0.0050a ± 0.00122	0.268b ± 0.0219	0.340b ± 0.0263	0.0075b ± 0.0003	< LOQ
	SA	5.39a ± 1.791	0.039a ± 0.0073	0.0229a ± 0.00986	0.0052a ± 0.00081	0.029a ± 0.0031	0.174ab ± 0.0500	0.0008a ± 000,003	< LOQ
	KIO_3_	7.61b ± 1.635	0.094a ± 0.0060	0.0172a ± 0.00659	0.0090a ± 0.00173	0.560c ± 0.0921	0.1268a ± 0.0483	0.0149c ± 0.00113	< LOQ
	KIO_3_ + SA	7.43ab ± 1.538	0.022a ± 0.0013	0.0249a ± 0.00849	0.0093a ± 0.00138	0.061a ± 0.0088	0.254ab ± 0.0375	0.0024a ± 0.00009	< LOQ
	5-Iodosalicylic acid	6.68a ± 1.723	0.027a ± 0.0034	0.0262a ± 0.00964	0.0072a ± 0.00071	0.053a ± 0.0069	0.257ab ± 0.0843	0.0092b ± 0.00092	< LOQ
	3,5-diiodosalicylic acid	6.22a ± 1.007	0.368c ± 0.1260	0.0288a ± 0.00930	0.0079a ± 0.00167	0.034a ± 0.0030	0.643c ± 0.1114	0.0176c ± 0.00184	< LOQ
		Roots							
Roots	Control	3.53ab ± 0.596	0.039a ± 0.0014	0.020ab ± 0.0065	0.007a ± 0.0011	0.045ab ± 0.0052	13.97a ± 3.226	0.046a ± 0.0045	< LOQ
	SA	3.33a ± 0.561	0.029a ± 0.0020	0.011a ± 0.0036	0.010a ± 0.0032	0.050b ± 0.0021	22.28bc ± 7.255	0.096b ± 0.0219	< LOQ
	KIO_3_	3.87ab ± 1.266	0.039a ± 0.0025	0.044b ± 0.0161	0.006a ± 0.0016	0.086c ± 0.0034	16.62a ± 5.045	0.049a ± 0.0134	< LOQ
	KIO_3_ + SA	4.42ab ± 0.661	0.034a ± 0.0033	0.020ab ± 0.0078	0.008a ± 0.0010	0.093c ± 0.0080	21.77b ± 6.588	0.054a ± 0.0046	< LOQ
	5-Iodosalicylic acid	3.45ab ± 0.473	0.093b ± 0.0073	0.021ab ± 0.0073	0.008a ± 0.0010	0.090c ± 0.0106	19.32ab ± 5.484	0.084ab ± 0.0153	< LOQ
	3,5-Diiodosalicylic acid	6.87b ± 2.027	0.039a ± 0.0093	0.014a ± 0.0033	0.209b ± 0.0456	0.027a ± 0.0049	22.87c ± 5.374	0.048a ± 0.0078	< LOQ

		RootSec*							
		BeA (µg dm^−3^)	2I-BeA (µg dm^−3^)	4I-BeA (µg dm^−3^)	2,3,5-triIBeA (µg dm^−3^)	Iodotyrosine (µg dm^−3^)	3,3′,5-Triiodothyronine/T3/ (µg dm^−3^)	3,3′,5′-Triiodothyronine/rT3/ (µg dm^−3^)	Thyroxine T4 (µg dm^−3^)
RootSec	Control	68 ± 9.0b	0.05ab ± 0.01	0.04a ± 0.01	0.25a ± 0.01	0.52b ± 0.08	484.1ab ± 40.5	2.81b ± 0.10	0.22ab ± 0.01
	SA	98 ± 0.6c	0.15c ± 0.01	0.19c ± 0.01	2.67c ± 0.46	0.56b ± 0.02	422.4ab ± 41.6	1.31a ± 0.01	1.83c ± 0.10
	KIO_3_	116 ± 9.9 cd	0.07b ± 0.01	0.10b ± 0.01	0.45b ± 0.10	0.55b ± 0.06	746.7c ± 58.6	2.84b ± 0.10	0.01a ± 0.00
	KIO_3_ + SA	88 ± 10.0bc	0.26d ± 0.03	0.08b ± 0.01	0.17a ± 0.07	0.71c ± 0.06	608.9bc ± 77.5	3.46c ± 0.10	0.64b ± 0.09
	5-Iodosalicylic acid	2 ± 1.0a	0.02a ± 0.01	0.06ab ± 0.01	0.01a ± 0.002	0.52b ± 0.24	342.0ab ± 3.4	5.00d ± 0.06	4.78d ± 0.10
	3,5-Diiodosalicylic acid	128 ± 10.0d	0.01a ± 0.00	0.06ab ± 0.01	0.09a ± 0.01	0.16a ± 0.01	285.2a ± 36.1	2.10b ± 0.10	4.04d ± 0.15

*RootSec—Results of the determination of individual compounds in secretions collected as a result of root pressure—this is in white secretion on the surface of the root neck after cutting the heads (lettuce leaves). < LOQ—Below limit of quantification (LOQ). Means in the column followed by different letters differ significantly at P < 0.05 (n = 8; means from two cultivation cycles).

The application of exogenous 3,5-diISA resulted in: A) the highest level of the following organic iodine compounds in the roots: 2,3,5-triIBeA/2,3,5-triiodobenzoic acid/, T3 (Table 3) and 5-chloro-7-iodoquin-8-ol (Table S3); (B) the highest accumulation in the leaves of: 2-IBeA, T3 (Table 3), 6-iodo-4-hydroxy-3-quinoline carbocyclic acid, 7-iodo-4-hydroxy-3-quinoline carbocyclic acid and 8-iodo-4-hydroxy-3-quinoline carbocyclic acid (Table S3); (C) the highest content in RootSec of: T4 (Table 3), 5-chloro-7-iodoquin-8-ol, 5,7-diiodo-8-quinolinol and 7-iodo-4-hydroxy-3-quinoline carbocyclic acid (Table S3).

### The effect of the application of tested compounds on the content of primary and secondary metabolites

The application of 3,5-diISA resulted in the highest content of: SA (Table 2) in leaves and roots; BeA in the roots (Table 3); quinoline in leaves (Table S3); chlorogenic, ferulic, p-coumaric and sinapic acids in leaves (Table S4); vitamin B3 and C in leaves (Table S5); fructose (F), glucose (G), sucrose (S), and sum of sugars (F + G + S) in leaves (Table S6); the highest degree of FRAP, ABTS and DPPH antioxidant activity in leaves (Table S6); and the highest content of dietary fiber in leaves (Table S7).

Supplying plants with exogenous 5-ISA resulted in the highest accumulation of: SA in leaves (Table 2), hydroxychloroquine in RootSec and roots (Table S3). That treatment also increased the content of p-coumaric and chlorogenic acid (Table S4) as well as dietary fiber in lettuce leaves as compared to controls (Table S7).

Fertilization with KIO_3_ resulted in the highest content of: (A) SA (Table 3), BeA (Table 3) in RootSec and (B) hydroxychloroquine (Table S3); vitamins: B6, B5, B3, B1 (Table S5) as well as the content of ash in lettuce leaves (Table S7).

### Effect of tested compounds on the uptake of macroelements and microelements

Compared to the control and other tested combinations, fertilization of plants with KIO_3_ alone resulted in the highest content of ammonium and nitrate(V) ions in leaves (Table S8), and N-total in leaves and roots (Table S9). Simultaneous application of KIO_3_ + SA increased the content of nitrate(V) in both leaves and roots as along with the highest leaf accumulation of nitrates(III) (Table S8), Mg, K, Ca (Table S9) and Mn (Table S10) in leaves as well as root content of Fe (Table S10).

The application of 3,5-diISA resulted in obtaining the highest content of Mg, Na (Table S9) and B (Table S10) in the roots as well as Fe and B in the leaves (Table S10).

## Discussion

### Iodine uptake, transport and metabolism versus uptake of chloride and iodosalicylate metabolism

After the application of KIO_3,_ the content of Cl^−^ ion in the leaves and roots was at a similar level as in the control plants. Meanwhile, the analysis of RNA-seq data showed that after the root application of KIO_3_, genes related to chloride transmembrane transport process (GO:1902476) were up-regulated. These results are only seemingly contradictory. First of all, before entering the root cell, iodate ions are reduced into the iodides and mostly in that form are taken up by plants^17^. What is more, it is generally recognized that the transport of iodides (I^−^) in the root cells takes place in the same way as Cl^−^ transport, i.e. by ion channels capable of transmitting Cl^−^/I^−^ through H^+^/anion or symporters of Na:K/Cl^18,19^ and the above-mentioned observation provides further substantiation to that issue. The increased content of I^−^ in the RootSec, roots and leaves of plants with exogenously applied 3,5-diISA and 5-ISA indicates that these two iodosalicylates were converted in the roots into I^−^ and in that form transported to the leaves. The obtained results additionally suggest that the catabolism of exogenous 3,5-diISA and 5-ISA, e.g. to I^−^ can also take place in the leaves.

**Figure 4:**
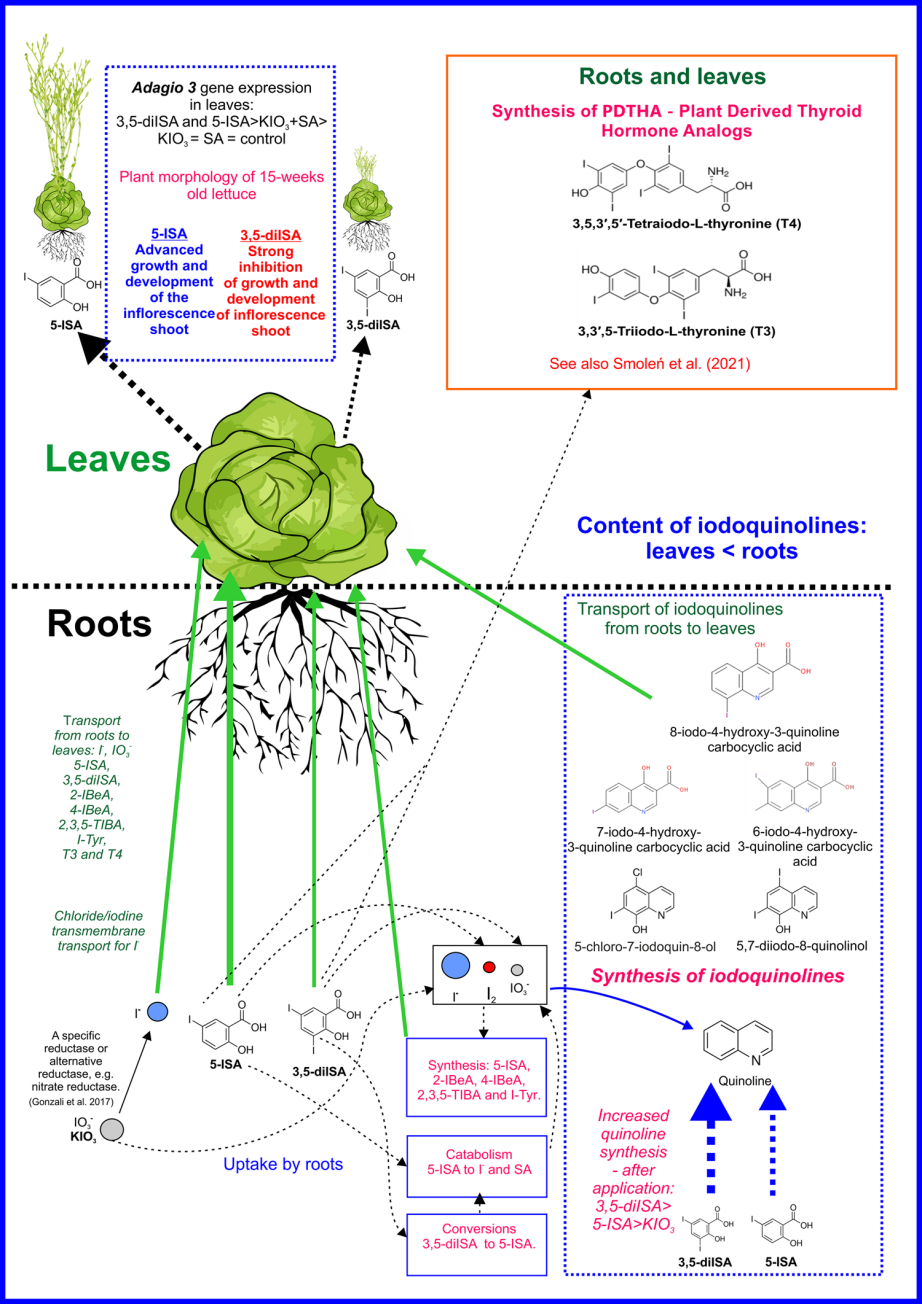
Partial graphical summary results of the study and literature data—in aspect of uptake, transport (IO_3_^−^ and iodosalicylates), outline gens expression (probable *NAD(P)H dehydrogenase (quinine) FQR1-like* and *Adagio)* as well as selected metabolic pathways of iodine compounds in lettuce plants.

In plants, long-distance transport of free SA takes place mainly through the phloem^20^, while within tissues SA movement occurs both through apoplast^21^ or symplast^22^. Transport of SA and its metabolites via xylem is also possible^23^. From cell to cell or within cells through cytoplasmic membranes, transport of SA or its glucoside and methyl derivatives takes place through an H^(+)^-antiport-type mechanism^24^. In our study, the transport of 3,5-diISA, 5-ISA and SA from the roots to the leaves was probably passive along with the transport of water in xylem bundles or through apoplastic and/or symplastic transport. This is indicated by the significantly increased 3,5-diISA and 5-ISA content in RootSec for 3,5-diISA, 5-ISA and SA treated plants, respectively. A root-to-leaf transport of primary and secondary metabolites (including those derived from quinoline as well as other organic metabolites of iodine) may occur in the similar manner. It is supported by the increased content of various metabolites (including: BeA, 2,3,5-triIBeA, 4-IBeA, 2-IBeA, T4, T3, rT3, iodotyrosine, quinoline and its derivatives) in the RootSec of plants treated with iodine compounds or SA as compared to the control (Tables 3 and Table S3). A graphical presentation of a proposed model of iodine transport and synthesis of iodine metabolites is shown in Fig. 4.

### Gene expression of *probable NAD(P)H dehydrogenase (quinone) FQR1-like*, *flavonoid 3',5'-hydroxylase 2 and F-box/kelch-repeat protein versus* iodosalicylate metabolism and others metabolites

The *probable NAD(P)H dehydrogenase (quinone) FQR1-like* gene encodes a protein that catalyzes the transfer of electrons from NADH or NADPH to several quinones (https://www.uniprot.org/uniprotkb/Q9LSQ5/entry). Enzymes from this group of dehydrogenase FMN-binding proteins form homodimers and catalyze two-electron reduction of quinones to hydroquinones^25^. In our study, after the application of iodine or SA compounds, a significant increase in the content of at least a few metabolites containing one or more aromatic rings in the structure, or its derivatives, was noted including: SA and iodosalicylates (3,5-diISA, 5-ISA), BeA and iodobenzoates (4-IBeA, 2,3,5-triIBeA), I-Tyr, T4, T3, rT3, quinoline and its derivatives, phenolic acids as well as vitamins: B2, B3, B9 and PP in lettuce leaves, roots or RootSec. Meanwhile, qPCR results confirmed a significant reduction in the expression of the *probable NAD(P)H dehydrogenase (quinone) FQR1-like* gene in lettuce roots and leaves from all combinations as compared to the control. It may have been due to the gene silencing by high concentrations of some of the primary and secondary metabolites mentioned—including exogenous organic iodine compounds.

In the roots, application of KIO_3_ increased the expression of genes attributed to phenylpropanoid metabolic process (GO:2000762). The enzyme encoded by the *flavonoid 3',5'-hydroxylase 2* gene participates in the conversion of flavanones into 3'-hydroxyflavanone and 3′,5′-dihydroxyflavanone^26,27^. SA induces flavonoid biosynthesis pathways^28^. The qPCR analysis for the *flavonoid 3',5'-hydroxylase hydroxylase 2* gene showed no significant changes in its expression in the roots following the application of SA or iodine compounds. It may be due to the fact that, as already demonstrated in some plants, root activity of that gene is very low^29,30^. At the same time, the application of KIO_3_ and 5-ISA increased, and the application of 3,5-diISA decreased the expression of the *flavonoid 3',5'-hydroxylase 2* gene in the leaves *vs* control plants. In our view, this could be caused by a specific regulation of the expression of this gene by accumulated 3,5-diISA, 5-ISA (or other iodine forms in leaves). A specific inhibiting effect of 3,5-diISA on the expression of *flavonoid 3',5'-hydroxylase 2* in leaves somehow correlates with the results of the analysis of antioxidant status of lettuce plants. The leaves of plants treated with 3,5-diISA showed the highest anti-radical activity measured by FRAP, ABTS and DPPH methods (Table S6). The antioxidant status of lettuce plants is related, inter alia, to the content of flavonoids and their metabolites^28^. Also the exposure to various stress conditions significantly modifies the antioxidant potential of higher plants^31^. At the same time, *flavonoid-3*′*5*′* hydroxylase* gene expression has also been reported to be affected by various abiotic and biotic stress factors as well as phytohormones such as SA, abscisic acid and methyl jasmonate. In the last case, the expression of *flavonoid 3',5'-hydroxylase hydroxylase 2* was either up- or down-regulated depending on the applied compound^32^. Taking this into account, it can be hypothesized that from all used iodine compounds, exogenous 3,5-diISA has most distinguishingly affected the antioxidant status of lettuce. It seems of particular interest in terms of the previously hypothesized role of iodosalicylates in improving the health-promoting properties of biofortified plants^33^. Down-regulation of *Flavonoid 3',5'-hydroxylase 2* in leaves after 3,5-diISA application could be due to the feedback mechanism related to the accumulation of large amounts of various secondary metabolites in leaves, i.e. T3, rT3, SA, 2-IBeA, BeA, quinoline and its derivatives, l-ascorbic acid, vitamin B3, chlorogenic, sinapic, p-coumaric, ferulic and 3-hydroxybenzoic acids.

F-box proteins are responsible for the regulation of many developmental processes and the course of plant metabolism^34^. They also regulate the synthesis of phenylpropanoids^35^, with coumaric acid being the major intermediate in that process. A phenylpropanoid pathway is involved in the biosynthesis of lignols (precursors of lignin and lignocellulose), isoflavonoids, flavonoids, coumarins and many other metabolites^36^. The qPCR results showed increased expression of the *F-box/kelch-repeat protein* gene only in leaves of plants treated with 3,5-diISA, 5-ISA and KIO_3_ + SA, which was accompanied by the increased content of the total iodine but also the I^−^ concentration in the leaves. Blasco et al.^37,38^ showed that iodine increased the content of flavonoids, anthocyanins and phenolic compounds in leaves of lettuce. In our study, higher expression of the *F-box/kelch-repeat protein* gene in the leaves of plants treated with 3,5-diISA, 5-ISA and KIO_3_ + SA, was also positively correlated with the content of SA, *p*-coumaric acid, sinapic acid, chlorogenic acid as well as iron, which is a cofactor of many plant enzymes.

### Gene expression of *aldo–keto reductase 2* and *probable LRR receptor-like serine/threonine-protein kinase* and their possible functional significance

T4 and T3 are well-known hormones that are produced by the thyroid gland and play a major role in regulating the functioning of human and animal organisms. PDTHAs, e.g. T4 and T3, are a very poorly studied group of plant metabolites. Their function in plants is unknown, however the existence of possible T4 and T3 receptors^39^ or transport proteins in cells of higher plant has been hypothesized^40^. Our previous research^4^ described the potential metabolic pathway of PDTHAs in lettuce, that may occur in roots rather than leaves, with 3,5-diISA and 5-ISA functioning as substrates. In addition, it has been documented that in lettuce the *cipk6* gene encodes a protein that is likely to function as a T3/T4 receptor^4^. In the studies on the functional properties of PDTHAs, researchers look for biochemical and molecular similarities between the plant and animal/human organisms^7,40^. The currently presented analysis of RNA-seq data regarding the synthesis, physiological or biochemical function of PDTHAs was hardened by the lack of reference entries in respective databases that may relate any lettuce, or plant genes in general, to that group of metabolites. Therefore, it was chosen to apply a literature search strategy in order to identify similarities between specifically overexpressed genes in lettuce and human genes encoding proteins engaged directly in T4 or T3 synthesis or acting as their receptors and transporters. Two genes with differential expression (DEGs) (KIO_3_ vs. Ctrl in roots) were selected for qPCR analysis, namely: *aldo–keto reductase 2* gene (XLOC_003743, log_2_FC = 2.6) and *probable LRR receptor-like serine/threonine-protein kinase* (XLOC_007779, log_2_FC = 1.1). In the human body, genes from the *aldo–keto reductase* family are involved in the response of cells to thyroid hormones^41,42^. The results of the qPCR analysis showed that the expression of *aldo–keto reductase 2* in the roots and leaves of the lettuce plants treated with 3,5-diISA and 5-ISA was higher than in other treatments. It has been revealed that the expression of *aldo–keto reductase 2* gene is positively correlated with the content of iodine and its organic metabolites in RootSec, leaves and roots. That observation provides the basis for the *aldo–keto reductase 2* gene to be assigned to the functional response of plants to iodine and its organic metabolites, including PDTHAs.

In the human body, proteins encoded by genes from the *serine/threonine-protein kinase* family are activated by thyroid hormones and involved in many cellular processes^43,44^. In *A. thaliana*, genes from the *probable LRR receptor-like serine/threonine-protein kinase* family are engaged in many metabolic pathways as well as energy conversion i.a. conditioned by phosphorylation^43^. The role of receptor-like protein kinases in plants is also recognized for such processes as: senescence, stress response and signal transduction^45^. The results of the qPCR analysis showed that in lettuce roots, the expression of the *probable LRR receptor-like serine/threonine-protein kinase* gene was significantly higher in all treatments as compared to the control. In the leaves, all iodine treatments led to increased expression of that gene. Comparing the qPCR results with the metabolomic data, it can be proposed that in lettuce the respective gene product might be involved in general plant response to the application of exogenous iodine, irrespective of its chemical form (3,5-diISA, 5-ISA, SA, KIO_3_ + SA and KIO_3_).

### The *Adagio 3* gene and the initiation of the flowering process

Lettuce initiates flowering and produces an inflorescence shoot under photoperiod conditions > 12 h^46^. In the studies by Smoleń et al.^16^, exogenous 5-ISA stimulated the growth of the inflorescence shoot in lettuce at winter photoperiod < 10 h; such effect was not observed for 3,5-diISA. In the current studies, we included the evaluation of the expression of the *Adagio 3* gene, as it plays a role in the initiation of flowering in plants in conjunction with the photoperiod and the circadian rhythm^47^. We aimed at determining whether iodine application affects the molecular regulation of plant flowering in the spring and summer cultivation (> 12 h photoperiod). The transcriptomic analysis revealed an increased expression of genes related to ath04712 (circadian rhythm—plants) and rhythmic process (GO:0048511) in plants. In addition, a significant increase in the expression of the *Adagio 3* gene in leaves occurred after the application of: 3,5-diISA and 5-ISA > KIO_3_ + SA > other treatments and control. Thus, only the application of both iodosalicylates and KIO_3_ + SA induced lettuce flowering. The lack of an increase in *Adagio 3* activity after the application of SA alone is unexpected as SA has been shown to stimulate flowering in finger millet plants^48^. It is also surprising that *the Adagio 3* gene was not induced after the application of KIO_3_ alone, as^49^ found that KI increased the number of pelargonium inflorescences. Furthermore, Kiferle et al.^1^ showed that the application of IO_3_^−^ and I^−^ ions initiated the flowering of *A. thaliana*.

The levels of *Adagio 3* gene expression noted for 3,5-diISA and 5-ISA applications are not quite consistent with morphological observations on the initiation of inflorescence shoot formation in lettuce grown during a short photoperiod (as previously reported by Smoleń et al.)^16^. In order to evaluate the effect of 3,5-diISA and 5-ISA on inflorescence, a dozen lettuce plants were left for further cultivation (spring and summer season; > 14-h photoperiod) after the harvest and sample collection. It has been revealed that 5-ISA accelerated the production of inflorescence shoots. In turn, exogenous 3,5-diISA prevented or significantly delayed the initiation and/or growth of inflorescence shoots (Fig. S2). Therefore, 3,5-diISA, despite the increased expression of *Adagio 3* (at a level similar to 5-ISA), ultimately inhibited the flowering of lettuce. Probably the increased activity of the *Adagio 3* gene after the application of 3,5-diISA was due to its partial catabolism to 5-ISA (metabolic process stated by Smoleń et al.^4^). Detailed explanation of the influence of 3,5-diISA and 5-ISA on the flowering process of lettuce requires further research in this field.

To sum up, molecular studies, mostly including transcriptomic analysis, allowed to characterize diverse plant response in terms of differentiated gene regulation in lettuce roots and leaves under the influence of exogenous KIO_3_ and SA. In roots 329 differentially expressed genes (DEGs) were detected after application of KIO_3_, out of which 252 genes were up-regulated, and 77 were down-regulated. In leaves, only 9 genes revealed differential expression pattern. DEGs analysis indicated the involvement of iodine in such metabolic pathways and processes as: chloride transmembrane transport, phenylpropanoid metabolism, positive regulation of defense response and leaf abscission, and also ubiquinone and other terpenoid-quinone biosynthesis, protein processing in endoplasmic reticulum, circadian rhythm including flowering induction. It needs to be underlined that the application of bioinformatics approach (de novo transcriptome assembly) allowed to functionally annotate only these genes, that were previously described in the databases. So far, the processes of the uptake, transport and metabolism of organic iodine compounds in higher plants are weakly described so the observed effects regarding the differentiated gene expression cannot be linked to exact metabolic processes. The complex analysis of chemical composition of lettuce plants allowed however to indicate the synthesis of which primary and secondary metabolites was affected by exogenous iodine compounds and SA. That became the basis for the selection of putative genes coding proteins that may have been engaged in plant response to the applied iodine compounds. The range of conducted studies allowed to widen the knowledge as compared to our previous report in that area^4^. The occurrence of PDTHAs as endogenous metabolites in lettuce has been confirmed. For the first time, the presence of novel and yet undescribed group of endogenous iodine compounds, i.e. iodoquinolines has been detected. Characterisation of a possible physiological role of PDTHAs, iodoquinolines and other organic iodine compounds in higher plants requires, however, further detailed studies.

## Materials and methods

### Plant material and treatments

Lettuce cv. 'Melodion' (*L. sativa* L. var. *capitata*) was grown in a NFT hydroponic study. The experiment was located in a greenhouse (50° 05′ 04.1″ N 19° 57′ 02.1″ E) and was repeated twice in the spring season in two separate years on the campus of the University of Agriculture in Kraków, Poland. In both years of the study, seeds were sown in early March into 112-cell propagation trays with 32 × 32 × 40 mm sized cells filled with peat substrate mixed with sand (1:1 v/v). Seedlings in phase 4–5 true leaves were transplanted into the hydroponic NFT system. Before replanting of the seedlings, substrate from between the roots was removed by rinsing with tap water. The plants were placed into holes (spaced 25 cm apart) of styrofoam slabs filling—the "dry hydroponic" method of cultivation without substrate has been used in order to obtain at harvest the entire undamaged root system (for molecular and chemical analyses).

During cultivation plants were watered during the day between 5.00 and 19.00 and during the night between 1.00 and 2.00 in cycles: 1 min at 5-min intervals. The nutrient solution (pH 5.70 and EC 1.75 mS cm^−1^) used for the lettuce cultivation contained macro- and microelements in the concentrations proposed by Smoleń et al.^50^.

Studies included the introduction of I and SA (Table 4) into the nutrient solution in the following treatments: (1) Control; (2) SA; (3) KIO_3_; (4) KIO_3_ + SA; (5) 5-iodosalicylic acid (5-ISA); (6) 3,5-diiodosalicylic acid (3,5-diISA). Application of tested compounds started at the rosette stage (5–6 true leaves). The 10 µM concentrations were used for all I compounds (molar mass equivalents) and SA. The nutrient solution (containing all tested compounds) was applied continually until harvesting of the plants.

**Table 4:** Dose of SA and I compounds in the hydroponics cultivation.

Treatments	Dose of I compounds and dose of I	Dose of SA	I and SA application from the rosette phase
Control	0.0204 µM I*	–	–
SA	0.0204 µM I*	10 µM	Permanent
KIO_3_	10 µM [10 µM I]	–	As above
KIO_3_ + SA	10 µM [10 µM I]	10 µM	As above
5-ISA	10 µM [10 µM I]	–	As above
3,5-diISA	10 µM [20 µM I]	–	As above

*Trace concentration of I in nutrient solution (I from fertilizers and tap water).

The experiment consisted of 4 replicates in a randomized block design. There were 15 plants per each replicate and 60 plants per one combination (totally 360 plants per experiment).

The samples of leaves and roots for molecular tests and chemical composition analysis were taken at the end of plant growth, when lettuce heads were well-formed. Thus, the results reflect the molecular, biochemical and physiological response of the lettuce plants to the continuous presence of SA and iodine compounds in the medium. Root and leaf transcriptome analyzes were performed for lettuce plants from three of the six test treatments: control, SA and KIO_3_. These treatments were selected to identify DEGs for the main research factors: iodine versus SA as a reference for both iodosalicylates. On the other hand, qPCR analyzes were carried out for selected DEGs in the roots and leaves of lettuce plants from all treatments. On the basis of bioinformatics analysis, key genes were selected that allowed verification of the physiological, biochemical and molecular functions of iodine in plants anionic form IO_3_^−^ versus 3,5-diISA and 5-ISA. RNA extraction from roots and leaves was conducted as described by Smoleń et al.^4^.

### Transcriptome analysis

RNA-seq was performed on RNA samples extracted from the roots and leaves representing three treatments: control (Ctrl), SA, KIO_3_. Total RNA isolation was performed with Direct-zol RNA MiniPrep Plus (Zymo Research, Irvine, CA, USA). All RNA extracts were treated with 1 U µl^−1^ RNase-free *Dnase* I (Thermo Fisher Scientific, Waltham, MA, USA) and 40 U µl^−1^ of RiboLock RNase Inhibitor (Thermo Fisher Scientific, Waltham, USA) to prevent DNA contamination. RNA integrity number (RIN) was determined by a Bioanalyzer 2100 (Agilent 2100 Bioanalyzer; Agilent Technologies, Palo Alto, Santa Clara, CA, USA). The cDNA libraries were prepared using the NEBNext^®^ Ultra™ RNA Library Kit (Illumina, San Diego, CA, USA). In total, 18 cDNA libraries, i.e., 2 tissues (leaf and root) × 3 treatments (Ctrl), SA, KIO_3_), × 3 experimental replicates were subjected to sequencing in PE mode, with 101 bp read length) on an Illumina HiSeq4000 (Illumina, San Diego, USA). The RNA-Seq datasets generated for this study were deposited in the NCBI under BioProject PRJNA891733.

Data filtering and quality control of reads were performed as described in Kęska et al.^51^. Based on the obtained reads, de novo transcriptomes were assembled using the StringTie ver. 1.3.3b^52^ with parameters described in Kęska et al.^51^. Then the mapped reads were quantified with RSEM ver. 1.3.0 program (https://deweylab.github.io/RSEM). Differential gene expression analysis using DeSeq2 ver. 1.18^53^ was performed to compare the transcriptomes of plant roots and leaves from three treatments. Transcripts with FDR (False Discovery Rate) < 0.05 were considered as significantly differentially expressed. Determination of the function of genes was established with Trinotate ver. 3.1.0 (https://trinotate.github.io), dedicated to de novo assembled ​​transcriptomes. topGO^54^ using Fisher's statistical test (p < 0.01) was used to identify enriched GO terms.

### Gene expression analysis

Based on the results of differential expression, six genes characterized by a different level of expression between the experimental groups were selected for qRT-PCR. qPCR primers were designed based on the assembled transcripts (Supplementary Table S11) using Primer3 (http://bioinfo.ut.ee/primer3-0.4.0/) and IDT PrimerQuest (https://eu.idtdna.com). qRT-PCR assay was performed as described in Smolen et al.^4^. Relative expression levels were calculated related to control samples from the root using the ∆∆Ct method^55^ with the standard curve and protein phosphatase 2A regulatory subunit A3 (pp2aa3) as the reference gene.

### Yield evaluation, chemical and biochemical analyzes of lettuce plant

Plants were harvested at the phase of lettuce head development, that is in mid-May. Then, the heads (lettuce leaves) were weighed and collected chemical and biochemical analyses. Leaf harvesting was immediately followed by pipette collection of RootSec [white secretion on the surface of the root neck, visible after cutting the heads at collar level—a secretion produced as a result of root pressure]. The RootSec was mixed with 20 mM Tris HCl buffer (pH 8.5). The secretion was collected for the determination of the chemical forms of I according to the method described by Smoleń et al.^4^. The collection of RootSec was followed by the measurement of lettuce root biomass. Chemical analyses were performed on RootSec leaves and roots of all plants each biological replicate.

The following chemical analyzes were performed: total iodine by ICP-MS/MS technique (iCAP TQ ICP-MS ThermoFisher Scientific, Bremen, Germany); IO_3_^−^ and I^−^ by HPLC-ICP-MS/MS; iodobenzoates, iodosalicylates, benzoic acid (BeA), SA, and plant-derived thyroid hormone analogs i.e. 2,3,5-triiodobenzoic acid (2,3,5-triIBeA), 4-iodobenzoic acid (4-IBeA), 2-iodobenzoic acid (2-IBeA), 3,5-diISA, 5-ISA, T3, reverse triiodothyronine (rT3), T4, iodotyrosine (I-Tyr)—the compounds were also measured in RootSec after lettuce harvesting. Iodine and all listed compounds were analyzed according to the method by Smoleń et al.^4^. The supporting information file presents the detailed methods and results of other chemical analyzed that were performed on the RootSec, leaf and root samples.

The obtained results of chemical analyzes were statistically verified using one-way analysis of variance (ANOVA) with the use of Statistica 12.0 PL software (https://www.tibco.com/products/data-science, StatSoft Inc., Tulsa, OK 74104, USA) at a significance level of P < 0.05. In the case of significant effects, homogenous groups were distinguished based on a post-hoc Tukey HSD test.

### Ethical statement

The study including the collection of plant material complies with relevant institutional, national, and international guidelines and legislation.

## Supplementary Information

Supplementary Information.

## Data Availability

The data used to support the findings of this study are available from the corresponding authors upon reasonable request.
