# Supplementary material for: Transcriptomic and metabolic studies on the role of inorganic and organic iodine compounds in lettuce plants
Source: Sci Rep. 2023 May 25;13:8440. doi: 10.1038/s41598-023-34873-7 (PMC10213046; doi:10.1038/s41598-023-34873-7)
Supplement: Supplementary file 1 — Supplementary Information. [file 41598_2023_34873_MOESM1_ESM.docx]

Supporting information file

**Transcriptomic and metabolic studies on the role of inorganic and organic iodine compounds in lettuce plants**

Sylwester Smoleń^1^, Małgorzata Czernicka^1*^, Kinga Kęska-Izworska^1^, Iwona Kowalska^1^, Dariusz Grzebelus^1^, Joanna Pitala^2^, Maria Halka^1^, Łukasz Skoczylas^3^, Małgorzata Tabaszewska^3^, Marta Liszka-Skoczylas^4^, Marlena Grzanka^1^, Iwona Ledwożyw-Smoleń^1^, Aneta Koronowicz^5^, Joanna Krzemińska^5^, Olga Sularz^5^, Daniel Kiełbasa^1^, Jakub Neupauer^6^, Peter Kováčik^6^

1 - Department of Plant Biology and Biotechnology, Faculty of Biotechnology and Horticulture, University of Agriculture in Krakow, al. Mickiewicza 21, 31-120 Krakow, Poland

2 - Laboratory of Mass Spectrometry, Faculty of Biotechnology and Horticulture, University of Agriculture in Krakow, al. Mickiewicza 21, 31-120 Krakow, Poland

3 - Department of Plant Product Technology and Nutrition Hygiene, Faculty of Food Technology, University of Agriculture in Krakow, University of Agriculture in Krakow, al. Mickiewicza 21, 31-120 Krakow, Poland

4 - Department of Engineering and Machinery for Food Industry, Faculty of Food Technology, University of Agriculture in Krakow, University of Agriculture in Krakow, al. Mickiewicza 21, 31-120 Krakow, Poland

5 - Department of Human Nutrition and Dietetics, Faculty of Food Technology, University of Agriculture in Krakow, University of Agriculture in Krakow, al. Mickiewicza 21, 31-120 Krakow, Poland

6 - Department of Agrochemistry and Plant Nutrition, Slovak University of Agriculture in Nitra, Tr. A. Hlinku 2, 949 01 Nitra, Slovakia

**List of tables in the supporting information file**

[**Table S1.** List of topGO and KEGG terms identified in tested comparisons. 2](#_Toc132621096)

[**Table S2.** Fresh weight of leaves / head/ and roots of lettuce 16](#_Toc132621097)

[**Table S3.** Concentrations of quinoline, 5-chloro-7-iodoquin-8-ol, 5,7-diiodo-8-quinolinol, hydroxychloroquine sulfate, 6-iodo-4-hydroxy-3-quinoline carbocyclic acid, 7-iodo-4-hydroxy-3-quinoline carbocyclic acid and 8-iodo-4-hydroxy-3-quinoline carbocyclic acid in leaves and roots of lettuce as well as root secretions.(RootSec). 17](#_Toc132621098)

[**Table S4.** Concentrations of chlorogenic, sinapic, p-coumaric, ferulic and 3-hydroxybenzoic acids in lettuce leaves. 18](#_Toc132621099)

[**Table S5.** Concentrations of water-soluble vitamins: B3 /nicotinic acid/, B7 /D-biotin/, PP /nicotinamide/, B5 /pantothenic acid/, B6 /pyridoxine/B2 /riboflavin/, B9 /folic acid/, B1 /thiamine/ and L-ascorbic acid /vitamin C/ in lettuce leaves. 19](#_Toc132621100)

[**Table S6.** Concentrations of sucrose (S), glucose (G), fructose (F) and sum of sugars (S+G+F), and free amino acids; activity of peroxidase (POX), catalase /CAT/, guaiacol peroxidase /POX/ as well as anti-radical activity DPPH, ABTS and FRAP in lettuce leaves. 20](#_Toc132621101)

[**Table S7.** Concentrations of ash, crude fat, protein, dietary fiber and digestible carbohydrates in lettuce leaves. 21](#_Toc132621102)

[**Table S8.** Concentrations of ammonium ion, nitrates(V) and nitrates(III) in leaves and roots of lettuce. 22](#_Toc132621103)

[**Table S9.** Concentrations of N, P, K, Mg, Ca, S and Na in leaves and roots of lettuce. 23](#_Toc132621104)

[**Table S10.** Concentrations of B, Cu, Fe, Mn, Mo and Zn in leaves and roots of lettuce. 24](#_Toc132621105)

[**Table S11.** Genes data and designed primers based on RNA-seq data used in the qRT-PCR analyses. 25](#_Toc132621106)

**List of figure in the supporting information file**

[**Figure S1.** Summary of de novo transcriptome assembly of Lactuca sativa L.: A) Numerical summary of the assembled transcriptome; B) Histogram showing distribution of the assembled transcripts; C) Pie chart showing distribution of homologous transcripts across species; D) UpSet plot displaying annotation of lettuce transcripts, according to the results from NCBI (Blastx, Blastp), GO, Pfam, KEGG and eggnog databases. 15](#_Toc132144042)

[**Figure S2.** Lettuce plants left to grow after harvesting the heads. The appearance of plants on 36 days after harvesting heads and roots for biomass assessment and chemical plant analysis. 26](#_Toc132144043)

**Table S1.** List of topGO and KEGG terms identified in tested comparisons.

| topGO | | | **R-KIO_3_ vs R-Ctrl** | | | | | | | | | | | | | | | | | | | | | | | | | | | | | | | | | | | | | | | | | | | | | | | | | | | | | | | | | | | | | | | | | | | | | | | | | | | | | | | | | | | |
| --- | --- | --- | --- | --- | --- | --- | --- | --- | --- | --- | --- | --- | --- | --- | --- | --- | --- | --- | --- | --- | --- | --- | --- | --- | --- | --- | --- | --- | --- | --- | --- | --- | --- | --- | --- | --- | --- | --- | --- | --- | --- | --- | --- | --- | --- | --- | --- | --- | --- | --- | --- | --- | --- | --- | --- | --- | --- | --- | --- | --- | --- | --- | --- | --- | --- | --- | --- | --- | --- | --- | --- | --- | --- | --- | --- | --- | --- | --- | --- | --- | --- | --- | --- | --- | --- | --- |
|  |  |  | Up-regulated | | | | | | | | | | | | | | | | | | | | | | | | | | | | | | | | | | | | | | | | | | | | | | | | | | | | | | | | | | | | | | | | | | | | | | | | | | | | | | | | | | | |
|  |  |  |  | | | | | | GO.ID | | | | | | | | | Term | | | | | | | | | | | | | | | | | | | | | | | | | | | Annotated | | | | | | | | | | Significant | | | | | | | | | | | Expected | | | | | | | classicFisher | | | | | | | | | | | | | q_value |
|  |  |  | BP | | | | | | GO:2000014 | | | | | | | | | regulation of endosperm development | | | | | | | | | | | | | | | | | | | | | | | | | | | 5 | | | | | | | | | | 3 | | | | | | | | | | | 0.06 | | | | | | | 2.10E-05 | | | | | | | | | | | | | 0.0006 |
|  |  |  |  |  |  |  |  |  | GO:1902476 | | | | | | | | | chloride transmembrane transport | | | | | | | | | | | | | | | | | | | | | | | | | | | 6 | | | | | | | | | | 3 | | | | | | | | | | | 0.08 | | | | | | | 4.20E-05 | | | | | | | | | | | | | 0.0006 |
|  |  |  |  |  |  |  |  |  | GO:0006821 | | | | | | | | | chloride transport | | | | | | | | | | | | | | | | | | | | | | | | | | | 14 | | | | | | | | | | 3 | | | | | | | | | | | 0.18 | | | | | | | 0.0007 | | | | | | | | | | | | | 0.0064 |
|  |  |  |  |  |  |  |  |  | GO:0009960 | | | | | | | | | endosperm development | | | | | | | | | | | | | | | | | | | | | | | | | | | 16 | | | | | | | | | | 3 | | | | | | | | | | | 0.21 | | | | | | | 0.0011 | | | | | | | | | | | | | 0.0064 |
|  |  |  |  |  |  |  |  |  | GO:2000762 | | | | | | | | | regulation of phenylpropanoid metabolic process | | | | | | | | | | | | | | | | | | | | | | | | | | | 16 | | | | | | | | | | 3 | | | | | | | | | | | 0.21 | | | | | | | 0.0011 | | | | | | | | | | | | | 0.0064 |
|  |  |  |  |  |  |  |  |  | GO:0098661 | | | | | | | | | inorganic anion transmembrane transport | | | | | | | | | | | | | | | | | | | | | | | | | | | 20 | | | | | | | | | | 3 | | | | | | | | | | | 0.26 | | | | | | | 0.0021 | | | | | | | | | | | | | 0.0085 |
|  |  |  |  |  |  |  |  |  | GO:0045490 | | | | | | | | | pectin catabolic process | | | | | | | | | | | | | | | | | | | | | | | | | | | 43 | | | | | | | | | | 4 | | | | | | | | | | | 0.56 | | | | | | | 0.0023 | | | | | | | | | | | | | 0.0085 |
|  |  |  |  |  |  |  |  |  | GO:0043455 | | | | | | | | | regulation of secondary metaboli process | | | | | | | | | | | | | | | | | | | | | | | | | | | 44 | | | | | | | | | | 4 | | | | | | | | | | | 0.57 | | | | | | | 0.0025 | | | | | | | | | | | | | 0.0085 |
|  |  |  |  |  |  |  |  |  | GO:2000026 | | | | | | | | | regulation of multicellular organismal development | | | | | | | | | | | | | | | | | | | | | | | | | | | 507 | | | | | | | | | | 15 | | | | | | | | | | | 6.57 | | | | | | | 0.0026 | | | | | | | | | | | | | 0.0085 |
|  |  |  |  |  |  |  |  |  | GO:0006556 | | | | | | | | | S-adenosylmethionine biosynthetic process | | | | | | | | | | | | | | | | | | | | | | | | | | | 7 | | | | | | | | | | 2 | | | | | | | | | | | 0.09 | | | | | | | 0.0034 | | | | | | | | | | | | | 0.0100 |
|  |  |  | MF | | | | | | GO:0005247 | | | | | | | | | voltage-gated chloride channel activity | | | | | | | | | | | | | | | | | | | | | | | | | | | 8 | | | | | | | | | | 3 | | | | | | | | | | | 0.12 | | | | | | | 0.00017 | | | | | | | | | | | | | 0.0014 |
|  |  |  |  |  |  |  |  |  | GO:0005254 | | | | | | | | | chloride channel activity | | | | | | | | | | | | | | | | | | | | | | | | | | | 8 | | | | | | | | | | 3 | | | | | | | | | | | 0.12 | | | | | | | 0.00017 | | | | | | | | | | | | | 0.0014 |
|  |  |  |  |  |  |  |  |  | GO:0038023 | | | | | | | | | signaling receptor activity | | | | | | | | | | | | | | | | | | | | | | | | | | | 235 | | | | | | | | | | 12 | | | | | | | | | | | 3.44 | | | | | | | 0.00019 | | | | | | | | | | | | | 0.0014 |
|  |  |  |  |  |  |  |  |  | GO:0005223 | | | | | | | | | intracellular cGMP-activated cation channel activity | | | | | | | | | | | | | | | | | | | | | | | | | | | 2 | | | | | | | | | | 2 | | | | | | | | | | | 0.03 | | | | | | | 0.00021 | | | | | | | | | | | | | 0.0014 |
|  |  |  |  |  |  |  |  |  | GO:0015108 | | | | | | | | | chloride transmembrane transporter activity | | | | | | | | | | | | | | | | | | | | | | | | | | | 9 | | | | | | | | | | 3 | | | | | | | | | | | 0.13 | | | | | | | 0.00024 | | | | | | | | | | | | | 0.0014 |
|  |  |  |  |  |  |  |  |  | GO:0004888 | | | | | | | | | transmembrane signaling receptor activity | | | | | | | | | | | | | | | | | | | | | | | | | | | 180 | | | | | | | | | | 10 | | | | | | | | | | | 2.64 | | | | | | | 0.00033 | | | | | | | | | | | | | 0.0016 |
|  |  |  |  |  |  |  |  |  | GO:0030599 | | | | | | | | | pectinesterase activity | | | | | | | | | | | | | | | | | | | | | | | | | | | 27 | | | | | | | | | | 4 | | | | | | | | | | | 0.4 | | | | | | | 0.00061 | | | | | | | | | | | | | 0.0022 |
|  |  |  |  |  |  |  |  |  | GO:0045330 | | | | | | | | | aspartyl esterase activity | | | | | | | | | | | | | | | | | | | | | | | | | | | 27 | | | | | | | | | | 4 | | | | | | | | | | | 0.4 | | | | | | | 0.00061 | | | | | | | | | | | | | 0.0022 |
|  |  |  |  |  |  |  |  |  | GO:0060089 | | | | | | | | | molecular transducer activity | | | | | | | | | | | | | | | | | | | | | | | | | | | 278 | | | | | | | | | | 12 | | | | | | | | | | | 4.07 | | | | | | | 0.00086 | | | | | | | | | | | | | 0.0028 |
|  |  |  |  |  |  |  |  |  | GO:0005217 | | | | | | | | | intracellular ligand-gated ion channel activity | | | | | | | | | | | | | | | | | | | | | | | | | | | 4 | | | | | | | | | | 2 | | | | | | | | | | | 0.06 | | | | | | | 0.00126 | | | | | | | | | | | | | 0.0037 |
|  |  |  | CC | | | | | | GO:0034707 | | | | | | | | | chloride channel complex | | | | | | | | | | | | | | | | | | | | | | | | | | | 7 | | | | | | | | | | 3 | | | | | | | | | | | 0.1 | | | | | | | 9.40E-05 | | | | | | | | | | | | | 0.0021 |
|  |  |  |  |  |  |  |  |  | GO:0034702 | | | | | | | | | ion channel complex | | | | | | | | | | | | | | | | | | | | | | | | | | | 8 | | | | | | | | | | 3 | | | | | | | | | | | 0.11 | | | | | | | 0.00015 | | | | | | | | | | | | | 0.0021 |
|  |  |  |  |  |  |  |  |  | GO:1902495 | | | | | | | | | transmembrane transporter complex | | | | | | | | | | | | | | | | | | | | | | | | | | | 9 | | | | | | | | | | 3 | | | | | | | | | | | 0.13 | | | | | | | 0.00022 | | | | | | | | | | | | | 0.0021 |
|  |  |  |  |  |  |  |  |  | GO:1990351 | | | | | | | | | transporter complex | | | | | | | | | | | | | | | | | | | | | | | | | | | 15 | | | | | | | | | | 3 | | | | | | | | | | | 0.21 | | | | | | | 0.00112 | | | | | | | | | | | | | 0.0082 |
|  |  |  |  |  |  |  |  |  | GO:0009705 | | | | | | | | | plant-type vacuole membrane | | | | | | | | | | | | | | | | | | | | | | | | | | | 118 | | | | | | | | | | 7 | | | | | | | | | | | 1.67 | | | | | | | 0.00145 | | | | | | | | | | | | | 0.0085 |
|  |  |  |  |  |  |  |  |  | GO:0000325 | | | | | | | | | plant-type vacuole | | | | | | | | | | | | | | | | | | | | | | | | | | | 155 | | | | | | | | | | 7 | | | | | | | | | | | 2.19 | | | | | | | 0.00658 | | | | | | | | | | | | | 0.0321 |
|  |  |  | Down-regulated | | | | | | | | | | | | | | | | | | | | | | | | | | | | | | | | | | | | | | | | | | | | | | | | | | | | | | | | | | | | | | | | | | | | | | | | | | | | | | | | | | | |
|  |  |  | BP | | | | | | GO:1902288 | | | | | | | | | regulation of defense response to oomycetes | | | | | | | | | | | | | | | | | | | | | | | | | | | 16 | | | | | | | | | | 3 | | | | | | | | | | | 0.07 | | | | | | | 4.90E-05 | | | | | | | | | | | | | 0.0014 |
|  |  |  |  |  |  |  |  |  | GO:0002230 | | | | | | | | | positive regulation of defense response to virus by host | | | | | | | | | | | | | | | | | | | | | | | | | | | 7 | | | | | | | | | | 2 | | | | | | | | | | | 0.03 | | | | | | | 0.00043 | | | | | | | | | | | | | 0.0042 |
|  |  |  |  |  |  |  |  |  | GO:0050691 | | | | | | | | | regulation of defense response to virus by host | | | | | | | | | | | | | | | | | | | | | | | | | | | 7 | | | | | | | | | | 2 | | | | | | | | | | | 0.03 | | | | | | | 0.00043 | | | | | | | | | | | | | 0.0042 |
|  |  |  |  |  |  |  |  |  | GO:0060866 | | | | | | | | | leaf abscission | | | | | | | | | | | | | | | | | | | | | | | | | | | 11 | | | | | | | | | | 2 | | | | | | | | | | | 0.05 | | | | | | | 0.00111 | | | | | | | | | | | | | 0.0068 |
|  |  |  |  |  |  |  |  |  | GO:1902290 | | | | | | | | | positive regulation of defense response to oomycetes | | | | | | | | | | | | | | | | | | | | | | | | | | | 12 | | | | | | | | | | 2 | | | | | | | | | | | 0.05 | | | | | | | 0.00132 | | | | | | | | | | | | | 0.0068 |
|  |  |  |  |  |  |  |  |  | GO:1900424 | | | | | | | | | regulation of defense response to bacteria | | | | | | | | | | | | | | | | | | | | | | | | | | | 53 | | | | | | | | | | 3 | | | | | | | | | | | 0.24 | | | | | | | 0.00183 | | | | | | | | | | | | | 0.0068 |
|  |  |  |  |  |  |  |  |  | GO:0009695 | | | | | | | | | jasmonic acid biosynthetic process | | | | | | | | | | | | | | | | | | | | | | | | | | | 15 | | | | | | | | | | 2 | | | | | | | | | | | 0.07 | | | | | | | 0.00209 | | | | | | | | | | | | | 0.0068 |
|  |  |  |  |  |  |  |  |  | GO:1900057 | | | | | | | | | positive regulation of leaf senescence | | | | | | | | | | | | | | | | | | | | | | | | | | | 15 | | | | | | | | | | 2 | | | | | | | | | | | 0.07 | | | | | | | 0.00209 | | | | | | | | | | | | | 0.0068 |
|  |  |  |  |  |  |  |  |  | GO:1905623 | | | | | | | | | positive regulation of leaf development | | | | | | | | | | | | | | | | | | | | | | | | | | | 15 | | | | | | | | | | 2 | | | | | | | | | | | 0.07 | | | | | | | 0.00209 | | | | | | | | | | | | | 0.0068 |
| KEGG | | | **Pathway code** | | | | | | | | | **Pathway name** | | | | | **p.value** | | | | | | | | | | **Annotated** | | | | | | **Tair Gene Symbol** | | | | | | **KEGG Gene** | | | | | | | | | | | | **RefSeq** | | | | | | | | | | ***Lactuca sativa* Gene** | | | | | | | | **Transcript** | | | | | | **FC** | | | | | | | | | | | **p-value** |
|  |  |  | ath04141 | | | | | | | | | Protein processing in endoplasmic reticulum | | | | | 0.054 | | | | | | | | | | 1 | | | | | | AT4G34100 | | | | | | E3 ubiquitin-protein ligase MARCH6 [EC:2.3.2.27] | | | | | | | | | | | | CER9; RING/U-box superfamily prot | | | | | | | | | | Probable E3 ubiquitin ligase SUD1 | | | | | | | | TCONS_00015185 | | | | | | 0.91 | | | | | | | | | | | 1.02E-08 |
|  |  |  | ath04626 | | | | | | | | | Plant-pathogen interaction | | | | | 0.073 | | | | | | | | | | 1 | | | | | | AT5G57940 | | | | | | cyclic nucleotide gated channel, plant | | | | | | | | | | | | CNGC5; cyclic nucleotide gated channel 5 | | | | | | | | | | Probable cyclic nucleotide-gated ion channel 5 | | | | | | | | TCONS_00023370 | | | | | | 0.91 | | | | | | | | | | | 9.40E-08 |
|  |  |  | ath02010 | | | | | | | | | ABC transporters | | | | | 0.083 | | | | | | | | | | 1 | | | | | | AT1G02520 | | | | | | ATP-binding cassette, subfamily B | | | | | | | | | | | | ABCB11; P-glycoprotein 11 | | | | | | | | | | ABC transporter B family member 11 | | | | | | | | TCONS_00025896 | | | | | | 0.65 | | | | | | | | | | | 7.11E-06 |
|  |  |  | ath00130 | | | | | | | | | Ubiquinone and other terpenoid-quinone biosynthesis | | | | | 0.088 | | | | | | | | | | 1 | | | | | | AT4G36750 | | | | | | NAD(P)H dehydrogenase (quinone) [EC:1.6.5.2] | | | | | | | | | | | | Quinone reductase family protein | | | | | | | | | | Probable NAD(P)H dehydrogenase (quinone) FQR1-like 2 | | | | | | | | TCONS_00015809 | | | | | | 2.06 | | | | | | | | | | | 2.30E-05 |
| **L-KIO_3_ vs L-Ctrl** | | | | | | | | | | | | | | | | | | | | | | | | | | | | | | | | | | | | | | | | | | | | | | | | | | | | | | | | | | | | | | | | | | | | | | | | | | | | | | | | | | | | | | |
| Up-regulated | | | | | | | | | | | | | | | | | | | | | | | | | | | | | | | | | | | | | | | | | | | | | | | | | | | | | | | | | | | | | | | | | | | | | | | | | | | | | | | | | | | | | | |
| topGO | | |  | | | | GO.ID | | | | | | | | | | | | Term | | | | | | | | | | | | | | | | | | | | | | | | | | | | | | Annotated | | | | | | | Significant | | | | | | Expected | | | | | | | | | | classicFisher | | | | | q_value | | | | | | | | | |
|  |  |  | BP | | | | GO:0048511 | | | | | | | | | | | | rhythmic process | | | | | | | | | | | | | | | | | | | | | | | | | | | | | | 140 | | | | | | | 2 | | | | | | 0.03 | | | | | | | | | | 0.00033 | | | | | 0.0097 | | | | | | | | | |
|  |  |  |  |  |  |  | GO:0010304 | | | | | | | | | | | | PSII associated light-harvesting complex II catabolic process | | | | | | | | | | | | | | | | | | | | | | | | | | | | | | 5 | | | | | | | 1 | | | | | | 0 | | | | | | | | | | 0.00108 | | | | | 0.0158 | | | | | | | | | |
|  |  |  |  |  |  |  | GO:0015996 | | | | | | | | | | | | chlorophyll catabolic process | | | | | | | | | | | | | | | | | | | | | | | | | | | | | | 19 | | | | | | | 1 | | | | | | 0 | | | | | | | | | | 0.00408 | | | | | 0.0181 | | | | | | | | | |
|  |  |  |  |  |  |  | GO:0042761 | | | | | | | | | | | | very long-chain fatty acid biosynthetic process | | | | | | | | | | | | | | | | | | | | | | | | | | | | | | 19 | | | | | | | 1 | | | | | | 0 | | | | | | | | | | 0.00408 | | | | | 0.0181 | | | | | | | | | |
|  |  |  |  |  |  |  | GO:0046149 | | | | | | | | | | | | pigment catabolic process | | | | | | | | | | | | | | | | | | | | | | | | | | | | | | 19 | | | | | | | 1 | | | | | | 0 | | | | | | | | | | 0.00408 | | | | | 0.0181 | | | | | | | | | |
|  |  |  |  |  |  |  | GO:0006787 | | | | | | | | | | | | porphyrin-containing compound catabolic process | | | | | | | | | | | | | | | | | | | | | | | | | | | | | | 22 | | | | | | | 1 | | | | | | 0 | | | | | | | | | | 0.00472 | | | | | 0.0180 | | | | | | | | | |
|  |  |  |  |  |  |  | GO:0033015 | | | | | | | | | | | | tetrapyrrole catabolic process | | | | | | | | | | | | | | | | | | | | | | | | | | | | | | 22 | | | | | | | 1 | | | | | | 0 | | | | | | | | | | 0.00472 | | | | | 0.018086 | | | | | | | | | |
|  |  |  |  |  |  |  | GO:0000038 | | | | | | | | | | | | very long-chain fatty acid metabolic process | | | | | | | | | | | | | | | | | | | | | | | | | | | | | | 23 | | | | | | | 1 | | | | | | 0 | | | | | | | | | | 0.00494 | | | | | 0.018086 | | | | | | | | | |
|  |  |  |  |  |  |  | GO:0009911 | | | | | | | | | | | | positive regulation of flower development | | | | | | | | | | | | | | | | | | | | | | | | | | | | | | 31 | | | | | | | 1 | | | | | | 0.01 | | | | | | | | | | 0.00665 | | | | | 0.021642 | | | | | | | | | |
|  |  |  |  |  |  |  | GO:0010025 | | | | | | | | | | | | wax biosynthetic process | | | | | | | | | | | | | | | | | | | | | | | | | | | | | | 38 | | | | | | | 1 | | | | | | 0.01 | | | | | | | | | | 0.00815 | | | | | 0.023871 | | | | | | | | | |
|  |  |  | MF | | | | GO:0034256 | | | | | | | | | | | | chlorophyll(ide) b reductase activity | | | | | | | | | | | | | | | | | | | | | | | | | | | | | | 3 | | | | | | | 1 | | | | | | 0 | | | | | | | | | | 0.00078 | | | | | 0.022846 | | | | | | | | | |
|  |  |  | Down-regulated | | | | | | | | | | | | | | | | | | | | | | | | | | | | | | | | | | | | | | | | | | | | | | | | | | | | | | | | | | | | | | | | | | | | | | | | | | | | | | | | | | | |
|  |  |  | BP | | | | GO:0042754 | | | | | | | | | | | | negative regulation of circadian rhythm | | | | | | | | | | | | | | | | | | | | | | | | | | | | | | 5 | | | | | | | 1 | | | | | | 0 | | | | | | | | | | 0.0019 | | | | | 0.033683 | | | | | | | | | |
|  |  |  |  |  |  |  | GO:0043433 | | | | | | | | | | | | negative regulation of DNA-binding transcription factor activity | | | | | | | | | | | | | | | | | | | | | | | | | | | | | | 6 | | | | | | | 1 | | | | | | 0 | | | | | | | | | | 0.0023 | | | | | 0.033683 | | | | | | | | | |
|  | | | **Pathway code** | | | | | | | | **Pathway name** | | | | | | | | | | | | | **p.value** | | | | | | **Annotated** | | | | | | **Tair Gene Symbol** | | | | | | **KEGG Gene** | | | | | | **RefSeq** | | | | | | | | | | | | | | ***Lactuca sativa* Gene** | | | | | | | **Transcript** | | | | | | **FC** | | | | | | | **p-value** | | | | |
| KEGG | | | ath04712 | | | | | | | | Circadian rhythm – plant | | | | | | | | | | | | | 0.6 | | | | | | 3 | | | | | | AT1G01060 | | | | | | MYB-related transcription factor LHY | | | | | | LHY; Homeodomain-like superfamily protein | | | | | | | | | | | | | | Protein LHY | | | | | | | TCONS_00008700 | | | | | | -2.03 | | | | | | | 0.01416 | | | | |
|  |  |  |  |  |  |  |  |  |  |  |  |  |  |  |  |  |  |  |  |  |  |  |  |  |  |  |  |  |  |  |  |  |  |  |  | AT1G68050 | | | | | | flavin-binding kelch repeat F-box protein 1 | | | | | | FKF1; flavin-binding, kelch repeat, f box 1 | | | | | | | | | | | | | | Adagio protein 3 | | | | | | | TCONS_00000142 | | | | | | 4.73 | | | | | | | 0.00224 | | | | |
|  |  |  |  |  |  |  |  |  |  |  |  |  |  |  |  |  |  |  |  |  |  |  |  |  |  |  |  |  |  |  |  |  |  |  |  | AT5G24470 | | | | | | pseudo-response regulator 5 | | | | | | PRR5; two-component response regulator-like protein | | | | | | | | | | | | | | Two-component response regulator-like APRR5 | | | | | | | TCONS_00022710 | | | | | | 5.14 | | | | | | | 3.96E-05 | | | | |
|  |  |  | ath00860 | | | | | | | | Porphyrin and chlorophyll metabolism | | | | | | | | | | | | | 0.1 | | | | | | 1 | | | | | | AT4G13250 | | | | | | chlorophyll(ide) b reductase [EC:1.1.1.294] | | | | | | NYC1; NAD(P)-binding Rossmann-fold superfamily protein | | | | | | | | | | | | | | Probable chlorophyll(ide) b reductase NYC1 | | | | | | | TCONS_00015014 | | | | | | 3.05 | | | | | | | 0.01628 | | | | |
| topGO | | | **R-SA vs R-Ctrl** | | | | | | | | | | | | | | | | | | | | | | | | | | | | | | | | | | | | | | | | | | | | | | | | | | | | | | | | | | | | | | | | | | | | | | | | | | | | | | | | | | | |
|  |  |  | Up-regulated | | | | | | | | | | | | | | | | | | | | | | | | | | | | | | | | | | | | | | | | | | | | | | | | | | | | | | | | | | | | | | | | | | | | | | | | | | | | | | | | | | | |
|  |  |  |  | | | GO.ID | | | | | | | | | | | Term | | | | | | | | | | | | | | | | | | | | | | | | | | | | | | | | | Annotated | | | | | | Significant | | | | | | | | | | | Expected | | | | | | classicFisher | | | | | | | | | | | q_value | | |
|  |  |  | BP | | | GO:0051028 | | | | | | | | | | | mRNA transport | | | | | | | | | | | | | | | | | | | | | | | | | | | | | | | | | 94 | | | | | | 6 | | | | | | | | | | | 0.97 | | | | | | 0.00043 | | | | | | | | | | | 0.00527 | | |
|  |  |  |  |  |  | GO:2000762 | | | | | | | | | | | regulation of phenylpropanoid metabolic process | | | | | | | | | | | | | | | | | | | | | | | | | | | | | | | | | 16 | | | | | | 3 | | | | | | | | | | | 0.17 | | | | | | 0.00055 | | | | | | | | | | | 0.00527 | | |
|  |  |  |  |  |  | GO:0050657 | | | | | | | | | | | nucleic acid transport | | | | | | | | | | | | | | | | | | | | | | | | | | | | | | | | | 111 | | | | | | 6 | | | | | | | | | | | 1.15 | | | | | | 0.00103 | | | | | | | | | | | 0.00527 | | |
|  |  |  |  |  |  | GO:0050658 | | | | | | | | | | | RNA transport | | | | | | | | | | | | | | | | | | | | | | | | | | | | | | | | | 111 | | | | | | 6 | | | | | | | | | | | 1.15 | | | | | | 0.00103 | | | | | | | | | | | 0.00527 | | |
|  |  |  |  |  |  | GO:0043455 | | | | | | | | | | | regulation of secondary metabolic process | | | | | | | | | | | | | | | | | | | | | | | | | | | | | | | | | 44 | | | | | | 4 | | | | | | | | | | | 0.45 | | | | | | 0.00108 | | | | | | | | | | | 0.00527 | | |
|  |  |  |  |  |  | GO:0051236 | | | | | | | | | | | establishment of RNA localization | | | | | | | | | | | | | | | | | | | | | | | | | | | | | | | | | 112 | | | | | | 6 | | | | | | | | | | | 1.16 | | | | | | 0.00108 | | | | | | | | | | | 0.00527 | | |
|  |  |  |  |  |  | GO:0006403 | | | | | | | | | | | RNA localization | | | | | | | | | | | | | | | | | | | | | | | | | | | | | | | | | 120 | | | | | | 6 | | | | | | | | | | | 1.24 | | | | | | 0.00155 | | | | | | | | | | | 0.00586 | | |
|  |  |  |  |  |  | GO:0016071 | | | | | | | | | | | mRNA metabolic process | | | | | | | | | | | | | | | | | | | | | | | | | | | | | | | | | 545 | | | | | | 14 | | | | | | | | | | | 5.63 | | | | | | 0.00162 | | | | | | | | | | | 0.00586 | | |
|  |  |  |  |  |  | GO:0008380 | | | | | | | | | | | RNA splicing | | | | | | | | | | | | | | | | | | | | | | | | | | | | | | | | | 323 | | | | | | 10 | | | | | | | | | | | 3.33 | | | | | | 0.00199 | | | | | | | | | | | 0.00586 | | |
|  |  |  |  |  |  | GO:0071705 | | | | | | | | | | | nitrogen compound transport | | | | | | | | | | | | | | | | | | | | | | | | | | | | | | | | | 1089 | | | | | | 22 | | | | | | | | | | | 11.24 | | | | | | 0.002 | | | | | | | | | | | 0.00586 | | |
|  |  |  | MF | | | GO:0003723 | | | | | | | | | | | RNA binding | | | | | | | | | | | | | | | | | | | | | | | | | | | | | | | | | 1272 | | | | | | 30 | | | | | | | | | | | 12.45 | | | | | | 6.30E-06 | | | | | | | | | | | 0.00018 | | |
|  |  |  |  |  |  | GO:0003729 | | | | | | | | | | | mRNA binding | | | | | | | | | | | | | | | | | | | | | | | | | | | | | | | | | 321 | | | | | | 12 | | | | | | | | | | | 3.14 | | | | | | 8.00E-05 | | | | | | | | | | | 0.00093 | | |
|  |  |  |  |  |  | GO:0005223 | | | | | | | | | | | intracellular cGMP-activated cation channel activity | | | | | | | | | | | | | | | | | | | | | | | | | | | | | | | | | 2 | | | | | | 2 | | | | | | | | | | | 0.02 | | | | | | 9.50E-05 | | | | | | | | | | | 0.00093 | | |
|  |  |  |  |  |  | GO:0005217 | | | | | | | | | | | intracellular ligand-gated ion channel activity | | | | | | | | | | | | | | | | | | | | | | | | | | | | | | | | | 4 | | | | | | 2 | | | | | | | | | | | 0.04 | | | | | | 0.00056 | | | | | | | | | | | 0.00273 | | |
|  |  |  |  |  |  | GO:0005221 | | | | | | | | | | | intracellular cyclic nucleotide activated cation channel activity | | | | | | | | | | | | | | | | | | | | | | | | | | | | | | | | | 4 | | | | | | 2 | | | | | | | | | | | 0.04 | | | | | | 0.00056 | | | | | | | | | | | 0.00273 | | |
|  |  |  |  |  |  | GO:0043855 | | | | | | | | | | | cyclic nucleotide-gated ion channel activity | | | | | | | | | | | | | | | | | | | | | | | | | | | | | | | | | 4 | | | | | | 2 | | | | | | | | | | | 0.04 | | | | | | 0.00056 | | | | | | | | | | | 0.00273 | | |
|  |  |  |  |  |  | GO:0099094 | | | | | | | | | | | ligand-gated cation channel activity | | | | | | | | | | | | | | | | | | | | | | | | | | | | | | | | | 5 | | | | | | 2 | | | | | | | | | | | 0.05 | | | | | | 0.00093 | | | | | | | | | | | 0.00381 | | |
|  |  |  |  |  |  | GO:0003676 | | | | | | | | | | | nucleic acid binding | | | | | | | | | | | | | | | | | | | | | | | | | | | | | | | | | 3175 | | | | | | 48 | | | | | | | | | | | 31.07 | | | | | | 0.00104 | | | | | | | | | | | 0.00381 | | |
|  |  |  |  |  |  | GO:0051082 | | | | | | | | | | | unfolded protein binding | | | | | | | | | | | | | | | | | | | | | | | | | | | | | | | | | 139 | | | | | | 6 | | | | | | | | | | | 1.36 | | | | | | 0.00249 | | | | | | | | | | | 0.00753 | | |
|  |  |  |  |  |  | GO:0004165 | | | | | | | | | | | delta(3)-delta(2)-enoyl-CoA isomerase activity | | | | | | | | | | | | | | | | | | | | | | | | | | | | | | | | | 8 | | | | | | 2 | | | | | | | | | | | 0.08 | | | | | | 0.00257 | | | | | | | | | | | 0.00753 | | |
|  |  |  | CC | | | GO:0005634 | | | | | | | | | | | nucleus | | | | | | | | | | | | | | | | | | | | | | | | | | | | | | | | | 4746 | | | | | | 73 | | | | | | | | | | | 49.55 | | | | | | 0.00014 | | | | | | | | | | | 0.00410 | | |
|  |  |  |  |  |  | GO:0071014 | | | | | | | | | | | post-mRNA release spliceosomal complex | | | | | | | | | | | | | | | | | | | | | | | | | | | | | | | | | 6 | | | | | | 2 | | | | | | | | | | | 0.06 | | | | | | 0.00158 | | | | | | | | | | | 0.02314 | | |
|  |  |  | Down-regulated | | | | | | | | | | | | | | | | | | | | | | | | | | | | | | | | | | | | | | | | | | | | | | | | | | | | | | | | | | | | | | | | | | | | | | | | | | | | | | | | | | | |
|  |  |  | BP | | | GO:0031347 | | | | | | | | | | | regulation of defense response | | | | | | | | | | | | | | | | | | | | | | | | | | | | | | | | | 281 | | | | | | 9 | | | | | | | | | | | 1.1 | | | | | | 1.50E-06 | | | | | | | | | | | 0.00004 | | |
|  |  |  |  |  |  | GO:0031349 | | | | | | | | | | | positive regulation of defense response | | | | | | | | | | | | | | | | | | | | | | | | | | | | | | | | | 119 | | | | | | 6 | | | | | | | | | | | 0.47 | | | | | | 7.30E-06 | | | | | | | | | | | 0.00010 | | |
|  |  |  |  |  |  | GO:0045088 | | | | | | | | | | | regulation of innate immune response | | | | | | | | | | | | | | | | | | | | | | | | | | | | | | | | | 128 | | | | | | 6 | | | | | | | | | | | 0.5 | | | | | | 1.10E-05 | | | | | | | | | | | 0.00010 | | |
|  |  |  |  |  |  | GO:0002682 | | | | | | | | | | | regulation of immune system process | | | | | | | | | | | | | | | | | | | | | | | | | | | | | | | | | 199 | | | | | | 7 | | | | | | | | | | | 0.78 | | | | | | 1.30E-05 | | | | | | | | | | | 0.00010 | | |
|  |  |  |  |  |  | GO:1900055 | | | | | | | | | | | regulation of leaf senescence | | | | | | | | | | | | | | | | | | | | | | | | | | | | | | | | | 46 | | | | | | 4 | | | | | | | | | | | 0.18 | | | | | | 3.10E-05 | | | | | | | | | | | 0.00018 | | |
|  |  |  |  |  |  | GO:0080134 | | | | | | | | | | | regulation of response to stress | | | | | | | | | | | | | | | | | | | | | | | | | | | | | | | | | 419 | | | | | | 9 | | | | | | | | | | | 1.64 | | | | | | 3.70E-05 | | | | | | | | | | | 0.00018 | | |
|  |  |  |  |  |  | GO:0009611 | | | | | | | | | | | response to wounding | | | | | | | | | | | | | | | | | | | | | | | | | | | | | | | | | 174 | | | | | | 6 | | | | | | | | | | | 0.68 | | | | | | 6.20E-05 | | | | | | | | | | | 0.00025 | | |
|  |  |  |  |  |  | GO:0050776 | | | | | | | | | | | regulation of immune response | | | | | | | | | | | | | | | | | | | | | | | | | | | | | | | | | 177 | | | | | | 6 | | | | | | | | | | | 0.69 | | | | | | 6.90E-05 | | | | | | | | | | | 0.00025 | | |
|  |  |  |  |  |  | GO:2000031 | | | | | | | | | | | regulation of salicylic acid mediated si… | | | | | | | | | | | | | | | | | | | | | | | | | | | | | | | | | 23 | | | | | | 3 | | | | | | | | | | | 0.09 | | | | | | 9.70E-05 | | | | | | | | | | | 0.00032 | | |
|  |  |  | MF | | | GO:0140110 | | | | | | | | | | | transcription regulator activity | | | | | | | | | | | | | | | | | | | | | | | | | | | | | | | | | 1132 | | | | | | 13 | | | | | | | | | | | 4.32 | | | | | | 0.00032 | | | | | | | | | | | 0.00937 | | |
|  |  |  |  |  |  | GO:0003677 | | | | | | | | | | | DNA binding | | | | | | | | | | | | | | | | | | | | | | | | | | | | | | | | | 1928 | | | | | | 17 | | | | | | | | | | | 7.37 | | | | | | 0.00078 | | | | | | | | | | | 0.01142 | | |
|  |  |  |  |  |  | GO:0043565 | | | | | | | | | | | sequence-specific DNA binding | | | | | | | | | | | | | | | | | | | | | | | | | | | | | | | | | 722 | | | | | | 9 | | | | | | | | | | | 2.76 | | | | | | 0.00167 | | | | | | | | | | | 0.01257 | | |
|  |  |  |  |  |  | GO:0052731 | | | | | | | | | | | phosphocholine phosphatase activity | | | | | | | | | | | | | | | | | | | | | | | | | | | | | | | | | 1 | | | | | | 1 | | | | | | | | | | | 0 | | | | | | 0.00382 | | | | | | | | | | | 0.01257 | | |
|  |  |  |  |  |  | GO:0052732 | | | | | | | | | | | phosphoethanolamine phosphatase activity | | | | | | | | | | | | | | | | | | | | | | | | | | | | | | | | | 1 | | | | | | 1 | | | | | | | | | | | 0 | | | | | | 0.00382 | | | | | | | | | | | 0.01257 | | |
|  |  |  |  |  |  | GO:0061522 | | | | | | | | | | | 1,4-dihydroxy-2-naphthoyl-CoA thioestera… | | | | | | | | | | | | | | | | | | | | | | | | | | | | | | | | | 1 | | | | | | 1 | | | | | | | | | | | 0 | | | | | | 0.00382 | | | | | | | | | | | 0.01257 | | |
|  |  |  |  |  |  | GO:0070888 | | | | | | | | | | | E-box binding | | | | | | | | | | | | | | | | | | | | | | | | | | | | | | | | | 1 | | | | | | 1 | | | | | | | | | | | 0 | | | | | | 0.00382 | | | | | | | | | | | 0.01257 | | |
|  |  |  |  |  |  | GO:1990136 | | | | | | | | | | | linoleate 9S-lipoxygenase activity | | | | | | | | | | | | | | | | | | | | | | | | | | | | | | | | | 1 | | | | | | 1 | | | | | | | | | | | 0 | | | | | | 0.00382 | | | | | | | | | | | 0.01257 | | |
|  |  |  |  |  |  | GO:0046872 | | | | | | | | | | | metal ion binding | | | | | | | | | | | | | | | | | | | | | | | | | | | | | | | | | 3464 | | | | | | 23 | | | | | | | | | | | 13.23 | | | | | | 0.00398 | | | | | | | | | | | 0.01257 | | |
|  |  |  |  |  |  | GO:0043169 | | | | | | | | | | | cation binding | | | | | | | | | | | | | | | | | | | | | | | | | | | | | | | | | 3484 | | | | | | 23 | | | | | | | | | | | 13.31 | | | | | | 0.00429 | | | | | | | | | | | 0.01257 | | |
|  |  |  | CC | | | GO:0000815 | | | | | | | | | | | ESCRT III complex | | | | | | | | | | | | | | | | | | | | | | | | | | | | | | | | | 16 | | | | | | 2 | | | | | | | | | | | 0.06 | | | | | | 0.0015 | | | | | | | | | | | 0.03954 | | |
|  |  |  |  |  |  | GO:0042564 | | | | | | | | | | | NLS-dependent protein nuclear import com… | | | | | | | | | | | | | | | | | | | | | | | | | | | | | | | | | 1 | | | | | | 1 | | | | | | | | | | | 0 | | | | | | 0.0036 | | | | | | | | | | | 0.03954 | | |
|  |  |  |  |  |  | GO:0036452 | | | | | | | | | | | ESCRT complex | | | | | | | | | | | | | | | | | | | | | | | | | | | | | | | | | 30 | | | | | | 2 | | | | | | | | | | | 0.11 | | | | | | 0.0052 | | | | | | | | | | | 0.03954 | | |
|  |  |  |  |  |  | GO:0005634 | | | | | | | | | | | nucleus | | | | | | | | | | | | | | | | | | | | | | | | | | | | | | | | | 4746 | | | | | | 27 | | | | | | | | | | | 17.01 | | | | | | 0.0054 | | | | | | | | | | | 0.03954 | | |
|  |  |  |  |  |  | GO:0031074 | | | | | | | | | | | nucleocytoplasmic transport complex | | | | | | | | | | | | | | | | | | | | | | | | | | | | | | | | | 2 | | | | | | 1 | | | | | | | | | | | 0.01 | | | | | | 0.0072 | | | | | | | | | | | 0.04218 | | |
| KEGG | | | **Pathway code** | | | | | | | **Pathway name** | | | | **p.value** | | | | | | | | | | | | **Annotated** | | | | | | **Tair Gene Symbol** | | | | | | **KEGG Gene** | | | | | | | | | | | **RefSeq** | | | | | | | | | | ***Lactuca sativa* Gene** | | | | | | | | | | **Transcript** | | | | | | | **FC** | | | | | | | **p-value** | | | |
|  |  |  | ath00591 | | | | | | | Linoleic acid metabolism | | | | 0.077669 | | | | | | | | | | | | 1 | | | | | | AT3G22400 | | | | | | linoleate 9S-lipoxygenase | | | | | | | | | | | LOX5; PLAT/LH2 domain-containing lipoxyg | | | | | | | | | | Linoleate 9S-lipoxygenase 5 | | | | | | | | | | TCONS_00013610 | | | | | | | -0.56 | | | | | | | 7.64E-07 | | | |
|  |  |  | ath00280 | | | | | | | Valine, leucine and isoleucine degradation | | | | 0.101026 | | | | | | | | | | | | 1 | | | | | | AT2G33150 | | | | | | acetyl-CoA acyltransferase 1 | | | | | | | | | | | PKT3; peroxisomal 3-ketoacyl-CoA thiola | | | | | | | | | | 3-ketoacyl-CoA thiolase 2, peroxisomal | | | | | | | | | | TCONS_00013997 | | | | | | | -0.72 | | | | | | | 4.99E-06 | | | |
|  |  |  | ath00592 | | | | | | | alpha-Linolenic acid metabolism | | | | 0.101026 | | | | | | | | | | | | 1 | | | | | | AT2G33150 | | | | | | acetyl-CoA acyltransferase 1 | | | | | | | | | | | PKT3; peroxisomal 3-ketoacyl-CoA thiola | | | | | | | | | | 3-ketoacyl-CoA thiolase 2, peroxisomal | | | | | | | | | | TCONS_00013997 | | | | | | | -0.72 | | | | | | | 4.99E-06 | | | |
|  |  |  | ath01040 | | | | | | | Biosynthesis of unsaturated fatty acids | | | | 0.101026 | | | | | | | | | | | | 1 | | | | | | AT2G33150 | | | | | | acetyl-CoA acyltransferase 1 | | | | | | | | | | | PKT3; peroxisomal 3-ketoacyl-CoA thiola | | | | | | | | | | 3-ketoacyl-CoA thiolase 2, peroxisomal | | | | | | | | | | TCONS_00013997 | | | | | | | -0.72 | | | | | | | 4.99E-06 | | | |
|  |  |  | ath04146 | | | | | | | Peroxisome | | | | 0.11242 | | | | | | | | | | | | 2 | | | | | | AT2G24580 | | | | | | sarcosine oxidase / L-pipecolate oxidase | | | | | | | | | | | FAD-dependent oxidor | | | | | | | | | | Probable sarcosine oxidase | | | | | | | | | | TCONS_00024420 | | | | | | | 1.36 | | | | | | | 0.000884 | | | |
|  |  |  |  |  |  |  |  |  |  |  |  |  |  |  |  |  |  |  |  |  |  |  |  |  |  |  |  |  |  |  |  | AT2G33150 | | | | | | acetyl-CoA acyltransferase 1 | | | | | | | | | | | PKT3; peroxisomal 3-ketoacyl-CoA thiola | | | | | | | | | | 3-ketoacyl-CoA thiolase 2, peroxisomal | | | | | | | | | | TCONS_00013997 | | | | | | | -0.72 | | | | | | | 4.99E-06 | | | |
|  |  |  | ath00230 | | | | | | | Purine metabolism | | | | 0.256743 | | | | | | | | | | | | 1 | | | | | | AT1G09830 | | | | | | phosphoribosylamine---glycine ligase | | | | | | | | | | | PUR2; Glycinamide ribonucleotid | | | | | | | | | | Phosphoribosylamine–glycine ligase | | | | | | | | | | TCONS_00020424 | | | | | | | 1.32 | | | | | | | 0.000343 | | | |
| topGO | | | **L-Ctrl vs R-Ctrl** | | | | | | | | | | | | | | | | | | | | | | | | | | | | | | | | | | | | | | | | | | | | | | | | | | | | | | | | | | | | | | | | | | | | | | | | | | | | | | | | | | | |
|  |  |  | Up-regulated | | | | | | | | | | | | | | | | | | | | | | | | | | | | | | | | | | | | | | | | | | | | | | | | | | | | | | | | | | | | | | | | | | | | | | | | | | | | | | | | | | | |
|  |  |  |  | GO.ID | | | | | | | | | | | | Term | | | | | | | | | | | | | | | | | | | | | | | | | | | | Annotated | | | | | | | | | | Significant | | | | | | | | | | | Expected | | | | | | | classicFisher | | | | | | | | q_value | | | | | | |
|  |  |  | BP | GO:0009408 | | | | | | | | | | | | response to heat | | | | | | | | | | | | | | | | | | | | | | | | | | | | 214 | | | | | | | | | | 27 | | | | | | | | | | | 6.43 | | | | | | | 3.20E-10 | | | | | | | | 9.4E-09 | | | | | | |
|  |  |  |  | GO:0006457 | | | | | | | | | | | | protein folding | | | | | | | | | | | | | | | | | | | | | | | | | | | | 212 | | | | | | | | | | 26 | | | | | | | | | | | 6.37 | | | | | | | 1.30E-09 | | | | | | | | 1.9E-08 | | | | | | |
|  |  |  |  | GO:0009658 | | | | | | | | | | | | chloroplast organization | | | | | | | | | | | | | | | | | | | | | | | | | | | | 194 | | | | | | | | | | 20 | | | | | | | | | | | 5.83 | | | | | | | 1.70E-06 | | | | | | | | 1.5E-05 | | | | | | |
|  |  |  |  | GO:0042542 | | | | | | | | | | | | response to hydrogen peroxide | | | | | | | | | | | | | | | | | | | | | | | | | | | | 62 | | | | | | | | | | 11 | | | | | | | | | | | 1.86 | | | | | | | 2.10E-06 | | | | | | | | 1.5E-05 | | | | | | |
|  |  |  |  | GO:0009266 | | | | | | | | | | | | response to temperature stimulus | | | | | | | | | | | | | | | | | | | | | | | | | | | | 484 | | | | | | | | | | 34 | | | | | | | | | | | 14.55 | | | | | | | 4.40E-06 | | | | | | | | 2.6E-05 | | | | | | |
|  |  |  |  | GO:0009657 | | | | | | | | | | | | plastid organization | | | | | | | | | | | | | | | | | | | | | | | | | | | | 233 | | | | | | | | | | 21 | | | | | | | | | | | 7 | | | | | | | 8.00E-06 | | | | | | | | 3.9E-05 | | | | | | |
|  |  |  |  | GO:0045036 | | | | | | | | | | | | protein targeting to chloroplast | | | | | | | | | | | | | | | | | | | | | | | | | | | | 28 | | | | | | | | | | 7 | | | | | | | | | | | 0.84 | | | | | | | 1.50E-05 | | | | | | | | 5.2E-05 | | | | | | |
|  |  |  |  | GO:0072596 | | | | | | | | | | | | establishment of protein localization to chloroplast | | | | | | | | | | | | | | | | | | | | | | | | | | | | 28 | | | | | | | | | | 7 | | | | | | | | | | | 0.84 | | | | | | | 1.50E-05 | | | | | | | | 5.2E-05 | | | | | | |
|  |  |  |  | GO:0045037 | | | | | | | | | | | | protein import into chloroplast stroma | | | | | | | | | | | | | | | | | | | | | | | | | | | | 12 | | | | | | | | | | 5 | | | | | | | | | | | 0.36 | | | | | | | 1.60E-05 | | | | | | | | 5.2E-05 | | | | | | |
|  |  |  |  | GO:0072598 | | | | | | | | | | | | protein localization to chloroplast | | | | | | | | | | | | | | | | | | | | | | | | | | | | 30 | | | | | | | | | | 7 | | | | | | | | | | | 0.9 | | | | | | | 2.40E-05 | | | | | | | | 7.0E-05 | | | | | | |
|  |  |  | MF | GO:0051082 | | | | | | | | | | | | unfolded protein binding | | | | | | | | | | | | | | | | | | | | | | | | | | | | 139 | | | | | | | | | | 21 | | | | | | | | | | | 4.15 | | | | | | | 9.80E-10 | | | | | | | | 2.9E-08 | | | | | | |
|  |  |  |  | GO:0032553 | | | | | | | | | | | | ribonucleotide binding | | | | | | | | | | | | | | | | | | | | | | | | | | | | 2798 | | | | | | | | | | 127 | | | | | | | | | | | 83.47 | | | | | | | 4.90E-07 | | | | | | | | 7.2E-06 | | | | | | |
|  |  |  |  | GO:0097367 | | | | | | | | | | | | carbohydrate derivative binding | | | | | | | | | | | | | | | | | | | | | | | | | | | | 2850 | | | | | | | | | | 127 | | | | | | | | | | | 85.02 | | | | | | | 1.30E-06 | | | | | | | | 1.3E-05 | | | | | | |
|  |  |  |  | GO:0017111 | | | | | | | | | | | | nucleoside-triphosphatase activity | | | | | | | | | | | | | | | | | | | | | | | | | | | | 784 | | | | | | | | | | 48 | | | | | | | | | | | 23.39 | | | | | | | 2.10E-06 | | | | | | | | 1.5E-05 | | | | | | |
|  |  |  |  | GO:0035639 | | | | | | | | | | | | purine ribonucleoside triphosphate binding | | | | | | | | | | | | | | | | | | | | | | | | | | | | 2734 | | | | | | | | | | 121 | | | | | | | | | | | 81.56 | | | | | | | 3.60E-06 | | | | | | | | 1.8E-05 | | | | | | |
|  |  |  |  | GO:0032555 | | | | | | | | | | | | purine ribonucleotide binding | | | | | | | | | | | | | | | | | | | | | | | | | | | | 2768 | | | | | | | | | | 122 | | | | | | | | | | | 82.57 | | | | | | | 3.90E-06 | | | | | | | | 1.8E-05 | | | | | | |
|  |  |  |  | GO:0017076 | | | | | | | | | | | | purine nucleotide binding | | | | | | | | | | | | | | | | | | | | | | | | | | | | 2772 | | | | | | | | | | 122 | | | | | | | | | | | 82.69 | | | | | | | 4.20E-06 | | | | | | | | 1.8E-05 | | | | | | |
|  |  |  |  | GO:0016817 | | | | | | | | | | | | hydrolase activity, acting on acid anhydrides | | | | | | | | | | | | | | | | | | | | | | | | | | | | 861 | | | | | | | | | | 50 | | | | | | | | | | | 25.68 | | | | | | | 5.50E-06 | | | | | | | | 2.0E-05 | | | | | | |
|  |  |  |  | GO:0005200 | | | | | | | | | | | | structural constituent of cytoskeleton | | | | | | | | | | | | | | | | | | | | | | | | | | | | 45 | | | | | | | | | | 9 | | | | | | | | | | | 1.34 | | | | | | | 6.00E-06 | | | | | | | | 2.0E-05 | | | | | | |
|  |  |  |  | GO:0016462 | | | | | | | | | | | | pyrophosphatase activity | | | | | | | | | | | | | | | | | | | | | | | | | | | | 849 | | | | | | | | | | 49 | | | | | | | | | | | 25.33 | | | | | | | 8.10E-06 | | | | | | | | 2.4E-05 | | | | | | |
|  |  |  | CC | GO:0044444 | | | | | | | | | | | | cytoplasmic part | | | | | | | | | | | | | | | | | | | | | | | | | | | | 7442 | | | | | | | | | | 287 | | | | | | | | | | | 227.67 | | | | | | | 2.90E-07 | | | | | | | | 5.7E-06 | | | | | | |
|  |  |  |  | GO:0009507 | | | | | | | | | | | | chloroplast | | | | | | | | | | | | | | | | | | | | | | | | | | | | 2298 | | | | | | | | | | 111 | | | | | | | | | | | 70.3 | | | | | | | 5.60E-07 | | | | | | | | 5.7E-06 | | | | | | |
|  |  |  |  | GO:0005737 | | | | | | | | | | | | cytoplasm | | | | | | | | | | | | | | | | | | | | | | | | | | | | 8989 | | | | | | | | | | 333 | | | | | | | | | | | 275 | | | | | | | 7.20E-07 | | | | | | | | 5.7E-06 | | | | | | |
|  |  |  |  | GO:0009536 | | | | | | | | | | | | plastid | | | | | | | | | | | | | | | | | | | | | | | | | | | | 2340 | | | | | | | | | | 112 | | | | | | | | | | | 71.59 | | | | | | | 7.80E-07 | | | | | | | | 5.7E-06 | | | | | | |
|  |  |  |  | GO:0044434 | | | | | | | | | | | | chloroplast part | | | | | | | | | | | | | | | | | | | | | | | | | | | | 1317 | | | | | | | | | | 70 | | | | | | | | | | | 40.29 | | | | | | | 4.20E-06 | | | | | | | | 2.5E-05 | | | | | | |
|  |  |  |  | GO:0044435 | | | | | | | | | | | | plastid part | | | | | | | | | | | | | | | | | | | | | | | | | | | | 1333 | | | | | | | | | | 70 | | | | | | | | | | | 40.78 | | | | | | | 6.30E-06 | | | | | | | | 3.1E-05 | | | | | | |
|  |  |  |  | GO:0009570 | | | | | | | | | | | | chloroplast stroma | | | | | | | | | | | | | | | | | | | | | | | | | | | | 555 | | | | | | | | | | 37 | | | | | | | | | | | 16.98 | | | | | | | 8.50E-06 | | | | | | | | 3.5E-05 | | | | | | |
|  |  |  |  | GO:0009941 | | | | | | | | | | | | chloroplast envelope | | | | | | | | | | | | | | | | | | | | | | | | | | | | 536 | | | | | | | | | | 36 | | | | | | | | | | | 16.4 | | | | | | | 9.50E-06 | | | | | | | | 3.5E-05 | | | | | | |
|  |  |  |  | GO:0009526 | | | | | | | | | | | | plastid envelope | | | | | | | | | | | | | | | | | | | | | | | | | | | | 545 | | | | | | | | | | 36 | | | | | | | | | | | 16.67 | | | | | | | 1.40E-05 | | | | | | | | 4.4E-05 | | | | | | |
|  |  |  |  | GO:0009532 | | | | | | | | | | | | plastid stroma | | | | | | | | | | | | | | | | | | | | | | | | | | | | 569 | | | | | | | | | | 37 | | | | | | | | | | | 17.41 | | | | | | | 1.50E-05 | | | | | | | | 4.4E-05 | | | | | | |
|  |  |  | Down-regulated | | | | | | | | | | | | | | | | | | | | | | | | | | | | | | | | | | | | | | | | | | | | | | | | | | | | | | | | | | | | | | | | | | | | | | | | | | | | | | | | | | | |
|  |  |  | BP | GO:0010200 | | | | | | | | | | | | response to chitin | | | | | | | | | | | | | | | | | | | | | | | | | | | | 120 | | | | | | | | | | 16 | | | | | | | | | | | 2.62 | | | | | | | 7.80E-09 | | | | | | | | 2.28E-07 | | | | | | |
|  |  |  |  | GO:0006952 | | | | | | | | | | | | defense response | | | | | | | | | | | | | | | | | | | | | | | | | | | | 1451 | | | | | | | | | | 61 | | | | | | | | | | | 31.68 | | | | | | | 5.40E-07 | | | | | | | | 7.91E-06 | | | | | | |
|  |  |  |  | GO:0010243 | | | | | | | | | | | | response to organonitrogen compound | | | | | | | | | | | | | | | | | | | | | | | | | | | | 256 | | | | | | | | | | 19 | | | | | | | | | | | 5.59 | | | | | | | 3.80E-06 | | | | | | | | 3.71E-05 | | | | | | |
|  |  |  |  | GO:1901698 | | | | | | | | | | | | response to nitrogen compound | | | | | | | | | | | | | | | | | | | | | | | | | | | | 319 | | | | | | | | | | 20 | | | | | | | | | | | 6.96 | | | | | | | 2.60E-05 | | | | | | | | 0.000164 | | | | | | |
|  |  |  |  | GO:0042493 | | | | | | | | | | | | response to drug | | | | | | | | | | | | | | | | | | | | | | | | | | | | 546 | | | | | | | | | | 28 | | | | | | | | | | | 11.92 | | | | | | | 2.80E-05 | | | | | | | | 0.000164 | | | | | | |
|  |  |  |  | GO:0009873 | | | | | | | | | | | | ethylene-activated signaling pathway | | | | | | | | | | | | | | | | | | | | | | | | | | | | 203 | | | | | | | | | | 15 | | | | | | | | | | | 4.43 | | | | | | | 4.20E-05 | | | | | | | | 0.00018 | | | | | | |
|  |  |  |  | GO:0048583 | | | | | | | | | | | | regulation of response to stimulus | | | | | | | | | | | | | | | | | | | | | | | | | | | | 873 | | | | | | | | | | 38 | | | | | | | | | | | 19.06 | | | | | | | 4.30E-05 | | | | | | | | 0.00018 | | | | | | |
|  |  |  |  | GO:0071369 | | | | | | | | | | | | cellular response to ethylene stimulus | | | | | | | | | | | | | | | | | | | | | | | | | | | | 213 | | | | | | | | | | 15 | | | | | | | | | | | 4.65 | | | | | | | 7.20E-05 | | | | | | | | 0.000214 | | | | | | |
|  |  |  |  | GO:0006950 | | | | | | | | | | | | response to stress | | | | | | | | | | | | | | | | | | | | | | | | | | | | 3231 | | | | | | | | | | 101 | | | | | | | | | | | 70.54 | | | | | | | 7.30E-05 | | | | | | | | 0.000214 | | | | | | |
|  |  |  | MF | GO:0043565 | | | | | | | | | | | | sequence-specific DNA binding | | | | | | | | | | | | | | | | | | | | | | | | | | | | 722 | | | | | | | | | | 36 | | | | | | | | | | | 15.72 | | | | | | | 3.60E-06 | | | | | | | | 0.000105 | | | | | | |
|  |  |  |  | GO:0140110 | | | | | | | | | | | | transcription regulator activity | | | | | | | | | | | | | | | | | | | | | | | | | | | | 1132 | | | | | | | | | | 44 | | | | | | | | | | | 24.64 | | | | | | | 0.00014 | | | | | | | | 0.00205 | | | | | | |
|  |  |  |  | GO:0003700 | | | | | | | | | | | | DNA-binding transcription factor activity | | | | | | | | | | | | | | | | | | | | | | | | | | | | 1022 | | | | | | | | | | 40 | | | | | | | | | | | 22.25 | | | | | | | 0.00025 | | | | | | | | 0.002441 | | | | | | |
|  |  |  |  | GO:0008083 | | | | | | | | | | | | growth factor activity | | | | | | | | | | | | | | | | | | | | | | | | | | | | 2 | | | | | | | | | | 2 | | | | | | | | | | | 0.04 | | | | | | | 0.00047 | | | | | | | | 0.003222 | | | | | | |
|  |  |  |  | GO:0051213 | | | | | | | | | | | | dioxygenase activity | | | | | | | | | | | | | | | | | | | | | | | | | | | | 180 | | | | | | | | | | 12 | | | | | | | | | | | 3.92 | | | | | | | 0.0006 | | | | | | | | 0.003222 | | | | | | |
|  |  |  |  | GO:0008061 | | | | | | | | | | | | chitin binding | | | | | | | | | | | | | | | | | | | | | | | | | | | | 19 | | | | | | | | | | 4 | | | | | | | | | | | 0.41 | | | | | | | 0.00066 | | | | | | | | 0.003222 | | | | | | |
|  |  |  |  | GO:0051019 | | | | | | | | | | | | mitogen-activated protein kinase binding | | | | | | | | | | | | | | | | | | | | | | | | | | | | 3 | | | | | | | | | | 2 | | | | | | | | | | | 0.07 | | | | | | | 0.0014 | | | | | | | | 0.005455 | | | | | | |
|  |  |  |  | GO:0016702 | | | | | | | | | | | | oxidoreductase activity, acting on singl… | | | | | | | | | | | | | | | | | | | | | | | | | | | | 39 | | | | | | | | | | 5 | | | | | | | | | | | 0.85 | | | | | | | 0.00149 | | | | | | | | 0.005455 | | | | | | |
|  |  |  |  | GO:0004842 | | | | | | | | | | | | ubiquitin-protein transferase activity | | | | | | | | | | | | | | | | | | | | | | | | | | | | 441 | | | | | | | | | | 20 | | | | | | | | | | | 9.6 | | | | | | | 0.00171 | | | | | | | | 0.005565 | | | | | | |
|  |  |  |  | GO:0019787 | | | | | | | | | | | | ubiquitin-like protein transferase activity | | | | | | | | | | | | | | | | | | | | | | | | | | | | 455 | | | | | | | | | | 20 | | | | | | | | | | | 9.91 | | | | | | | 0.00246 | | | | | | | | 0.007205 | | | | | | |
|  | | |  |  | | | | | | | | | | | |  | | | | | | | | | | | | | | | | | | | | | | | | | | | |  | | | | | | | | | |  | | | | | | | | | | |  | | | | | | |  | | | | | | | |  | | | | | | |
| KEGG | | **Pathway code** | | | | | | | | **Pathway name** | | | | | | | | | | | | | **p.value** | | | | | | **Annotated** | | | | | | **Tair Gene Symbol** | | | | | | **KEGG Gene** | | | | | | **RefSeq** | | | | | | | | | | | ***Lactuca sativa* Gene** | | | | | | | | | | | **Transcript** | | | | | | | **FC** | | | | | | | | | **p-value** | |
|  |  | ath00010 | | | | | | | | Glycolysis / Gluconeogenesis | | | | | | | | | | | | | 0.038744 | | | | | | 4 | | | | | | AT5G54960 | | | | | | pyruvate decarboxylase [EC:4.1.1.1] | | | | | | PDC2; pyruvate decarboxylase-2 | | | | | | | | | | | Pyruvate decarboxylase 2 | | | | | | | | | | | TCONS_00003639 | | | | | | | -2.02 | | | | | | | | | 0.005218 | |
|  |  |  |  |  |  |  |  |  |  |  |  |  |  |  |  |  |  |  |  |  |  |  |  |  |  |  |  |  |  |  |  |  |  |  | AT1G32780 | | | | | | alcohol dehydrogenase [EC:1.1.1.1] | | | | | | GroES-like zinc-binding dehydrogenase family protein | | | | | | | | | | | Alcohol dehydrogenase-like 3 | | | | | | | | | | | TCONS_00006274 | | | | | | | -2.23 | | | | | | | | | 0.002265 | |
|  |  |  |  |  |  |  |  |  |  |  |  |  |  |  |  |  |  |  |  |  |  |  |  |  |  |  |  |  |  |  |  |  |  |  | AT3G50520 | | | | | | probable phosphoglycerate mutase [EC:5.4.2.12] | | | | | | Phosphoglycerate mutase family protein | | | | | | | | | | | Phosphoglycerate mutase-like protein 4 | | | | | | | | | | | TCONS_00012029 | | | | | | | 1.67 | | | | | | | | | 0.000107 | |
|  |  |  |  |  |  |  |  |  |  |  |  |  |  |  |  |  |  |  |  |  |  |  |  |  |  |  |  |  |  |  |  |  |  |  | AT5G52920 | | | | | | pyruvate kinase [EC:2.7.1.40] | | | | | | PKP-BETA1; plastidic pyruvate kinase beta subunit 1 | | | | | | | | | | | Plastidial pyruvate kinase 2 | | | | | | | | | | | TCONS_00004619 | | | | | | | 0.77 | | | | | | | | | 0.020428 | |
|  |  | ath00592 | | | | | | | | alpha-Linolenic acid metabolism | | | | | | | | | | | | | 0.046529 | | | | | | 2 | | | | | | AT1G67560 | | | | | | lipoxygenase [EC:1.13.11.12] | | | | | | LOX6; PLAT/LH2 domain-containing lipoxygenase family | | | | | | | | | | | Lipoxygenase 6, chloroplastic | | | | | | | | | | | TCONS_00007754 | | | | | | | -1.99 | | | | | | | | | 0.002177 | |
|  |  |  |  |  |  |  |  |  |  |  |  |  |  |  |  |  |  |  |  |  |  |  |  |  |  |  |  |  |  |  |  |  |  |  | AT2G33150 | | | | | | acetyl-CoA acyltransferase 1 [EC:2.3.1.16] | | | | | | PKT3; peroxisomal 3-ketoacyl-CoA thiolase 3 | | | | | | | | | | | 3-ketoacyl-CoA thiolase 2, peroxisomal | | | | | | | | | | | TCONS_00013997 | | | | | | | -1.46 | | | | | | | | | 0.002177 | |
|  |  | ath00071 | | | | | | | | Fatty acid degradation | | | | | | | | | | | | | 0.048276 | | | | | | 2 | | | | | | AT1G32780 | | | | | | alcohol dehydrogenase [EC:1.1.1.1] | | | | | | GroES-like zinc-binding dehydrogenase family protein | | | | | | | | | | | Alcohol dehydrogenase-like 3 | | | | | | | | | | | TCONS_00006274 | | | | | | | -2.23 | | | | | | | | | 0.002265 | |
|  |  |  |  |  |  |  |  |  |  |  |  |  |  |  |  |  |  |  |  |  |  |  |  |  |  |  |  |  |  |  |  |  |  |  | AT2G33150 | | | | | | acetyl-CoA acyltransferase 1 [EC:2.3.1.16] | | | | | | PKT3; peroxisomal 3-ketoacyl-CoA thiolase 3 | | | | | | | | | | | 3-ketoacyl-CoA thiolase 2, peroxisoma | | | | | | | | | | | TCONS_00013997 | | | | | | | -1.46 | | | | | | | | | 0.002177 | |
|  |  | ath04146 | | | | | | | | Peroxisome | | | | | | | | | | | | | 0.060354 | | | | | | 2 | | | | | | AT2G24580 | | | | | | sarcosine oxidase / L-pipecolate oxidase [EC:1.5.3.1 1.5.3.7] | | | | | | FAD-dependent oxidoreductase family protein | | | | | | | | | | | Probable sarcosine oxidase | | | | | | | | | | | TCONS_00024420 | | | | | | | 1.61 | | | | | | | | | 0.00379 | |
|  |  |  |  |  |  |  |  |  |  |  |  |  |  |  |  |  |  |  |  |  |  |  |  |  |  |  |  |  |  |  |  |  |  |  | AT2G33150 | | | | | | acetyl-CoA acyltransferase 1 [EC:2.3.1.16] | | | | | | PKT3; peroxisomal 3-ketoacyl-CoA thiolase 3 | | | | | | | | | | | 3-ketoacyl-CoA thiolase 2, peroxisomal | | | | | | | | | | | TCONS_00013997 | | | | | | | -1.46 | | | | | | | | | 0.002177 | |
|  |  | ath00591 | | | | | | | | Linoleic acid metabolism | | | | | | | | | | | | | 0.062741 | | | | | | 2 | | | | | | AT1G67560 | | | | | | lipoxygenase [EC:1.13.11.12] | | | | | | LOX6; PLAT/LH2 domain-containing lipoxygenase family | | | | | | | | | | | Lipoxygenase 6, chloroplastic | | | | | | | | | | | TCONS_00007754 | | | | | | | -1.99 | | | | | | | | | 0.002177 | |
|  |  |  |  |  |  |  |  |  |  |  |  |  |  |  |  |  |  |  |  |  |  |  |  |  |  |  |  |  |  |  |  |  |  |  | AT3G22400 | | | | | | linoleate 9S-lipoxygenase [EC:1.13.11.58] | | | | | | PLAT/LH2 domain-containing lipoxygenase family protein | | | | | | | | | | | Linoleate 9S-lipoxygenase 5 | | | | | | | | | | | TCONS_00013610 | | | | | | | -0.40 | | | | | | | | | 0.003817 | |
|  |  | ath04712 | | | | | | | | Circadian rhythm – plant | | | | | | | | | | | | | 0.082589 | | | | | | 1 | | | | | | AT5G57360 | | | | | | clock-associated PAS protein ZTL | | | | | | ZTL; Galactose oxidase/kelch repeat superfamily protein | | | | | | | | | | | Adagio protein 1 | | | | | | | | | | | TCONS_00020250 | | | | | | | 1.63 | | | | | | | | | 0.000608 | |
| topGO | | **L-KIO_3_ vs R-KIO_3_** | | | | | | | | | | | | | | | | | | | | | | | | | | | | | | | | | | | | | | | | | | | | | | | | | | | | | | | | | | | | | | | | | | | | | | | | | | | | | | | | | | | | |
|  |  | Up-regulated | | | | | | | | | | | | | | | | | | | | | | | | | | | | | | | | | | | | | | | | | | | | | | | | | | | | | | | | | | | | | | | | | | | | | | | | | | | | | | | | | | | | |
|  | |  | | | | GO.ID | | | | | | | | | | | | | | | | Term | | | | | | | | | | | | | | | | | | | | | | | | | Annotated | | | | | | Significant | | | | | | | | | | | Expected | | | | | | | classicFisher | | | | | | | | q_value | | | | | | | |
|  |  | BP | | | | GO:0009658 | | | | | | | | | | | | | | | | chloroplast organization | | | | | | | | | | | | | | | | | | | | | | | | | 194 | | | | | | 114 | | | | | | | | | | | 44.11 | | | | | | | 2.20E-27 | | | | | | | | 5.6E-26 | | | | | | | |
|  |  |  |  |  |  | GO:0055114 | | | | | | | | | | | | | | | | oxidation-reduction process | | | | | | | | | | | | | | | | | | | | | | | | | 1335 | | | | | | 455 | | | | | | | | | | | 303.53 | | | | | | | 5.80E-23 | | | | | | | | 7.4E-22 | | | | | | | |
|  |  |  |  |  |  | GO:0015979 | | | | | | | | | | | | | | | | photosynthesis | | | | | | | | | | | | | | | | | | | | | | | | | 364 | | | | | | 155 | | | | | | | | | | | 82.76 | | | | | | | 1.60E-17 | | | | | | | | 1.4E-16 | | | | | | | |
|  |  |  |  |  |  | GO:0033013 | | | | | | | | | | | | | | | | tetrapyrrole metabolic process | | | | | | | | | | | | | | | | | | | | | | | | | 107 | | | | | | 61 | | | | | | | | | | | 24.33 | | | | | | | 1.80E-14 | | | | | | | | 1.1E-13 | | | | | | | |
|  |  |  |  |  |  | GO:0015994 | | | | | | | | | | | | | | | | chlorophyll metabolic process | | | | | | | | | | | | | | | | | | | | | | | | | 90 | | | | | | 54 | | | | | | | | | | | 20.46 | | | | | | | 3.10E-14 | | | | | | | | 1.6E-13 | | | | | | | |
|  |  |  |  |  |  | GO:0006778 | | | | | | | | | | | | | | | | porphyrin-containing compound metabolic ... | | | | | | | | | | | | | | | | | | | | | | | | | 104 | | | | | | 59 | | | | | | | | | | | 23.65 | | | | | | | 6.60E-14 | | | | | | | | 2.7E-13 | | | | | | | |
|  |  |  |  |  |  | GO:0042440 | | | | | | | | | | | | | | | | pigment metabolic process | | | | | | | | | | | | | | | | | | | | | | | | | 176 | | | | | | 85 | | | | | | | | | | | 40.02 | | | | | | | 7.40E-14 | | | | | | | | 2.7E-13 | | | | | | | |
|  |  |  |  |  |  | GO:0046148 | | | | | | | | | | | | | | | | pigment biosynthetic process | | | | | | | | | | | | | | | | | | | | | | | | | 146 | | | | | | 74 | | | | | | | | | | | 33.19 | | | | | | | 1.30E-13 | | | | | | | | 4.1E-13 | | | | | | | |
|  |  |  |  |  |  | GO:0051186 | | | | | | | | | | | | | | | | cofactor metabolic process | | | | | | | | | | | | | | | | | | | | | | | | | 595 | | | | | | 212 | | | | | | | | | | | 135.28 | | | | | | | 2.90E-13 | | | | | | | | 8.2E-13 | | | | | | | |
|  |  | MF | | | | GO:0003824 | | | | | | | | | | | | | | | | catalytic activity | | | | | | | | | | | | | | | | | | | | | | | | | 8099 | | | | | | 2170 | | | | | | | | | | | 1846.31 | | | | | | | 1.40E-29 | | | | | | | | 3.6E-28 | | | | | | | |
|  |  |  |  |  |  | GO:0048037 | | | | | | | | | | | | | | | | cofactor binding | | | | | | | | | | | | | | | | | | | | | | | | | 1167 | | | | | | 391 | | | | | | | | | | | 266.04 | | | | | | | 4.10E-18 | | | | | | | | 5.2E-17 | | | | | | | |
|  |  |  |  |  |  | GO:0016705 | | | | | | | | | | | | | | | | oxidoreductase activity, acting on paire... | | | | | | | | | | | | | | | | | | | | | | | | | 325 | | | | | | 138 | | | | | | | | | | | 74.09 | | | | | | | 1.60E-15 | | | | | | | | 1.4E-14 | | | | | | | |
|  |  |  |  |  |  | GO:0004497 | | | | | | | | | | | | | | | | monooxygenase activity | | | | | | | | | | | | | | | | | | | | | | | | | 286 | | | | | | 119 | | | | | | | | | | | 65.2 | | | | | | | 7.50E-13 | | | | | | | | 4.8E-12 | | | | | | | |
|  |  |  |  |  |  | GO:0005506 | | | | | | | | | | | | | | | | iron ion binding | | | | | | | | | | | | | | | | | | | | | | | | | 312 | | | | | | 117 | | | | | | | | | | | 71.13 | | | | | | | 2.60E-09 | | | | | | | | 1.3E-08 | | | | | | | |
|  |  |  |  |  |  | GO:0020037 | | | | | | | | | | | | | | | | heme binding | | | | | | | | | | | | | | | | | | | | | | | | | 358 | | | | | | 130 | | | | | | | | | | | 81.61 | | | | | | | 3.70E-09 | | | | | | | | 1.6E-08 | | | | | | | |
|  |  |  |  |  |  | GO:0003674 | | | | | | | | | | | | | | | | molecular_function | | | | | | | | | | | | | | | | | | | | | | | | | 14443 | | | | | | 3431 | | | | | | | | | | | 3292.53 | | | | | | | 1.10E-08 | | | | | | | | 4.0E-08 | | | | | | | |
|  |  |  |  |  |  | GO:0016709 | | | | | | | | | | | | | | | | oxidoreductase activity, acting on paire... | | | | | | | | | | | | | | | | | | | | | | | | | 124 | | | | | | 55 | | | | | | | | | | | 28.27 | | | | | | | 8.30E-08 | | | | | | | | 2.6E-07 | | | | | | | |
|  |  |  |  |  |  | GO:0046906 | | | | | | | | | | | | | | | | tetrapyrrole binding | | | | | | | | | | | | | | | | | | | | | | | | | 458 | | | | | | 153 | | | | | | | | | | | 104.41 | | | | | | | 1.00E-07 | | | | | | | | 2.8E-07 | | | | | | | |
|  |  | Down-regulated | | | | | | | | | | | | | | | | | | | | | | | | | | | | | | | | | | | | | | | | | | | | | | | | | | | | | | | | | | | | | | | | | | | | | | | | | | | | | | | | | | | | |
|  |  | BP | | | | GO:0009415 | | | | | | | | | | | | | | | | response to water | | | | | | | | | | | | | | | | | | | | | | | | | 368 | | | | | | 82 | | | | | | | | | | | 42.61 | | | | | | | 3.10E-09 | | | | | | | | 6.9E-08 | | | | | | | |
|  |  |  |  |  |  | GO:0009414 | | | | | | | | | | | | | | | | response to water deprivation | | | | | | | | | | | | | | | | | | | | | | | | | 365 | | | | | | 81 | | | | | | | | | | | 42.26 | | | | | | | 4.70E-09 | | | | | | | | 6.9E-08 | | | | | | | |
|  |  |  |  |  |  | GO:0006334 | | | | | | | | | | | | | | | | nucleosome assembly | | | | | | | | | | | | | | | | | | | | | | | | | 54 | | | | | | 23 | | | | | | | | | | | 6.25 | | | | | | | 7.80E-09 | | | | | | | | 7.6E-08 | | | | | | | |
|  |  |  |  |  |  | GO:0010035 | | | | | | | | | | | | | | | | response to inorganic substance | | | | | | | | | | | | | | | | | | | | | | | | | 777 | | | | | | 140 | | | | | | | | | | | 89.96 | | | | | | | 4.80E-08 | | | | | | | | 2.5E-07 | | | | | | | |
|  |  |  |  |  |  | GO:0031497 | | | | | | | | | | | | | | | | chromatin assembly | | | | | | | | | | | | | | | | | | | | | | | | | 63 | | | | | | 24 | | | | | | | | | | | 7.29 | | | | | | | 5.10E-08 | | | | | | | | 2.5E-07 | | | | | | | |
|  |  |  |  |  |  | GO:0006333 | | | | | | | | | | | | | | | | chromatin assembly or disassembly | | | | | | | | | | | | | | | | | | | | | | | | | 72 | | | | | | 26 | | | | | | | | | | | 8.34 | | | | | | | 5.10E-08 | | | | | | | | 2.5E-07 | | | | | | | |
|  |  |  |  |  |  | GO:0042221 | | | | | | | | | | | | | | | | response to chemical | | | | | | | | | | | | | | | | | | | | | | | | | 2583 | | | | | | 381 | | | | | | | | | | | 299.05 | | | | | | | 7.80E-08 | | | | | | | | 3.3E-07 | | | | | | | |
|  |  |  |  |  |  | GO:0019748 | | | | | | | | | | | | | | | | secondary metabolic process | | | | | | | | | | | | | | | | | | | | | | | | | 304 | | | | | | 66 | | | | | | | | | | | 35.2 | | | | | | | 2.90E-07 | | | | | | | | 1.1E-06 | | | | | | | |
|  |  |  |  |  |  | GO:0006323 | | | | | | | | | | | | | | | | DNA packaging | | | | | | | | | | | | | | | | | | | | | | | | | 75 | | | | | | 25 | | | | | | | | | | | 8.68 | | | | | | | 5.40E-07 | | | | | | | | 1.8E-06 | | | | | | | |
|  |  | MF | | | | GO:0046982 | | | | | | | | | | | | | | | | protein heterodimerization activity | | | | | | | | | | | | | | | | | | | | | | | | | 171 | | | | | | 73 | | | | | | | | | | | 19.8 | | | | | | | 4.50E-25 | | | | | | | | 1.3E-23 | | | | | | | |
|  |  |  |  |  |  | GO:0046983 | | | | | | | | | | | | | | | | protein dimerization activity | | | | | | | | | | | | | | | | | | | | | | | | | 561 | | | | | | 119 | | | | | | | | | | | 64.97 | | | | | | | 2.60E-11 | | | | | | | | 3.8E-10 | | | | | | | |
|  |  |  |  |  |  | GO:0016705 | | | | | | | | | | | | | | | | oxidoreductase activity, acting on paire... | | | | | | | | | | | | | | | | | | | | | | | | | 325 | | | | | | 70 | | | | | | | | | | | 37.64 | | | | | | | 1.80E-07 | | | | | | | | 1.8E-06 | | | | | | | |
|  |  |  |  |  |  | GO:0016491 | | | | | | | | | | | | | | | | oxidoreductase activity | | | | | | | | | | | | | | | | | | | | | | | | | 1710 | | | | | | 261 | | | | | | | | | | | 198.04 | | | | | | | 9.00E-07 | | | | | | | | 6.6E-06 | | | | | | | |
|  |  |  |  |  |  | GO:0003700 | | | | | | | | | | | | | | | | DNA-binding transcription factor activit... | | | | | | | | | | | | | | | | | | | | | | | | | 1022 | | | | | | 162 | | | | | | | | | | | 118.36 | | | | | | | 1.60E-05 | | | | | | | | 9.4E-05 | | | | | | | |
|  |  |  |  |  |  | GO:0020037 | | | | | | | | | | | | | | | | heme binding | | | | | | | | | | | | | | | | | | | | | | | | | 358 | | | | | | 68 | | | | | | | | | | | 41.46 | | | | | | | 2.50E-05 | | | | | | | | 1.2E-04 | | | | | | | |
|  |  |  |  |  |  | GO:0003677 | | | | | | | | | | | | | | | | DNA binding | | | | | | | | | | | | | | | | | | | | | | | | | 1928 | | | | | | 279 | | | | | | | | | | | 223.29 | | | | | | | 2.80E-05 | | | | | | | | 1.2E-04 | | | | | | | |
|  |  |  |  |  |  | GO:0016717 | | | | | | | | | | | | | | | | oxidoreductase activity, acting on paire... | | | | | | | | | | | | | | | | | | | | | | | | | 27 | | | | | | 11 | | | | | | | | | | | 3.13 | | | | | | | 0.00011 | | | | | | | | 4.0E-04 | | | | | | | |
|  |  |  |  |  |  | GO:0031492 | | | | | | | | | | | | | | | | nucleosomal DNA binding | | | | | | | | | | | | | | | | | | | | | | | | | 16 | | | | | | 8 | | | | | | | | | | | 1.85 | | | | | | | 0.00017 | | | | | | | | 5.5E-04 | | | | | | | |
|  |  |  |  |  |  | GO:0005544 | | | | | | | | | | | | | | | | calcium-dependent phospholipid binding | | | | | | | | | | | | | | | | | | | | | | | | | 20 | | | | | | 9 | | | | | | | | | | | 2.32 | | | | | | | 0.00019 | | | | | | | | 5.6E-04 | | | | | | | |
|  |  | CC | | | | GO:0000785 | | | | | | | | | | | | | | | | chromatin | | | | | | | | | | | | | | | | | | | | | | | | | 239 | | | | | | 80 | | | | | | | | | | | 25.99 | | | | | | | 4.40E-21 | | | | | | | | 8.0E-20 | | | | | | | |
|  |  |  |  |  |  | GO:0044427 | | | | | | | | | | | | | | | | chromosomal part | | | | | | | | | | | | | | | | | | | | | | | | | 370 | | | | | | 92 | | | | | | | | | | | 40.24 | | | | | | | 1.40E-14 | | | | | | | | 1.3E-13 | | | | | | | |
|  |  |  |  |  |  | GO:0071944 | | | | | | | | | | | | | | | | cell periphery | | | | | | | | | | | | | | | | | | | | | | | | | 3233 | | | | | | 460 | | | | | | | | | | | 351.63 | | | | | | | 4.70E-11 | | | | | | | | 2.8E-10 | | | | | | | |
|  |  |  |  |  |  | GO:0005694 | | | | | | | | | | | | | | | | chromosome | | | | | | | | | | | | | | | | | | | | | | | | | 428 | | | | | | 92 | | | | | | | | | | | 46.55 | | | | | | | 9.30E-11 | | | | | | | | 4.2E-10 | | | | | | | |
|  |  |  |  |  |  | GO:0005886 | | | | | | | | | | | | | | | | plasma membrane | | | | | | | | | | | | | | | | | | | | | | | | | 2771 | | | | | | 379 | | | | | | | | | | | 301.38 | | | | | | | 3.70E-07 | | | | | | | | 1.3E-06 | | | | | | | |
|  |  |  |  |  |  | GO:0005576 | | | | | | | | | | | | | | | | extracellular region | | | | | | | | | | | | | | | | | | | | | | | | | 928 | | | | | | 145 | | | | | | | | | | | 100.93 | | | | | | | 3.70E-06 | | | | | | | | 1.1E-05 | | | | | | | |
|  |  |  |  |  |  | GO:0005618 | | | | | | | | | | | | | | | | cell wall | | | | | | | | | | | | | | | | | | | | | | | | | 564 | | | | | | 95 | | | | | | | | | | | 61.34 | | | | | | | 9.30E-06 | | | | | | | | 2.4E-05 | | | | | | | |
| KEGG | **Pathway code** | | | | | | | **Pathway name** | | | | | | | **p.value** | | | | | | | | | | **Annotated** | | | | | | **Tair Gene Symbol** | | | | | | **KEGG Gene** | | | | | | **RefSeq** | | | | | | | | | | | | | | ***Lactuca sativa* Gene** | | | | | | | | | | | **Transcript** | | | | | | **FC** | | | | | | | **p-value** | | | | | |
|  | ath00970 | | | | | | | Aminoacyl-tRNA biosynthesis | | | | | | | 1.25E-06 | | | | | | | | | | 17 | | | | | | AT1G11870 | | | | | | seryl-tRNA synthetase [EC:6.1.1.11] | | | | | | SRS; Seryl-tRNA synthetase | | | | | | | | | | | | | | Serine--tRNA ligase, chloroplastic/mitochondrial | | | | | | | | | | | TCONS_00025295 | | | | | | 1.98 | | | | | | | 0.003478 | | | | | |
|  |  |  |  |  |  |  |  |  |  |  |  |  |  |  |  |  |  |  |  |  |  |  |  |  |  |  |  |  |  |  | AT1G29880 | | | | | | glycyl-tRNA synthetase [EC:6.1.1.14] | | | | | | glycyl-tRNA synthetase / glycine-tRNA ligase | | | | | | | | | | | | | | Glycine--tRNA ligase, mitochondrial 1 | | | | | | | | | | | TCONS_00022309 | | | | | | 0.78 | | | | | | | 0.007673 | | | | | |
|  |  |  |  |  |  |  |  |  |  |  |  |  |  |  |  |  |  |  |  |  |  |  |  |  |  |  |  |  |  |  | AT2G04842 | | | | | | threonyl-tRNA synthetase [EC:6.1.1.3] | | | | | | EMB2761;threonyl-tRNA synthetase, putative / threonine-tRNA ligase | | | | | | | | | | | | | | Threonine--tRNA ligase, chloroplastic/mitochondrial 2 | | | | | | | | | | | TCONS_00013307 | | | | | | 2.41 | | | | | | | 0.000356 | | | | | |
|  |  |  |  |  |  |  |  |  |  |  |  |  |  |  |  |  |  |  |  |  |  |  |  |  |  |  |  |  |  |  | AT2G25840 | | | | | | tryptophanyl-tRNA synthetase [EC:6.1.1.2] | | | | | | OVA4; Nucleotidylyl transferase superfamily protein | | | | | | | | | | | | | | Tryptophan--tRNA ligase, chloroplastic/mitochondrial | | | | | | | | | | | TCONS_00006424 | | | | | | 2.12 | | | | | | | 0.000235 | | | | | |
|  |  |  |  |  |  |  |  |  |  |  |  |  |  |  |  |  |  |  |  |  |  |  |  |  |  |  |  |  |  |  | AT2G31170 | | | | | | cysteinyl-tRNA synthetase [EC:6.1.1.16] | | | | | | SYCO_ARATH; Cysteinyl-tRNA synthetase, class Ia family protein | | | | | | | | | | | | | | Cysteine--tRNA ligase, chloroplastic/mitochondrial | | | | | | | | | | | TCONS_00017181 | | | | | | 1.89 | | | | | | | 0.002126 | | | | | |
|  |  |  |  |  |  |  |  |  |  |  |  |  |  |  |  |  |  |  |  |  |  |  |  |  |  |  |  |  |  |  | AT3G02660 | | | | | | tyrosyl-tRNA synthetase [EC:6.1.1.1] | | | | | | EMB2768; Tyrosyl-tRNA synthetase, class Ib, bacterial/mitochondrial | | | | | | | | | | | | | | Tyrosine--tRNA ligase, chloroplastic/mitochondrial | | | | | | | | | | | TCONS_00017386 | | | | | | 1.79 | | | | | | | 5.52E-08 | | | | | |
|  |  |  |  |  |  |  |  |  |  |  |  |  |  |  |  |  |  |  |  |  |  |  |  |  |  |  |  |  |  |  | AT3G13490 | | | | | | lysyl-tRNA synthetase, class II [EC:6.1.1.6] | | | | | | OVA5;Lysyl-tRNA synthetase, class II | | | | | | | | | | | | | | Lysine--tRNA ligase, chloroplastic/mitochondrial | | | | | | | | | | | TCONS_00017724 | | | | | | 1.32 | | | | | | | 0.000533 | | | | | |
|  |  |  |  |  |  |  |  |  |  |  |  |  |  |  |  |  |  |  |  |  |  |  |  |  |  |  |  |  |  |  | AT3G46100 | | | | | | histidyl-tRNA synthetase [EC:6.1.1.21] | | | | | | HRS1; Histidyl-tRNA synthetase 1 | | | | | | | | | | | | | | Histidine--tRNA ligase, chloroplastic/mitochondrial | | | | | | | | | | | TCONS_00005932 | | | | | | 2.20 | | | | | | | 3.88E-05 | | | | | |
|  |  |  |  |  |  |  |  |  |  |  |  |  |  |  |  |  |  |  |  |  |  |  |  |  |  |  |  |  |  |  | AT3G48110 | | | | | | glycyl-tRNA synthetase [EC:6.1.1.14] | | | | | | EDD1; glycine-tRNA ligase | | | | | | | | | | | | | | Glycine--tRNA ligase, chloroplastic/mitochondrial 2 | | | | | | | | | | | TCONS_00010898 | | | | | | 2.19 | | | | | | | 0.000209 | | | | | |
|  |  |  |  |  |  |  |  |  |  |  |  |  |  |  |  |  |  |  |  |  |  |  |  |  |  |  |  |  |  |  | AT3G55400 | | | | | | methionyl-tRNA synthetase [EC:6.1.1.10] | | | | | | OVA1; methionyl-tRNA synthetase / methionine-tRNA ligase / MetRS (cpMetRS) | | | | | | | | | | | | | | Methionine--tRNA ligase, chloroplastic/mitochondrial | | | | | | | | | | | TCONS_00025238 | | | | | | 2.57 | | | | | | | 0.001007 | | | | | |
|  |  |  |  |  |  |  |  |  |  |  |  |  |  |  |  |  |  |  |  |  |  |  |  |  |  |  |  |  |  |  | AT3G58140 | | | | | | phenylalanyl-tRNA synthetase alpha chain [EC:6.1.1.20] | | | | | | phenylalanyl-tRNA synthetase class IIc family protein | | | | | | | | | | | | | | Phenylalanine--tRNA ligase, chloroplastic/mitochondrial | | | | | | | | | | | TCONS_00023965 | | | | | | 1.23 | | | | | | | 0.002222 | | | | | |
|  |  |  |  |  |  |  |  |  |  |  |  |  |  |  |  |  |  |  |  |  |  |  |  |  |  |  |  |  |  |  | AT4G04350 | | | | | | leucyl-tRNA synthetase [EC:6.1.1.4] | | | | | | EMB2369;tRNA synthetase class I (I, L, M and V) family protein | | | | | | | | | | | | | | Leucine--tRNA ligase, chloroplastic/mitochondrial | | | | | | | | | | | TCONS_00011092 | | | | | | 3.35 | | | | | | | 0.000127 | | | | | |
|  |  |  |  |  |  |  |  |  |  |  |  |  |  |  |  |  |  |  |  |  |  |  |  |  |  |  |  |  |  |  | AT4G17300 | | | | | | asparaginyl-tRNA synthetase [EC:6.1.1.22] | | | | | | NS1; Class II aminoacyl-tRNA and biotin synthetases superfamily protein | | | | | | | | | | | | | | Asparagine--tRNA ligase, chloroplastic/mitochondrial | | | | | | | | | | | TCONS_00016931 | | | | | | 1.46 | | | | | | | 0.001701 | | | | | |
|  |  |  |  |  |  |  |  |  |  |  |  |  |  |  |  |  |  |  |  |  |  |  |  |  |  |  |  |  |  |  | AT4G33760 | | | | | | aspartyl-tRNA synthetase [EC:6.1.1.12] | | | | | | tRNA synthetase class II (D, K and N) family protein | | | | | | | | | | | | | | Aspartate--tRNA ligase, chloroplastic/mitochondrial | | | | | | | | | | | TCONS_00025680 | | | | | | 1.26 | | | | | | | 0.00068 | | | | | |
|  |  |  |  |  |  |  |  |  |  |  |  |  |  |  |  |  |  |  |  |  |  |  |  |  |  |  |  |  |  |  | AT5G16715 | | | | | | valyl-tRNA synthetase [EC:6.1.1.9] | | | | | | EMB2247; protein EMBRYO DEFECTIVE 2247 | | | | | | | | | | | | | | Valine--tRNA ligase, chloroplastic/mitochondrial 2 | | | | | | | | | | | TCONS_00006813 | | | | | | 3.16 | | | | | | | 7.95E-07 | | | | | |
|  |  |  |  |  |  |  |  |  |  |  |  |  |  |  |  |  |  |  |  |  |  |  |  |  |  |  |  |  |  |  | AT5G49030 | | | | | | isoleucyl-tRNA synthetase [EC:6.1.1.5] | | | | | | OVA2; tRNA synthetase class I (I, L, M and V) family protein | | | | | | | | | | | | | | Isoleucine--tRNA ligase, chloroplastic/mitochondrial | | | | | | | | | | | TCONS_00012730 | | | | | | 2.26 | | | | | | | 2.78E-05 | | | | | |
|  |  |  |  |  |  |  |  |  |  |  |  |  |  |  |  |  |  |  |  |  |  |  |  |  |  |  |  |  |  |  | AT5G52520 | | | | | | prolyl-tRNA synthetase [EC:6.1.1.15] | | | | | | OVA6;Class II aaRS and biotin synthetases superfamily protein | | | | | | | | | | | | | | Proline--tRNA ligase, chloroplastic/mitochondrial | | | | | | | | | | | TCONS_00006032 | | | | | | 1.87 | | | | | | | 0.001551 | | | | | |
|  | ath00906 | | | | | | | Carotenoid biosynthesis | | | | | | | 2.09E-03 | | | | | | | | | | 6 | | | | | | AT1G03055 | | | | | | beta-carotene isomerase [EC:5.2.1.14] | | | | | | D27; beta-carotene isomerase D27-like protein | | | | | | | | | | | | | | Beta-carotene isomerase D27, chloroplastic | | | | | | | | | | | TCONS_00024383 | | | | | | 2.32 | | | | | | | 0.003939 | | | | | |
|  |  |  |  |  |  |  |  |  |  |  |  |  |  |  |  |  |  |  |  |  |  |  |  |  |  |  |  |  |  |  | AT1G08550 | | | | | | violaxanthin de-epoxidase [EC:1.23.5.1] | | | | | | NPQ1; non-photochemical quenching 1 | | | | | | | | | | | | | | Violaxanthin de-epoxidase, chloroplastic | | | | | | | | | | | TCONS_00012675 | | | | | | 3.63 | | | | | | | 0.000907 | | | | | |
|  |  |  |  |  |  |  |  |  |  |  |  |  |  |  |  |  |  |  |  |  |  |  |  |  |  |  |  |  |  |  | AT1G10830 | | | | | | zeta-carotene isomerase [EC:5.2.1.12] | | | | | | Z-ISO; 15-cis-zeta-carotene isomerase | | | | | | | | | | | | | | 15-cis-zeta-carotene isomerase, chloroplastic | | | | | | | | | | | TCONS_00005031 | | | | | | 2.04 | | | | | | | 0.000326 | | | | | |
|  |  |  |  |  |  |  |  |  |  |  |  |  |  |  |  |  |  |  |  |  |  |  |  |  |  |  |  |  |  |  | AT1G31800 | | | | | | beta-ring hydroxylase [EC:1.14.-.-] | | | | | | CYP97A3;cytochrome P450, family 97, subfamily A, polypeptide 3 | | | | | | | | | | | | | | Protein LUTEIN DEFICIENT 5, chloroplastic | | | | | | | | | | | TCONS_00003357 | | | | | | 2.73 | | | | | | | 0.000155 | | | | | |
|  |  |  |  |  |  |  |  |  |  |  |  |  |  |  |  |  |  |  |  |  |  |  |  |  |  |  |  |  |  |  | AT3G53130 | | | | | | carotenoid epsilon hydroxylase [EC:1.14.14.158] | | | | | | LUT1;Cytochrome P450 superfamily protein | | | | | | | | | | | | | | Carotene epsilon-monooxygenase, chloroplastic | | | | | | | | | | | TCONS_00001066 | | | | | | 2.60 | | | | | | | 0.000184 | | | | | |
|  |  |  |  |  |  |  |  |  |  |  |  |  |  |  |  |  |  |  |  |  |  |  |  |  |  |  |  |  |  |  | AT4G14210 | | | | | | 15-cis-phytoene desaturase [EC:1.3.5.5] | | | | | | PDS3; phytoene desaturase 3 | | | | | | | | | | | | | | 15-cis-phytoene desaturase, chloroplastic/chromoplastic | | | | | | | | | | | TCONS_00011387 | | | | | | 1.56 | | | | | | | 0.000197 | | | | | |
|  | ath00500 | | | | | | | Starch and sucrose metabolism | | | | | | | 7.56E-03 | | | | | | | | | | 24 | | | | | | AT1G02850 | | | | | | beta-glucosidase [EC:3.2.1.21] | | | | | | BGLU11; beta glucosidase 11 | | | | | | | | | | | | | | Beta-glucosidase 11 | | | | | | | | | | | TCONS_00026109 | | | | | | 1.23 | | | | | | | 0.001007 | | | | | |
|  |  |  |  |  |  |  |  |  |  |  |  |  |  |  |  |  |  |  |  |  |  |  |  |  |  |  |  |  |  |  | AT1G03310 | | | | | | isoamylase [EC:3.2.1.68] | | | | | | DBE1; debranching enzyme 1 | | | | | | | | | | | | | | Isoamylase 2, chloroplastic | | | | | | | | | | | TCONS_00025824 | | | | | | 2.29 | | | | | | | 1.59E-08 | | | | | |
|  |  |  |  |  |  |  |  |  |  |  |  |  |  |  |  |  |  |  |  |  |  |  |  |  |  |  |  |  |  |  | AT1G11820 | | | | | | glucan endo-1,3-beta-glucosidase 1/2/3 [EC:3.2.1.39] | | | | | | O-Glycosyl hydrolases family | | | | | | | | | | | | | | Glucan endo-1,3-beta-glucosidase 1 | | | | | | | | | | | TCONS_00019847 | | | | | | 1.26 | | | | | | | 0.020803 | | | | | |
|  |  |  |  |  |  |  |  |  |  |  |  |  |  |  |  |  |  |  |  |  |  |  |  |  |  |  |  |  |  |  | AT1G35910 | | | | | | trehalose 6-phosphate phosphatase [EC:3.1.3.12] | | | | | | TPPD; Haloacid dehalogenase-like h | | | | | | | | | | | | | | Probable trehalose-phosphate phosphatase 4 | | | | | | | | | | | TCONS_00017065 | | | | | | -2.12 | | | | | | | 0.00041 | | | | | |
|  |  |  |  |  |  |  |  |  |  |  |  |  |  |  |  |  |  |  |  |  |  |  |  |  |  |  |  |  |  |  | AT1G60140 | | | | | | trehalose 6-phosphate synthase/phosphatase [EC:2.4.1.15 3.1.3.12] | | | | | | TPS10; trehalose | | | | | | | | | | | | | | Probable alpha,alpha-trehalose-phosphate synthase [UDP-forming] 9 | | | | | | | | | | | TCONS_00010562 | | | | | | -1.77 | | | | | | | 0.001779 | | | | | |
|  |  |  |  |  |  |  |  |  |  |  |  |  |  |  |  |  |  |  |  |  |  |  |  |  |  |  |  |  |  |  | AT1G64390 | | | | | | endoglucanase [EC:3.2.1.4] | | | | | | GH9C2; glycosyl hydrolase 9C2 | | | | | | | | | | | | | | Endoglucanase 6 | | | | | | | | | | | TCONS_00006210 | | | | | | 1.98 | | | | | | | 2.81E-10 | | | | | |
|  |  |  |  |  |  |  |  |  |  |  |  |  |  |  |  |  |  |  |  |  |  |  |  |  |  |  |  |  |  |  | AT1G66430 | | | | | | fructokinase [EC:2.7.1.4] | | | | | | pfkB-like carbohydrate kinase family protein | | | | | | | | | | | | | | Probable fructokinase-6, chloroplastic | | | | | | | | | | | TCONS_00005354 | | | | | | 2.46 | | | | | | | 0.000159 | | | | | |
|  |  |  |  |  |  |  |  |  |  |  |  |  |  |  |  |  |  |  |  |  |  |  |  |  |  |  |  |  |  |  | AT1G69830 | | | | | | alpha-amylase [EC:3.2.1.1] | | | | | | AMY3; alpha-amylase-like 3 | | | | | | | | | | | | | | Alpha-amylase 3, chloroplastic | | | | | | | | | | | TCONS_00010859 | | | | | | 1.28 | | | | | | | 0.0334 | | | | | |
|  |  |  |  |  |  |  |  |  |  |  |  |  |  |  |  |  |  |  |  |  |  |  |  |  |  |  |  |  |  |  | AT1G71380 | | | | | | endoglucanase [EC:3.2.1.4] | | | | | | CEL3; cellulase 3 | | | | | | | | | | | | | | Endoglucanase 9 | | | | | | | | | | | TCONS_00009972 | | | | | | -1.94 | | | | | | | 0.00904 | | | | | |
|  |  |  |  |  |  |  |  |  |  |  |  |  |  |  |  |  |  |  |  |  |  |  |  |  |  |  |  |  |  |  | AT1G76130 | | | | | | alpha-amylase [EC:3.2.1.1] | | | | | | AMY2; alpha-amylase-like 2 | | | | | | | | | | | | | | Probable alpha-amylase 2 | | | | | | | | | | | TCONS_00023028 | | | | | | 1.91 | | | | | | | 0.024028 | | | | | |
|  |  |  |  |  |  |  |  |  |  |  |  |  |  |  |  |  |  |  |  |  |  |  |  |  |  |  |  |  |  |  | AT1G78580 | | | | | | trehalose 6-phosphate synthase/phosphatase [EC:2.4.1.15 3.1.3.12] | | | | | | TPS1; trehalose- | | | | | | | | | | | | | | Alpha,alpha-trehalose-phosphate synthase [UDP-forming] 1 | | | | | | | | | | | TCONS_00025577 | | | | | | 2.14 | | | | | | | 0.011511 | | | | | |
|  |  |  |  |  |  |  |  |  |  |  |  |  |  |  |  |  |  |  |  |  |  |  |  |  |  |  |  |  |  |  | AT2G01630 | | | | | | glucan endo-1,3-beta-glucosidase 1/2/3 [EC:3.2.1.39] | | | | | | O-Glycosyl hydrolases family | | | | | | | | | | | | | | Glucan endo-1,3-beta-glucosidase 3 | | | | | | | | | | | TCONS_00013313 | | | | | | -1.78 | | | | | | | 0.015767 | | | | | |
|  |  |  |  |  |  |  |  |  |  |  |  |  |  |  |  |  |  |  |  |  |  |  |  |  |  |  |  |  |  |  | AT2G18700 | | | | | | trehalose 6-phosphate synthase/phosphatase [EC:2.4.1.15 3.1.3.12] | | | | | | TPS11; trehalose | | | | | | | | | | | | | | Probable alpha,alpha-trehalose-phosphate synthase [UDP-forming] 11 | | | | | | | | | | | TCONS_00018992 | | | | | | -2.26 | | | | | | | 0.027831 | | | | | |
|  |  |  |  |  |  |  |  |  |  |  |  |  |  |  |  |  |  |  |  |  |  |  |  |  |  |  |  |  |  |  | AT2G39930 | | | | | | isoamylase [EC:3.2.1.68] | | | | | | ISA1; isoamylase 1 | | | | | | | | | | | | | | Isoamylase 1, chloroplastic | | | | | | | | | | | TCONS_00021705 | | | | | | 2.71 | | | | | | | 0.000192 | | | | | |
|  |  |  |  |  |  |  |  |  |  |  |  |  |  |  |  |  |  |  |  |  |  |  |  |  |  |  |  |  |  |  | AT2G40840 | | | | | | 4-alpha-glucanotransferase [EC:2.4.1.25] | | | | | | DPE2; disproportionating enzyme 2 | | | | | | | | | | | | | | 4-alpha-glucanotransferase DPE2 | | | | | | | | | | | TCONS_00023616 | | | | | | 2.83 | | | | | | | 4.22E-05 | | | | | |
|  |  |  |  |  |  |  |  |  |  |  |  |  |  |  |  |  |  |  |  |  |  |  |  |  |  |  |  |  |  |  | AT3G13560 | | | | | | glucan endo-1,3-beta-glucosidase 4 [EC:3.2.1.39] | | | | | | O-Glycosyl hydrolases family 17 p | | | | | | | | | | | | | | Glucan endo-1,3-beta-glucosidase 4 | | | | | | | | | | | TCONS_00017729 | | | | | | 1.24 | | | | | | | 0.008983 | | | | | |
|  |  |  |  |  |  |  |  |  |  |  |  |  |  |  |  |  |  |  |  |  |  |  |  |  |  |  |  |  |  |  | AT3G18080 | | | | | | beta-glucosidase [EC:3.2.1.21] | | | | | | BGLU44; B-S glucosidase 44 | | | | | | | | | | | | | | Beta-glucosidase 44 | | | | | | | | | | | TCONS_00020852 | | | | | | 2.54 | | | | | | | 0.000651 | | | | | |
|  |  |  |  |  |  |  |  |  |  |  |  |  |  |  |  |  |  |  |  |  |  |  |  |  |  |  |  |  |  |  | AT3G20440 | | | | | | 1,4-alpha-glucan branching enzyme [EC:2.4.1.18] | | | | | | EMB2729; Alpha amylase family prot | | | | | | | | | | | | | | 1,4-alpha-glucan-branching enzyme 3, chloroplastic/amyloplastic | | | | | | | | | | | TCONS_00026807 | | | | | | 1.87 | | | | | | | 0.023629 | | | | | |
|  |  |  |  |  |  |  |  |  |  |  |  |  |  |  |  |  |  |  |  |  |  |  |  |  |  |  |  |  |  |  | AT3G23920 | | | | | | beta-amylase [EC:3.2.1.2] | | | | | | BAM1; beta-amylase 1 | | | | | | | | | | | | | | Beta-amylase 1, chloroplastic | | | | | | | | | | | TCONS_00016716 | | | | | | 2.27 | | | | | | | 0.001305 | | | | | |
|  |  |  |  |  |  |  |  |  |  |  |  |  |  |  |  |  |  |  |  |  |  |  |  |  |  |  |  |  |  |  | AT4G17090 | | | | | | beta-amylase [EC:3.2.1.2] | | | | | | CT-BMY; chloroplast beta-amylase | | | | | | | | | | | | | | Beta-amylase 3, chloroplastic | | | | | | | | | | | TCONS_00007564 | | | | | | 2.09 | | | | | | | 0.006367 | | | | | |
|  |  |  |  |  |  |  |  |  |  |  |  |  |  |  |  |  |  |  |  |  |  |  |  |  |  |  |  |  |  |  | AT4G18240 | | | | | | starch synthase [EC:2.4.1.21] | | | | | | SS4; starch synthase 4 | | | | | | | | | | | | | | Probable starch synthase 4, chloroplastic/amyloplastic | | | | | | | | | | | TCONS_00020048 | | | | | | 1.70 | | | | | | | 0.001751 | | | | | |
|  |  |  |  |  |  |  |  |  |  |  |  |  |  |  |  |  |  |  |  |  |  |  |  |  |  |  |  |  |  |  | AT4G22590 | | | | | | trehalose 6-phosphate phosphatase [EC:3.1.3.12] | | | | | | TPPG; Haloacid dehalogenase-like h | | | | | | | | | | | | | | Probable trehalose-phosphate phosphatase G | | | | | | | | | | | TCONS_00016062 | | | | | | -2.89 | | | | | | | 0.024742 | | | | | |
|  |  |  |  |  |  |  |  |  |  |  |  |  |  |  |  |  |  |  |  |  |  |  |  |  |  |  |  |  |  |  | AT4G37840 | | | | | | hexokinase [EC:2.7.1.1] | | | | | | HKL3; hexokinase-like 3 | | | | | | | | | | | | | | Probable hexokinase-like 2 protein | | | | | | | | | | | TCONS_00006944 | | | | | | 2.78 | | | | | | | 0.003766 | | | | | |
|  |  |  |  |  |  |  |  |  |  |  |  |  |  |  |  |  |  |  |  |  |  |  |  |  |  |  |  |  |  |  | AT5G45300 | | | | | | beta-amylase [EC:3.2.1.2] | | | | | | BMY2; beta-amylase 2 | | | | | | | | | | | | | | Beta-amylase 8 | | | | | | | | | | | TCONS_00006347 | | | | | | 1.08 | | | | | | | 0.031253 | | | | | |
|  | ath00860 | | | | | | | Porphyrin and chlorophyll metabolism | | | | | | | 8.88E-03 | | | | | | | | | | 9 | | | | | | AT1G04620 | | | | | | 7-hydroxymethyl chlorophyll a reductase [EC:1.17.7.2] | | | | | | HCAR; coenzyme F420 hydrogen | | | | | | | | | | | | | | 7-hydroxymethyl chlorophyll a reductase, chloroplastic | | | | | | | | | | | TCONS_00016669 | | | | | | 3.62 | | | | | | | 0.007027 | | | | | |
|  |  |  |  |  |  |  |  |  |  |  |  |  |  |  |  |  |  |  |  |  |  |  |  |  |  |  |  |  |  |  | AT1G69740 | | | | | | porphobilinogen synthase [EC:4.2.1.24] | | | | | | HEMB1; Aldolase superfamily protein | | | | | | | | | | | | | | Delta-aminolevulinic acid dehydratase 1, chloroplastic | | | | | | | | | | | TCONS_00022475 | | | | | | 1.76 | | | | | | | 0.000206 | | | | | |
|  |  |  |  |  |  |  |  |  |  |  |  |  |  |  |  |  |  |  |  |  |  |  |  |  |  |  |  |  |  |  | AT2G26670 | | | | | | heme oxygenase (biliverdin-producing, ferredoxin) [EC:1.14.15.20] | | | | | | TED4; Plant heme | | | | | | | | | | | | | | Heme oxygenase 1, chloroplastic | | | | | | | | | | | TCONS_00020128 | | | | | | -2.77 | | | | | | | 0.033562 | | | | | |
|  |  |  |  |  |  |  |  |  |  |  |  |  |  |  |  |  |  |  |  |  |  |  |  |  |  |  |  |  |  |  | AT3G14930 | | | | | | uroporphyrinogen decarboxylase [EC:4.1.1.37] | | | | | | HEME1; Uroporphyrinogen decarboxylase | | | | | | | | | | | | | | Uroporphyrinogen decarboxylase 1, chloroplastic | | | | | | | | | | | TCONS_00020396 | | | | | | 2.79 | | | | | | | 1.45E-06 | | | | | |
|  |  |  |  |  |  |  |  |  |  |  |  |  |  |  |  |  |  |  |  |  |  |  |  |  |  |  |  |  |  |  | AT4G01690 | | | | | | protoporphyrinogen/coproporphyrinogen III oxidase [EC:1.3.3.4 1.3.3.15] | | | | | | PPOX; Flav | | | | | | | | | | | | | | Protoporphyrinogen oxidase 1, chloroplastic | | | | | | | | | | | TCONS_00024481 | | | | | | 1.88 | | | | | | | 0.000153 | | | | | |
|  |  |  |  |  |  |  |  |  |  |  |  |  |  |  |  |  |  |  |  |  |  |  |  |  |  |  |  |  |  |  | AT4G25080 | | | | | | magnesium-protoporphyrin O-methyltransferase [EC:2.1.1.11] | | | | | | CHLM; magnesium-protopo | | | | | | | | | | | | | | Magnesium protoporphyrin IX methyltransferase, chloroplastic | | | | | | | | | | | TCONS_00009544 | | | | | | 3.02 | | | | | | | 0.001081 | | | | | |
|  |  |  |  |  |  |  |  |  |  |  |  |  |  |  |  |  |  |  |  |  |  |  |  |  |  |  |  |  |  |  | AT4G37000 | | | | | | red chlorophyll catabolite reductase [EC:1.3.7.12] | | | | | | ACD2; accelerated cell death 2 | | | | | | | | | | | | | | Red chlorophyll catabolite reductase, chloroplastic | | | | | | | | | | | TCONS_00003980 | | | | | | 1.83 | | | | | | | 0.033321 | | | | | |
|  |  |  |  |  |  |  |  |  |  |  |  |  |  |  |  |  |  |  |  |  |  |  |  |  |  |  |  |  |  |  | AT5G04900 | | | | | | chlorophyll(ide) b reductase [EC:1.1.1.294] | | | | | | NOL; NYC1-like protein | | | | | | | | | | | | | | Chlorophyll(ide) b reductase NOL, chloroplastic | | | | | | | | | | | TCONS_00010756 | | | | | | 3.01 | | | | | | | 0.00953 | | | | | |
|  |  |  |  |  |  |  |  |  |  |  |  |  |  |  |  |  |  |  |  |  |  |  |  |  |  |  |  |  |  |  | AT5G43860 | | | | | | chlorophyllase [EC:3.1.1.14] | | | | | | CLH2; chlorophyllase 2 | | | | | | | | | | | | | | Chlorophyllase-2, chloroplastic | | | | | | | | | | | TCONS_00023404 | | | | | | 2.39 | | | | | | | 0.000942 | | | | | |
|  | ath00190 | | | | | | | Oxidative phosphorylation | | | | | | | 9.83E-03 | | | | | | | | | | 6 | | | | | | AT1G20260 | | | | | | V-type H+-transporting ATPase subunit B | | | | | | VAB3; ATPase, V1 complex, subunit B protei | | | | | | | | | | | | | | V-type proton ATPase subunit B3 | | | | | | | | | | | TCONS_00016986 | | | | | | 1.07 | | | | | | | 8.81E-05 | | | | | |
|  |  |  |  |  |  |  |  |  |  |  |  |  |  |  |  |  |  |  |  |  |  |  |  |  |  |  |  |  |  |  | AT3G53620 | | | | | | inorganic pyrophosphatase [EC:3.6.1.1] | | | | | | PPa4; pyrophosphorylase 4 | | | | | | | | | | | | | | Soluble inorganic pyrophosphatase 4 | | | | | | | | | | | TCONS_00001110 | | | | | | 2.26 | | | | | | | 5.18E-07 | | | | | |
|  |  |  |  |  |  |  |  |  |  |  |  |  |  |  |  |  |  |  |  |  |  |  |  |  |  |  |  |  |  |  | AT3G58730 | | | | | | V-type H+-transporting ATPase subunit D | | | | | | vacuolar ATP synthase subunit D (VATD) / V | | | | | | | | | | | | | | V-type proton ATPase subunit D | | | | | | | | | | | TCONS_00002662 | | | | | | 0.77 | | | | | | | 0.017217 | | | | | |
|  |  |  |  |  |  |  |  |  |  |  |  |  |  |  |  |  |  |  |  |  |  |  |  |  |  |  |  |  |  |  | AT4G39080 | | | | | | V-type H+-transporting ATPase subunit a | | | | | | VHA-A3; vacuolar proton ATPase A3 | | | | | | | | | | | | | | V-type proton ATPase subunit a3 | | | | | | | | | | | TCONS_00020583 | | | | | | 1.19 | | | | | | | 0.000108 | | | | | |
|  |  |  |  |  |  |  |  |  |  |  |  |  |  |  |  |  |  |  |  |  |  |  |  |  |  |  |  |  |  |  | AT5G09650 | | | | | | inorganic pyrophosphatase [EC:3.6.1.1] | | | | | | PPa6; pyrophosphorylase 6 | | | | | | | | | | | | | | Soluble inorganic pyrophosphatase 6, chloroplastic | | | | | | | | | | | TCONS_00019245 | | | | | | 1.05 | | | | | | | 0.002521 | | | | | |
|  |  |  |  |  |  |  |  |  |  |  |  |  |  |  |  |  |  |  |  |  |  |  |  |  |  |  |  |  |  |  | AT5G40650 | | | | | | succinate dehydrogenase (ubiquinone) iron-sulfur subunit [EC:1.3.5.1] | | | | | | SDH2-2; succ | | | | | | | | | | | | | | Succinate dehydrogenase [ubiquinone] iron-sulfur subunit 2, mitochondrial | | | | | | | | | | | TCONS_00000102 | | | | | | 1.71 | | | | | | | 0.002198 | | | | | |
|  | ath03060 | | | | | | | Protein export | | | | | | | 1.01E-02 | | | | | | | | | | 8 | | | | | | AT1G06870 | | | | | | signal peptidase I [EC:3.4.21.89] | | | | | | Plsp2A; Peptidase S24/S26A/S26B/S26C family prot | | | | | | | | | | | | | | Probable thylakoidal processing peptidase 2, chloroplastic | | | | | | | | | | | TCONS_00020113 | | | | | | -1.25 | | | | | | | 0.004566 | | | | | |
|  |  |  |  |  |  |  |  |  |  |  |  |  |  |  |  |  |  |  |  |  |  |  |  |  |  |  |  |  |  |  | AT1G24490 | | | | | | YidC/Oxa1 family membrane protein insertase | | | | | | ALB4; OxaA/YidC-like membrane insertio | | | | | | | | | | | | | | ALBINO3-like protein 1, chloroplastic | | | | | | | | | | | TCONS_00022529 | | | | | | 1.97 | | | | | | | 0.006422 | | | | | |
|  |  |  |  |  |  |  |  |  |  |  |  |  |  |  |  |  |  |  |  |  |  |  |  |  |  |  |  |  |  |  | AT2G01110 | | | | | | sec-independent protein translocase protein TatC | | | | | | APG2; Sec-independent periplasmic | | | | | | | | | | | | | | Sec-independent protein translocase protein TATC, chloroplastic | | | | | | | | | | | TCONS_00008060 | | | | | | 2.56 | | | | | | | 0.004539 | | | | | |
|  |  |  |  |  |  |  |  |  |  |  |  |  |  |  |  |  |  |  |  |  |  |  |  |  |  |  |  |  |  |  | AT2G18710 | | | | | | protein transport protein SEC61 subunit alpha | | | | | | SCY1; SECY homolog 1 | | | | | | | | | | | | | | Preprotein translocase subunit SCY1, chloroplastic | | | | | | | | | | | TCONS_00021647 | | | | | | 2.72 | | | | | | | 0.00115 | | | | | |
|  |  |  |  |  |  |  |  |  |  |  |  |  |  |  |  |  |  |  |  |  |  |  |  |  |  |  |  |  |  |  | AT2G45070 | | | | | | protein transport protein SEC61 subunit beta | | | | | | SEC61_BETA; Preprotein translocase Se | | | | | | | | | | | | | | Protein transport protein Sec61 subunit beta | | | | | | | | | | | TCONS_00009159 | | | | | | -1.75 | | | | | | | 0.007377 | | | | | |
|  |  |  |  |  |  |  |  |  |  |  |  |  |  |  |  |  |  |  |  |  |  |  |  |  |  |  |  |  |  |  | AT2G45770 | | | | | | fused signal recognition particle receptor | | | | | | CPFTSY; signal recognition particle rec | | | | | | | | | | | | | | Cell division protein FtsY homolog, chloroplastic | | | | | | | | | | | TCONS_00007349 | | | | | | 1.39 | | | | | | | 0.000818 | | | | | |
|  |  |  |  |  |  |  |  |  |  |  |  |  |  |  |  |  |  |  |  |  |  |  |  |  |  |  |  |  |  |  | AT5G03940 | | | | | | signal recognition particle subunit SRP54 [EC:3.6.5.4] | | | | | | CPSRP54; chloroplast signal | | | | | | | | | | | | | | Signal recognition particle 54 kDa protein, chloroplastic | | | | | | | | | | | TCONS_00024675 | | | | | | 3.03 | | | | | | | 0.001703 | | | | | |
|  |  |  |  |  |  |  |  |  |  |  |  |  |  |  |  |  |  |  |  |  |  |  |  |  |  |  |  |  |  |  | AT5G28750 | | | | | | sec-independent protein translocase protein TatA | | | | | | Bacterial sec-independent translo | | | | | | | | | | | | | | Sec-independent protein translocase protein TATA, chloroplastic | | | | | | | | | | | TCONS_00012604 | | | | | | 2.22 | | | | | | | 0.0004 | | | | | |
| topGO | **L-SA vs R-SA** | | | | | | | | | | | | | | | | | | | | | | | | | | | | | | | | | | | | | | | | | | | | | | | | | | | | | | | | | | | | | | | | | | | | | | | | | | | | | | | | | | | | | |
|  | Up-regulated | | | | | | | | | | | | | | | | | | | | | | | | | | | | | | | | | | | | | | | | | | | | | | | | | | | | | | | | | | | | | | | | | | | | | | | | | | | | | | | | | | | | | |
|  |  | | | | GO.ID | | | | | | | | | | | | | | | | Term | | | | | | | | | | | | | | | | | | | | | | | | | Annotated | | | | | | Significant | | | | | | | | | | | Expected | | | | | | | classicFisher | | | | | | | | q_value | | | | | | | | |
|  | BP | | | | GO:0006457 | | | | | | | | | | | | | | | | protein folding | | | | | | | | | | | | | | | | | | | | | | | | | 212 | | | | | | 25 | | | | | | | | | | | 3.27 | | | | | | | 2.90E-15 | | | | | | | | 8.5E-14 | | | | | | | | |
|  |  |  |  |  | GO:0009408 | | | | | | | | | | | | | | | | response to heat | | | | | | | | | | | | | | | | | | | | | | | | | 214 | | | | | | 19 | | | | | | | | | | | 3.3 | | | | | | | 9.70E-10 | | | | | | | | 1.4E-08 | | | | | | | | |
|  |  |  |  |  | GO:0009644 | | | | | | | | | | | | | | | | response to high light intensity | | | | | | | | | | | | | | | | | | | | | | | | | 61 | | | | | | 9 | | | | | | | | | | | 0.94 | | | | | | | 3.80E-07 | | | | | | | | 3.7E-06 | | | | | | | | |
|  |  |  |  |  | GO:0042026 | | | | | | | | | | | | | | | | protein refolding | | | | | | | | | | | | | | | | | | | | | | | | | 35 | | | | | | 7 | | | | | | | | | | | 0.54 | | | | | | | 9.00E-07 | | | | | | | | 6.6E-06 | | | | | | | | |
|  |  |  |  |  | GO:0000302 | | | | | | | | | | | | | | | | response to reactive oxygen species | | | | | | | | | | | | | | | | | | | | | | | | | 152 | | | | | | 12 | | | | | | | | | | | 2.35 | | | | | | | 4.30E-06 | | | | | | | | 2.3E-05 | | | | | | | | |
|  |  |  |  |  | GO:0042542 | | | | | | | | | | | | | | | | response to hydrogen peroxide | | | | | | | | | | | | | | | | | | | | | | | | | 62 | | | | | | 8 | | | | | | | | | | | 0.96 | | | | | | | 4.80E-06 | | | | | | | | 2.3E-05 | | | | | | | | |
|  |  |  |  |  | GO:0009266 | | | | | | | | | | | | | | | | response to temperature stimulus | | | | | | | | | | | | | | | | | | | | | | | | | 484 | | | | | | 20 | | | | | | | | | | | 7.47 | | | | | | | 6.80E-05 | | | | | | | | 2.5E-04 | | | | | | | | |
|  |  |  |  |  | GO:0009642 | | | | | | | | | | | | | | | | response to light intensity | | | | | | | | | | | | | | | | | | | | | | | | | 114 | | | | | | 9 | | | | | | | | | | | 1.76 | | | | | | | 6.90E-05 | | | | | | | | 2.5E-04 | | | | | | | | |
|  |  |  |  |  | GO:0034620 | | | | | | | | | | | | | | | | cellular response to unfolded protein | | | | | | | | | | | | | | | | | | | | | | | | | 23 | | | | | | 4 | | | | | | | | | | | 0.35 | | | | | | | 0.00039 | | | | | | | | 1.2E-03 | | | | | | | | |
|  |  |  |  |  | GO:0052033 | | | | | | | | | | | | | | | | pathogen-associated molecular pattern de... | | | | | | | | | | | | | | | | | | | | | | | | | 10 | | | | | | 3 | | | | | | | | | | | 0.15 | | | | | | | 0.0004 | | | | | | | | 1.2E-03 | | | | | | | | |
|  | MF | | | | GO:0051082 | | | | | | | | | | | | | | | | unfolded protein binding | | | | | | | | | | | | | | | | | | | | | | | | | 139 | | | | | | 18 | | | | | | | | | | | 2.06 | | | | | | | 2.50E-12 | | | | | | | | 7.3E-11 | | | | | | | | |
|  |  |  |  |  | GO:0031625 | | | | | | | | | | | | | | | | ubiquitin protein ligase binding | | | | | | | | | | | | | | | | | | | | | | | | | 50 | | | | | | 5 | | | | | | | | | | | 0.74 | | | | | | | 0.00085 | | | | | | | | 1.0E-02 | | | | | | | | |
|  |  |  |  |  | GO:0044389 | | | | | | | | | | | | | | | | ubiquitin-like protein ligase binding | | | | | | | | | | | | | | | | | | | | | | | | | 56 | | | | | | 5 | | | | | | | | | | | 0.83 | | | | | | | 0.00142 | | | | | | | | 1.0E-02 | | | | | | | | |
|  |  |  |  |  | GO:0017111 | | | | | | | | | | | | | | | | nucleoside-triphosphatase activity | | | | | | | | | | | | | | | | | | | | | | | | | 784 | | | | | | 23 | | | | | | | | | | | 11.61 | | | | | | | 0.00148 | | | | | | | | 1.0E-02 | | | | | | | | |
|  |  |  |  |  | GO:0016887 | | | | | | | | | | | | | | | | ATPase activity | | | | | | | | | | | | | | | | | | | | | | | | | 469 | | | | | | 16 | | | | | | | | | | | 6.95 | | | | | | | 0.00178 | | | | | | | | 1.0E-02 | | | | | | | | |
|  |  |  |  |  | GO:0031072 | | | | | | | | | | | | | | | | heat shock protein binding | | | | | | | | | | | | | | | | | | | | | | | | | 63 | | | | | | 5 | | | | | | | | | | | 0.93 | | | | | | | 0.0024 | | | | | | | | 1.2E-02 | | | | | | | | |
|  |  |  |  |  | GO:0030628 | | | | | | | | | | | | | | | | pre-mRNA 3'-splice site binding | | | | | | | | | | | | | | | | | | | | | | | | | 6 | | | | | | 2 | | | | | | | | | | | 0.09 | | | | | | | 0.00315 | | | | | | | | 1.2E-02 | | | | | | | | |
|  |  |  |  |  | GO:0008237 | | | | | | | | | | | | | | | | metallopeptidase activity | | | | | | | | | | | | | | | | | | | | | | | | | 98 | | | | | | 6 | | | | | | | | | | | 1.45 | | | | | | | 0.00338 | | | | | | | | 1.2E-02 | | | | | | | | |
|  |  |  |  |  | GO:0016462 | | | | | | | | | | | | | | | | pyrophosphatase activity | | | | | | | | | | | | | | | | | | | | | | | | | 849 | | | | | | 23 | | | | | | | | | | | 12.57 | | | | | | | 0.00402 | | | | | | | | 1.3E-02 | | | | | | | | |
|  |  |  |  |  | GO:0016818 | | | | | | | | | | | | | | | | hydrolase activity, acting on acid anhyd... | | | | | | | | | | | | | | | | | | | | | | | | | 857 | | | | | | 23 | | | | | | | | | | | 12.69 | | | | | | | 0.0045 | | | | | | | | 1.3E-02 | | | | | | | | |
|  | CC | | | | GO:0005788 | | | | | | | | | | | | | | | | endoplasmic reticulum lumen | | | | | | | | | | | | | | | | | | | | | | | | | 31 | | | | | | 7 | | | | | | | | | | | 0.46 | | | | | | | 2.70E-07 | | | | | | | | 7.9E-06 | | | | | | | | |
|  |  |  |  |  | GO:0034663 | | | | | | | | | | | | | | | | endoplasmic reticulum chaperone complex | | | | | | | | | | | | | | | | | | | | | | | | | 3 | | | | | | 2 | | | | | | | | | | | 0.04 | | | | | | | 0.00064 | | | | | | | | 9.4E-03 | | | | | | | | |
|  |  |  |  |  | GO:0009941 | | | | | | | | | | | | | | | | chloroplast envelope | | | | | | | | | | | | | | | | | | | | | | | | | 536 | | | | | | 18 | | | | | | | | | | | 7.91 | | | | | | | 0.00107 | | | | | | | | 9.4E-03 | | | | | | | | |
|  |  |  |  |  | GO:0009526 | | | | | | | | | | | | | | | | plastid envelope | | | | | | | | | | | | | | | | | | | | | | | | | 545 | | | | | | 18 | | | | | | | | | | | 8.04 | | | | | | | 0.00129 | | | | | | | | 9.4E-03 | | | | | | | | |
|  |  |  |  |  | GO:0019005 | | | | | | | | | | | | | | | | SCF ubiquitin ligase complex | | | | | | | | | | | | | | | | | | | | | | | | | 67 | | | | | | 5 | | | | | | | | | | | 0.99 | | | | | | | 0.00309 | | | | | | | | 1.8E-02 | | | | | | | | |
|  |  |  |  |  | GO:0031969 | | | | | | | | | | | | | | | | chloroplast membrane | | | | | | | | | | | | | | | | | | | | | | | | | 290 | | | | | | 11 | | | | | | | | | | | 4.28 | | | | | | | 0.00401 | | | | | | | | 1.9E-02 | | | | | | | | |
|  |  |  |  |  | GO:0042170 | | | | | | | | | | | | | | | | plastid membrane | | | | | | | | | | | | | | | | | | | | | | | | | 295 | | | | | | 11 | | | | | | | | | | | 4.35 | | | | | | | 0.00455 | | | | | | | | 1.9E-02 | | | | | | | | |
|  |  |  |  |  | GO:0000943 | | | | | | | | | | | | | | | | retrotransposon nucleocapsid | | | | | | | | | | | | | | | | | | | | | | | | | 10 | | | | | | 2 | | | | | | | | | | | 0.15 | | | | | | | 0.00902 | | | | | | | | 3.3E-02 | | | | | | | | |
|  |  |  |  |  | GO:0031514 | | | | | | | | | | | | | | | | motile cilium | | | | | | | | | | | | | | | | | | | | | | | | | 11 | | | | | | 2 | | | | | | | | | | | 0.16 | | | | | | | 0.01092 | | | | | | | | 3.6E-02 | | | | | | | | |
|  |  |  |  |  | GO:0009507 | | | | | | | | | | | | | | | | chloroplast | | | | | | | | | | | | | | | | | | | | | | | | | 2298 | | | | | | 47 | | | | | | | | | | | 33.9 | | | | | | | 0.01258 | | | | | | | | 3.7E-02 | | | | | | | | |
|  | Down-regulated | | | | | | | | | | | | | | | | | | | | | | | | | | | | | | | | | | | | | | | | | | | | | | | | | | | | | | | | | | | | | | | | | | | | | | | | | | | | | | | | | | | | | |
|  | BP | | | | GO:0043604 | | | | | | | | | | | | | | | | amide biosynthetic process | | | | | | | | | | | | | | | | | | | | | | | | | 732 | | | | | | 23 | | | | | | | | | | | 8.46 | | | | | | | 1.40E-05 | | | | | | | | 2.2E-04 | | | | | | | | |
|  |  |  |  |  | GO:0043603 | | | | | | | | | | | | | | | | cellular amide metabolic process | | | | | | | | | | | | | | | | | | | | | | | | | 857 | | | | | | 25 | | | | | | | | | | | 9.91 | | | | | | | 2.00E-05 | | | | | | | | 2.2E-04 | | | | | | | | |
|  |  |  |  |  | GO:0006412 | | | | | | | | | | | | | | | | translation | | | | | | | | | | | | | | | | | | | | | | | | | 670 | | | | | | 21 | | | | | | | | | | | 7.75 | | | | | | | 3.50E-05 | | | | | | | | 2.2E-04 | | | | | | | | |
|  |  |  |  |  | GO:0043043 | | | | | | | | | | | | | | | | peptide biosynthetic process | | | | | | | | | | | | | | | | | | | | | | | | | 673 | | | | | | 21 | | | | | | | | | | | 7.78 | | | | | | | 3.70E-05 | | | | | | | | 2.2E-04 | | | | | | | | |
|  |  |  |  |  | GO:0006518 | | | | | | | | | | | | | | | | peptide metabolic process | | | | | | | | | | | | | | | | | | | | | | | | | 726 | | | | | | 22 | | | | | | | | | | | 8.39 | | | | | | | 3.80E-05 | | | | | | | | 2.2E-04 | | | | | | | | |
|  |  |  |  |  | GO:0000463 | | | | | | | | | | | | | | | | maturation of LSU-rRNA from tricistronic... | | | | | | | | | | | | | | | | | | | | | | | | | 16 | | | | | | 3 | | | | | | | | | | | 0.18 | | | | | | | 0.00076 | | | | | | | | 3.7E-03 | | | | | | | | |
|  |  |  |  |  | GO:0008272 | | | | | | | | | | | | | | | | sulfate transport | | | | | | | | | | | | | | | | | | | | | | | | | 18 | | | | | | 3 | | | | | | | | | | | 0.21 | | | | | | | 0.00109 | | | | | | | | 4.6E-03 | | | | | | | | |
|  |  |  |  |  | GO:0006839 | | | | | | | | | | | | | | | | mitochondrial transport | | | | | | | | | | | | | | | | | | | | | | | | | 78 | | | | | | 5 | | | | | | | | | | | 0.9 | | | | | | | 0.0021 | | | | | | | | 7.7E-03 | | | | | | | | |
|  |  |  |  |  | GO:0045910 | | | | | | | | | | | | | | | | negative regulation of DNA recombination | | | | | | | | | | | | | | | | | | | | | | | | | 24 | | | | | | 3 | | | | | | | | | | | 0.28 | | | | | | | 0.00258 | | | | | | | | 7.9E-03 | | | | | | | | |
|  | MF | | | | GO:0003735 | | | | | | | | | | | | | | | | structural constituent of ribosome | | | | | | | | | | | | | | | | | | | | | | | | | 384 | | | | | | 21 | | | | | | | | | | | 4.22 | | | | | | | 1.70E-09 | | | | | | | | 5.0E-08 | | | | | | | | |
|  |  |  |  |  | GO:0005198 | | | | | | | | | | | | | | | | structural molecule activity | | | | | | | | | | | | | | | | | | | | | | | | | 518 | | | | | | 22 | | | | | | | | | | | 5.69 | | | | | | | 6.70E-08 | | | | | | | | 9.8E-07 | | | | | | | | |
|  |  |  |  |  | GO:0003954 | | | | | | | | | | | | | | | | NADH dehydrogenase activity | | | | | | | | | | | | | | | | | | | | | | | | | 62 | | | | | | 6 | | | | | | | | | | | 0.68 | | | | | | | 6.00E-05 | | | | | | | | 5.9E-04 | | | | | | | | |
|  |  |  |  |  | GO:1901682 | | | | | | | | | | | | | | | | sulfur compound transmembrane transporte... | | | | | | | | | | | | | | | | | | | | | | | | | 25 | | | | | | 4 | | | | | | | | | | | 0.27 | | | | | | | 0.00015 | | | | | | | | 1.1E-03 | | | | | | | | |
|  |  |  |  |  | GO:0008121 | | | | | | | | | | | | | | | | ubiquinol-cytochrome-c reductase activit... | | | | | | | | | | | | | | | | | | | | | | | | | 13 | | | | | | 3 | | | | | | | | | | | 0.14 | | | | | | | 0.00035 | | | | | | | | 1.7E-03 | | | | | | | | |
|  |  |  |  |  | GO:0016681 | | | | | | | | | | | | | | | | oxidoreductase activity, acting on diphe... | | | | | | | | | | | | | | | | | | | | | | | | | 13 | | | | | | 3 | | | | | | | | | | | 0.14 | | | | | | | 0.00035 | | | | | | | | 1.7E-03 | | | | | | | | |
|  |  |  |  |  | GO:0015116 | | | | | | | | | | | | | | | | sulfate transmembrane transporter activi... | | | | | | | | | | | | | | | | | | | | | | | | | 14 | | | | | | 3 | | | | | | | | | | | 0.15 | | | | | | | 0.00044 | | | | | | | | 1.8E-03 | | | | | | | | |
|  |  |  |  |  | GO:0016651 | | | | | | | | | | | | | | | | oxidoreductase activity, acting on NAD(P... | | | | | | | | | | | | | | | | | | | | | | | | | 148 | | | | | | 7 | | | | | | | | | | | 1.63 | | | | | | | 0.00127 | | | | | | | | 4.6E-03 | | | | | | | | |
|  |  |  |  |  | GO:0031491 | | | | | | | | | | | | | | | | nucleosome binding | | | | | | | | | | | | | | | | | | | | | | | | | 23 | | | | | | 3 | | | | | | | | | | | 0.25 | | | | | | | 0.00197 | | | | | | | | 6.4E-03 | | | | | | | | |
|  |  |  |  |  | GO:0048038 | | | | | | | | | | | | | | | | quinone binding | | | | | | | | | | | | | | | | | | | | | | | | | 53 | | | | | | 4 | | | | | | | | | | | 0.58 | | | | | | | 0.00273 | | | | | | | | 8.0E-03 | | | | | | | | |
|  | CC | | | | GO:0022626 | | | | | | | | | | | | | | | | cytosolic ribosome | | | | | | | | | | | | | | | | | | | | | | | | | 194 | | | | | | 15 | | | | | | | | | | | 2.32 | | | | | | | 1.30E-08 | | | | | | | | 3.1E-07 | | | | | | | | |
|  |  |  |  |  | GO:1990904 | | | | | | | | | | | | | | | | ribonucleoprotein complex | | | | | | | | | | | | | | | | | | | | | | | | | 882 | | | | | | 32 | | | | | | | | | | | 10.54 | | | | | | | 2.10E-08 | | | | | | | | 3.1E-07 | | | | | | | | |
|  |  |  |  |  | GO:0005840 | | | | | | | | | | | | | | | | ribosome | | | | | | | | | | | | | | | | | | | | | | | | | 456 | | | | | | 22 | | | | | | | | | | | 5.45 | | | | | | | 3.20E-08 | | | | | | | | 3.1E-07 | | | | | | | | |
|  |  |  |  |  | GO:0070469 | | | | | | | | | | | | | | | | respiratory chain | | | | | | | | | | | | | | | | | | | | | | | | | 135 | | | | | | 12 | | | | | | | | | | | 1.61 | | | | | | | 8.00E-08 | | | | | | | | 5.9E-07 | | | | | | | | |
|  |  |  |  |  | GO:0031966 | | | | | | | | | | | | | | | | mitochondrial membrane | | | | | | | | | | | | | | | | | | | | | | | | | 416 | | | | | | 20 | | | | | | | | | | | 4.97 | | | | | | | 1.50E-07 | | | | | | | | 8.8E-07 | | | | | | | | |
|  |  |  |  |  | GO:0044445 | | | | | | | | | | | | | | | | cytosolic part | | | | | | | | | | | | | | | | | | | | | | | | | 241 | | | | | | 15 | | | | | | | | | | | 2.88 | | | | | | | 2.20E-07 | | | | | | | | 9.2E-07 | | | | | | | | |
|  |  |  |  |  | GO:0005739 | | | | | | | | | | | | | | | | mitochondrion | | | | | | | | | | | | | | | | | | | | | | | | | 1239 | | | | | | 37 | | | | | | | | | | | 14.8 | | | | | | | 2.20E-07 | | | | | | | | 9.2E-07 | | | | | | | | |
|  |  |  |  |  | GO:0005740 | | | | | | | | | | | | | | | | mitochondrial envelope | | | | | | | | | | | | | | | | | | | | | | | | | 433 | | | | | | 20 | | | | | | | | | | | 5.17 | | | | | | | 2.80E-07 | | | | | | | | 1.0E-06 | | | | | | | | |
|  |  |  |  |  | GO:0044391 | | | | | | | | | | | | | | | | ribosomal subunit | | | | | | | | | | | | | | | | | | | | | | | | | 240 | | | | | | 14 | | | | | | | | | | | 2.87 | | | | | | | 1.20E-06 | | | | | | | | 3.9E-06 | | | | | | | | |
|  |  |  |  |  | GO:0044429 | | | | | | | | | | | | | | | | mitochondrial part | | | | | | | | | | | | | | | | | | | | | | | | | 545 | | | | | | 21 | | | | | | | | | | | 6.51 | | | | | | | 2.60E-06 | | | | | | | | 7.6E-06 | | | | | | | | |
| KEGG | **Pathway code** | | | | | | | | | | | | **Pathway name** | | | | | | | **p.value** | | | | | | | | **Annotated** | | | | | | **Tair Gene Symbol** | | | | | | **KEGG Gene** | | | **RefSeq** | | | | | | | | | | | | | | | | | ***Lactuca sativa* Gene** | | | | | | | | **Transcript** | | | | | | **FC** | | | | | | | | | | **p-value** | | |
|  | ath04141 | | | | | | | | | | | | Protein processing in endoplasmic reticulum | | | | | | | 0.001957 | | | | | | | | 11 | | | | | | AT1G54050 | | | | | | HSP20 family protein | | | HSP20-like chaperones superfamily protein | | | | | | | | | | | | | | | | | 17.4 kDa class III heat shock protein | | | | | | | | TCONS_00013458 | | | | | | 5.12 | | | | | | | | | | 0.015974 | | |
|  |  |  |  |  |  |  |  |  |  |  |  |  |  |  |  |  |  |  |  |  |  |  |  |  |  |  |  |  |  |  |  |  |  | AT4G25200 | | | | | | HSP20 family protein | | | HSP23.6-MITO; mitochondrion-localized small heat shock protein 23.6 | | | | | | | | | | | | | | | | | 23.6 kDa heat shock protein, mitochondrial | | | | | | | | TCONS_00007407 | | | | | | 4.08 | | | | | | | | | | 0.000238 | | |
|  |  |  |  |  |  |  |  |  |  |  |  |  |  |  |  |  |  |  |  |  |  |  |  |  |  |  |  |  |  |  |  |  |  | AT3G12580 | | | | | | heat shock 70kDa protein 1/2/6/8 | | | HSP70; heat shock protein 70 | | | | | | | | | | | | | | | | | Probable mediator of RNA polymerase II transcription subunit 37c | | | | | | | | TCONS_00023527 | | | | | | 2.25 | | | | | | | | | | 6.35E-06 | | |
|  |  |  |  |  |  |  |  |  |  |  |  |  |  |  |  |  |  |  |  |  |  |  |  |  |  |  |  |  |  |  |  |  |  | AT4G16660 | | | | | | hypoxia up-regulated 1 | | | heat shock protein 70 (Hsp 70) family protein | | | | | | | | | | | | | | | | | Heat shock 70 kDa protein 17 | | | | | | | | TCONS_00006569 | | | | | | 1.29 | | | | | | | | | | 0.022823 | | |
|  |  |  |  |  |  |  |  |  |  |  |  |  |  |  |  |  |  |  |  |  |  |  |  |  |  |  |  |  |  |  |  |  |  | AT3G62600 | | | | | | DnaJ homolog subfamily B member 11 | | | ATERDJ3B; DNAJ heat shock family protein | | | | | | | | | | | | | | | | | DnaJ protein ERDJ3B | | | | | | | | TCONS_00014105 | | | | | | 1.27 | | | | | | | | | | 0.012015 | | |
|  |  |  |  |  |  |  |  |  |  |  |  |  |  |  |  |  |  |  |  |  |  |  |  |  |  |  |  |  |  |  |  |  |  | AT3G07770 | | | | | | heat shock protein 90kDa beta | | | Hsp89.1; HEAT SHOCK PROTEIN 89.1 | | | | | | | | | | | | | | | | | Heat shock protein 90-6, mitochondrial | | | | | | | | TCONS_00026936 | | | | | | 0.90 | | | | | | | | | | 0.005955 | | |
|  |  |  |  |  |  |  |  |  |  |  |  |  |  |  |  |  |  |  |  | 0.043993 | | | | | | | |  |  |  |  |  |  | AT5G59300 | | | | | | ubiquitin-conjugating enzyme E2 G1 [EC:2.3.2.23] | | | UBC7;ubiquitin-conjugating enzyme E2 | | | | | | | | | | | | | | | | | RNA-binding protein 25 | | | | | | | | TCONS_00010569 | | | | | | 0.73 | | | | | | | | | | 0.019903 | | |
|  |  |  |  |  |  |  |  |  |  |  |  |  |  |  |  |  |  |  |  |  |  |  |  |  |  |  |  |  |  |  |  |  |  | AT3G16090 | | | | | | E3 ubiquitin-protein ligase synoviolin [EC:2.3.2.27] | | | Hrd1A;RING/U-box superfamily protein | | | | | | | | | | | | | | | | | ERAD-associated E3 ubiquitin-protein ligase HRD1A | | | | | | | | TCONS_00016204 | | | | | | 0.45 | | | | | | | | | | 0.044306 | | |
|  |  |  |  |  |  |  |  |  |  |  |  |  |  |  |  |  |  |  |  |  |  |  |  |  |  |  |  |  |  |  |  |  |  | AT1G75950 | | | | | | S-phase kinase-associated protein 1 | | | SKP1; S phase kinase-associated protein 1 | | | | | | | | | | | | | | | | | SKP1-like protein 1 | | | | | | | | TCONS_00011215 | | | | | | -0.29 | | | | | | | | | | 0.000138 | | |
|  |  |  |  |  |  |  |  |  |  |  |  |  |  |  |  |  |  |  |  |  |  |  |  |  |  |  |  |  |  |  |  |  |  | AT2G45070 | | | | | | protein transport protein SEC61 subunit beta | | | SEC61_BETA; Preprotein translocase Sec, Sec61-beta subunit protein | | | | | | | | | | | | | | | | | Protein transport protein Sec61 subunit beta | | | | | | | | TCONS_00009159 | | | | | | -0.51 | | | | | | | | | | 2.87E-09 | | |
|  |  |  |  |  |  |  |  |  |  |  |  |  |  |  |  |  |  |  |  |  |  |  |  |  |  |  |  |  |  |  |  |  |  | AT5G12020 | | | | | | HSP20 family protein | | | HSP17.6II; 17.6 kDa class II heat shock protein | | | | | | | | | | | | | | | | | 17.6 kDa class II heat shock protein | | | | | | | | TCONS_00011537 | | | | | | -0.78 | | | | | | | | | | 0.030878 | | |
|  | ath03060 | | | | | | | | | | | | Protein export | | | | | | |  |  |  |  |  |  |  |  | 1 | | | | | | AT2G45070 | | | | | | protein transport protein SEC61 subunit beta | | | SEC61_BETA;Preprotein translocase Sec, Sec61-beta subunit protein | | | | | | | | | | | | | | | | | Protein transport protein Sec61 subunit bet | | | | | | | | TCONS_00009159 | | | | | | -0.51 | | | | | | | | | | 2.87E-09 | | |
|  | ath04145 | | | | | | | | | | | | Phagosome | | | | | | | 0.043993 | | | | | | | | 1 | | | | | | AT2G45070 | | | | | | protein transport protein SEC61 subunit beta | | | SEC61_BETA; Preprotein translocase Sec, Sec61-beta subunit protein | | | | | | | | | | | | | | | | | Protein transport protein Sec61 subunit beta | | | | | | | | TCONS_00009159 | | | | | | -0.51 | | | | | | | | | | 2.87E-09 | | |
|  | ath03410 | | | | | | | | | | | | Base excision repair | | | | | | | 0.083523 | | | | | | | | 1 | | | | | | AT4G02390 | | | | | | poly [ADP-ribose] polymerase [EC:2.4.2.30] | | | PARP2; poly(ADP-ribose) polymerase | | | | | | | | | | | | | | | | | Poly [ADP-ribose] polymerase 2 | | | | | | | | TCONS_00014110 | | | | | | 1.18 | | | | | | | | | | 0.00211 | | |
|  | ath03018 | | | | | | | | | | | | RNA degradation | | | | | | | 0.136019 | | | | | | | | 2 | | | | | | AT3G13470 | | | | | | chaperonin GroEL | | | Cpn60beta2; TCP-1/cpn60 chaperonin family protein | | | | | | | | | | | | | | | | | RuBisCO large subunit-binding protein subunit beta | | | | | | | | TCONS_00003633 | | | | | | 2.39 | | | | | | | | | | 0.024849 | | |
|  |  |  |  |  |  |  |  |  |  |  |  |  |  |  |  |  |  |  |  |  |  |  |  |  |  |  |  |  |  |  |  |  |  | AT5G22250 | | | | | | CCR4-NOT transcription complex subunit 7/8 | | | CAF1b; Polynucleotidyl transferase, ribonuclease H-like superfamily protein | | | | | | | | | | | | | | | | | Probable CCR4-associated factor 1 homolog 11 | | | | | | | | TCONS_00022566 | | | | | | -1.25 | | | | | | | | | | 0.00428 | | |
|  | ath00240 | | | | | | | | | | | | Pyrimidine metabolism | | | | | | | 0.154225 | | | | | | | | 1 | | | | | | AT2G19570 | | | | | | cytidine deaminase [EC:3.5.4.5] | | | CDA1; cytidine deaminase 1 | | | | | | | | | | | | | | | | | Cytidine deaminase 1 | | | | | | | | TCONS_00020080 | | | | | | 1.64 | | | | | | | | | | 0.008399 | | |

**Material and methods for this table - description and reference literature:**

For methods for RNA sequencing and analysis of RNA-seq data see manuscript: Materials and Methods “Transcriptome analysis” subsection [method according to Kęska et al (2021)].

*Reference:*

Kęska, K.; Szcześniak, M.; Makałowska, I.; Czernicka, M. 2021. Long-Term Waterlogging as Factor Contributing to Hypoxia Stress Tolerance Enhancement in Cucumber: Comparative Transcriptome Analysis of Waterlogging Sensitive and Tolerant Accessions. Genes 12, 189. https://doi.org/10.3390/genes12020189.


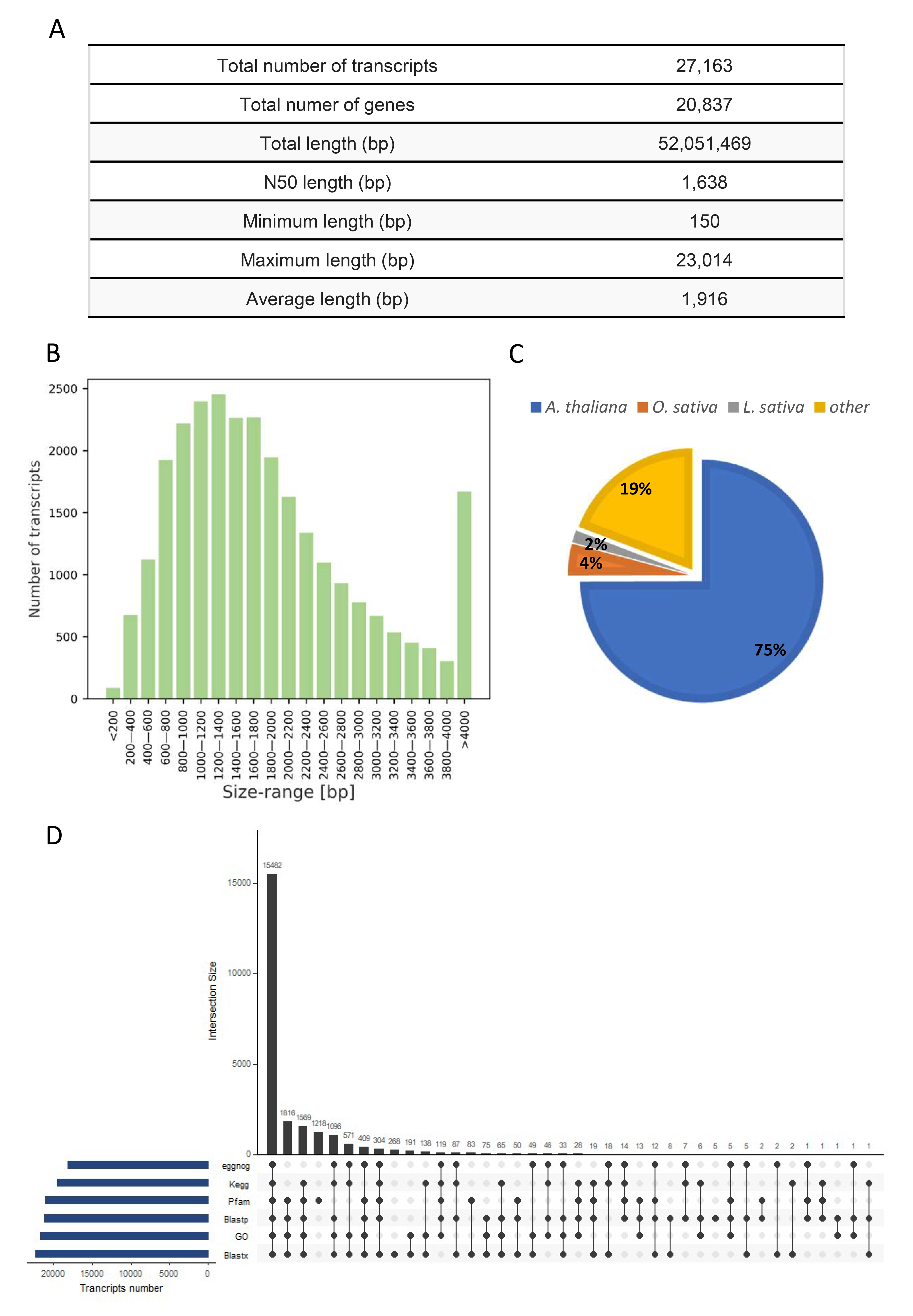


**Figure S1.** Summary of de novo transcriptome assembly of Lactuca sativa L.: A) Numerical summary of the assembled transcriptome; B) Histogram showing distribution of the assembled transcripts; C) Pie chart showing distribution of homologous transcripts across species; D) UpSet plot displaying annotation of lettuce transcripts, according to the results from NCBI (Blastx, Blastp), GO, Pfam, KEGG and eggnog databases.

**Table S2.** Fresh weight of leaves / head/ and roots of lettuce

| **Part of plant** | **Treatment** | **Fresh weight of  leaves /lettuce head/**  **(g)** | **Fresh weight of  lettuce roots**  **(g)** | **Dry weight of leaves**  **(% D.W.)** | **Dry weight of roots**  **(% D.W.)** |
| --- | --- | --- | --- | --- | --- |
| Leaves | Control | **291.4d±35.22** | 31.65a±4.464 | 4.58ab±0.091 | 3.71a±0.073 |
|  | SA | **291.3d±30.11** | 31.36a±3.792 | 4.24a±0.126 | 3.58a±0.148 |
|  | KIO_3_ | **296.8d±34.09** | 34.55a±3.770 | 4.35a±0.081 | 3.57a±0.136 |
|  | KIO_3_+SA | 276.5c±42.20 | 32.61a±3.813 | 4.45a±0.100 | 3.59a±0.337 |
|  | 5-iodosalicylic acid | 171.1ab±35.75 | 39.38a±6.068 | 5.23b±0.150 | **4.02b±0.154** |
|  | 3,5-diiodosalicylic acid | 147.7a±37.79 | 30.12a±5.216 | **6.42c±0.548** | **4.74b±0.308** |

Means in the column followed by different letters differ significantly at P < 0.05 (n=8; means from two cultivation cycles).

**Material and methods for this table:** The harvesting of lettuce plants was conducted in mid-May in each year of study and was followed by the estimation of lettuce head weight. Dry matter content was estimated at 105°C.

**Table S3.** Concentrations of quinoline, 5-chloro-7-iodoquin-8-ol, 5,7-diiodo-8-quinolinol, hydroxychloroquine sulfate, 6-iodo-4-hydroxy-3-quinoline carbocyclic acid, 7-iodo-4-hydroxy-3-quinoline carbocyclic acid and 8-iodo-4-hydroxy-3-quinoline carbocyclic acid in leaves and roots of lettuce as well as root secretions.(RootSec).

**The results of an additional experiment (in year 2021) where no plants were cultivated with the application SA and KIO_3_+SA.**

|  |  | **Quinoline (µg·kg^-1^ D.W.)** | **5-chloro-7-iodoquin-8-ol (µg·kg^-1^ D.W.)** | **5,7-diiodo-8-quinolinol**  **(µg·kg^-1^ D.W.)** | **Hydroxychloroquine**  **(µg·kg^-1^ D.W.)** | **6-iodo-4-hydroxy-3-quinoline carbocyclic acid**  **(µg·kg^-1^ D.W.)** | **7-iodo-4-hydroxy-3-quinoline carbocyclic acid**  **(µg·kg^-1^ D.W.)** | **8-iodo-4-hydroxy-3-quinoline carbocyclic acid**  **(µg·kg^-1^ D.W.)** |
| --- | --- | --- | --- | --- | --- | --- | --- | --- |
| **Part of plant / RootSec*** | **Treatment** | 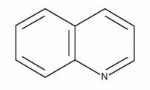 | 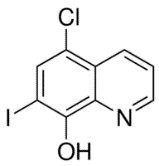 | 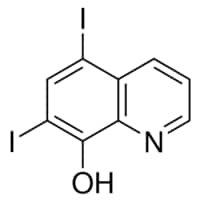 | 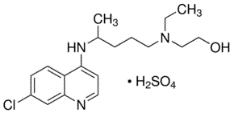 | **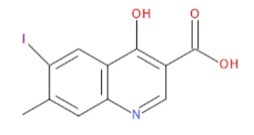** | 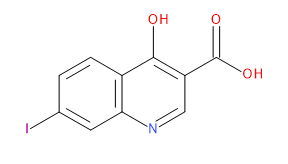 | 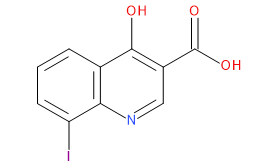 |
|  |  | **Leaves** | | | | | | |
| Leaves | Control | 1,9±0,12a | 4,4±0,25c | 14,12±0a | 47,4±4,37b | 3,5±0,25a | 2,6±0,16a | 44,09±0bc |
|  | KIO_3_ | 5,6±0,41c | 1,9±0,03b | 7,14±0a | **56,3±4,34c** | **9,9±0,14c** | **7,3±0,76b** | 16,18±0a |
|  | 5-iodosalicylic acid | 4,2±0,34b | 1,2±0,1a | 6,62±0a | 35,9±2,09a | **7,6±0,32b** | **7,7±0,31b** | 25,99±0ab |
|  | 3,5-diiodosalicylic acid | **9,6±0,65d** | 1,1±0,04a | 7,57±0a | 34,8±0,88a | **13,8±0,7d** | **8,3±0,21b** | **56,38±0c** |
|  |  | **Roots** | | | | | | |
| Roots | Control | 44,6±3,29a | 40,9±0,26d | 42,8±1,79a | 1,2±0,09a | 11,6±0,86a | 58,6±8,73a | 0±0a |
|  | KIO_3_ | **48,9±1,19b** | 22,3±3,21b | 18,5±2,25a | 1,1±0,05a | 16,8±1,41b | 32,7±9,09a | **9,57±0,704** |
|  | 5-iodosalicylic acid | **56,4±3,81c** | 9,9±1,73a | **513±18,05b** | **2,9±0,23c** | 17,4±1,79b | 37,5±3,66a | 0±0a |
|  | 3,5-diiodosalicylic acid | **53,6±1,95c** | **60,6±2,28c** | 44,8±3,71a | **2,3±0,22b** | 11,8±0,60a | 55,0±2,04a | 0±0a |
|  |  | **RootSec*** | | | | | | |
|  |  | **Quinoline**  **(µg·dm^-3^)** | **5-chloro-7-iodoquin-8-ol**  **(µg·dm^-3^)** | **5,7-diiodo-8-quinolinol**  **(µg·dm^-3^)** | **Hydroxychloroquine**  **(µg·dm^-3^)** | **6-iodo-4-hydroxy-3-quinoline carbocyclic acid**  **(µg·dm^-3^)** | **7-iodo-4-hydroxy-3-quinoline carbocyclic acid**  **(µg·dm^-3^)** | **8-iodo-4-hydroxy-3-quinoline carbocyclic acid**  **(µg·dm^-3^)** |
| RootSec | Control | 1 264,2±14,9a | 2,9±0,11b | 1,2±0,13b | 17,2±0,55b | **41,6±5,06d** | 11,3±1,05a | 8,236±b |
|  | KIO_3_ | 3 406,2±10,29b | 1,7±0,57a | 0±0a | 18,5±0,93b | 12,4±1,71b | 82,5±3,66c | 10.99±b |
|  | 5-iodosalicylic acid | 4 392,6±36,68c | 8,7±0,69c | 4,1±0,32c | **24,6±1,18c** | 7,1±0,48a | 29,7±3,4b | 4.524±a |
|  | 3,5-diiodosalicylic acid | **6 315,9±135,54d** | **22,2±0,79d** | **12,9±0,42d** | 12,1±0,4a | 26,7±1,61c | **154,8±8,83d** | 2.687±a |

*RootSec - Results of the determination of individual compounds in secretions collected as a result of root pressure– this is in white secretion on the surface of the root neck after cutting the heads (lettuce leaves). <LOQ - Below limit of quantification (LOQ). Means in the column followed by different letters differ significantly at P < 0.05 (n=4; means from two cultivation cycles).

**Material and methods for this table - description and reference literature:**

All compounds in this table were determined using the same analytical method as for the iodine metabolites listed in Tables S4 and S5 − method with using LC-MS/MS technique according to Smoleń et al. (2021).

*References:*

Smoleń, S., et al. 2021 New Aspects of Uptake and Metabolism of Non-organic and Organic Iodine Compounds — The Role of Vanadium and Plant-Derived Thyroid Hormone Analogs in Lettuce. Frontiers in Plant Science, 12, 608. doi: 10.3389/fpls.2021.653168.

**Table S4.** Concentrations of chlorogenic, sinapic, p-coumaric, ferulic and 3-hydroxybenzoic acids in lettuce leaves.

|  |  | **Chlorogenic acid**  **(mg·100g^-1^ F.W.)** | **Sinapic acid (mg·100g^-1^ F.W.)** | **p-coumaric acid (mg·100g^-1^ F.W.)** | **Ferulic acid**  **(mg·100g^-1^ F.W.)** | **3-hydroxybenzoic**  **(mg·100g^-1^ F.W.)** |
| --- | --- | --- | --- | --- | --- | --- |
| **Part of plant** | **Treatment** | 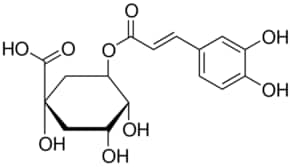 | 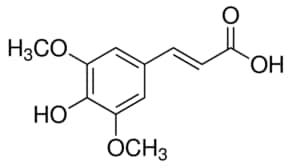 | 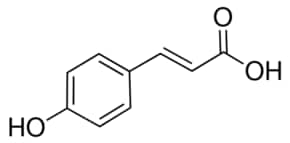 | 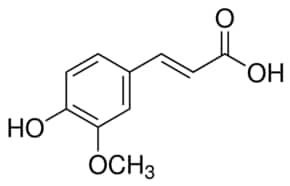 | 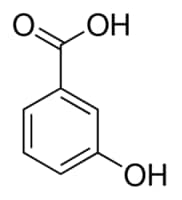 |
|  |  |  |  |  |  |  |
|  |  | **Leaves** | | | | |
| Leaves | Control | 183.6ab±40.1 | 1.80a±0.149 | 2.72b±0.129 | 14.9b±5.53 | **14.37c±4.54** |
|  | SA | 154.1a±28.9 | 1.58a±0.098 | 2.42ab±0.099 | 10.8a±4.01 | 11.74ab±3.44 |
|  | KIO_3_ | 163.3a±25.9 | 1.59a±0.119 | 2.26a±0.089 | 11.3a±4.14 | 14.23c±4.23 |
|  | KIO_3_+SA | **199.7b±35.7** | 2.10b±0.090 | 2.59b±0.096 | 16.9b±6.19 | 10.73a±2.78 |
|  | 5-iodosalicylic acid | **204.6b±33.4** | 2.16b±0.167 | **3.63c±0.231** | 17.1b±6.50 | 11.82ab±2.49 |
|  | 3,5-diiodosalicylic acid | **406.4c±60.0** | **3.41c±0.257** | **5.78d±0.648** | **53.3c±10.24** | 13.29bc±2.96 |

Means in the column followed by different letters differ significantly at P < 0.05 (n=8; means from two cultivation cycles).

**Material and methods for this table - description and reference literature:**

Samples for polyphenol content, chlorogenic, sinapic, *p*-coumaric, ferulic and 3-hydroxybenzoic acid determination with the HPLC method were prepared according to the procedure described in Klimczak et al. (2007) in modification Skoczylas et. al. (2020). NaOH (2 M) was added (1:1, v/v) to the lettuce extracts prepared as described above, and the sample was mixed using a Labnet vortex mixer (Edison, USA) and left in a dark place for 4 h (room temperature). Then, it was neutralised to a pH from 2.1 to 2.6 with HCl (2 mol·dm^-3^) using a pH meter (Metrohm, Herisau, Switzerland) and transferred quantitatively to a measuring flask with 1% L-ascorbic acid dissolved in methanol. Before the chromatographic analysis, the samples were centrifuged in an MPW-260R centrifuge (Warsaw, Poland) (18,000 RPM, 20 min, 4°C) and filtered through a PTFE-L filter with a pore diameter of 0.22 µm. Before injection onto the column, the samples were stored at 4°C.

The chromatographic analysis was carried out in the HPLC Dionex UltiMate 3000 system with DAD detector (Thermo Scientific, Germering, Germany), using a Cosmosil 5C_18_-MS-II column (250 × 4.6 mm ID, 5 µm) (Nacalai Tesque, INC., Kyoto, Japan). Two eluents were used as the mobile phase: A–2% (v/v) an aqueous solution of acetic acid and B–100% methanol. The flow rate of the mobile phase was 1 mL·min^-1^ throughout the analysis, which lasted 50 min and was performed in the following system of eluents: eluent A–0 min 95%, 10 min 70%, 25 min 50%, 35 min 30% and 40 min 95%.

*References:*

Klimczak, I., Małecka, M., Szlachta, M., Gliszczyńska‐Świgło, A. (2007). Effect of storage on the content of polyphenols, vitamin C and the antioxidant activity of orange juices. Journal of Food Composition and Analysis, 20, 313–322, <https://doi.org/10.1016/j.jfca.2006.02.012>.

Skoczylas, Ł., Tabaszewska, M., Smolen, S., Słupski, J., Liszka-Skoczylas, M., & Baranski, R. (2020). Carrots (*Daucus carota* L.) biofortified with iodine and selenium as a raw material for the production of juice with additional nutritional functions. Agronomy, 10(9), 1–17. https://doi.org/10.3390/agronomy10091360

**Table S5.** Concentrations of water-soluble vitamins: B3 /nicotinic acid/, B7 /D-biotin/, PP /nicotinamide/, B5 /pantothenic acid/, B6 /pyridoxine/B2 /riboflavin/, B9 /folic acid/, B1 /thiamine/ and L-ascorbic acid /vitamin C/ in lettuce leaves.

|  |  | **Concentrations in leaves** | | | | |
| --- | --- | --- | --- | --- | --- | --- |
| **Part of plant** | **Treatment** | **B3 /nicotinic acid/**  **(mg·kg^-1^ D.W.)** | **B7 /D-biotin/**  **(mg·kg^-1^ D.W.)** | **PP /nicotinamide/**  **(mg·kg^-1^ D.W.)** | | **B5 /pantothenic acid/**  **(mg·kg^-1^ D.W.)** |
|  |  | 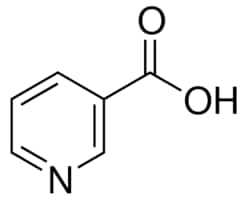 | 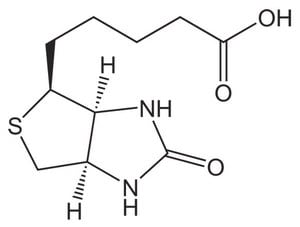 | 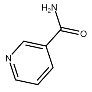 | | 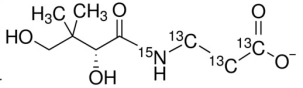 |
| Leaves | Control | 3.36ab±0.620 | 0.105ab±0.0112 | 7.32a±1.768 | | 5.24ab±0.206 |
|  | SA | 2.70a±0.606 | 0.086ab±0.0064 | 7.02a±2.219 | | 4.89a±0.124 |
|  | KIO_3_ | 4.89bc±0.359 | 0.084ab±0.0063 | **13.95c±0.485** | | **5.97c±0.245** |
|  | KIO_3_+SA | 2.72a±0.239 | **0.111b±0.0099** | 8.18a±2.455 | | 4.96a±0.249 |
|  | 5-iodosalicylic acid | 3.19a±0.649 | 0.096ab±0.0101 | 8.51b±2.246 | | 4.86a±0.047 |
|  | 3,5-diiodosalicylic acid | **5.22c±0.718** | 0.076a±0.0085 | 7.85a±1.687 | | 5.30bc±0.079 |
|  |  | **B6 /pyridoxine/**  **(mg·kg^-1^ D.W.)** | **B2 /riboflavin/**  **(mg·kg^-1^ D.W.)** | **B9 /folic acid/**  **(mg·kg^-1^ D.W.)** | **B1 /thiamine/**  **(mg·kg^-1^ D.W.)** | **L-ascorbic acid /vitamin C/**  **(mg 100·g^-1^ F.W.)** |
|  |  | 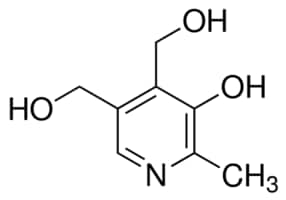 | 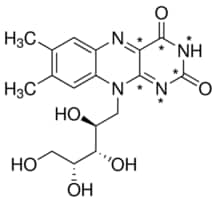 | 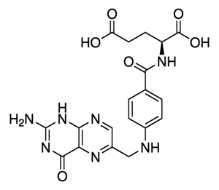 | 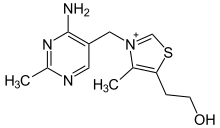 | 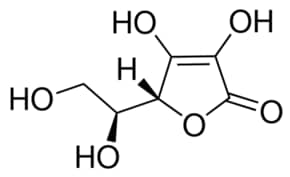 |
| Leaves | Control | 0.324b±0.0231 | 5.41a±0.794 | **0.981b±0.2064** | 4.51ab±0.1069 | 20.45ab±1.620 |
|  | SA | 0.343bc±0.0227 | 4.69a±0.255 | 0.498a±0.0978 | 5.07bc±0.1818 | 19.07ab±1.393 |
|  | KIO_3_ | **0.376c±0.0054** | 5.34a±0.651 | 0.758ab±0.1676 | **5.30c±0.1723** | 16.89a±2.598 |
|  | KIO_3_+SA | 0.285ab±0.0598 | 5.05a±0.711 | 0.457a±0.0739 | 3.43a±0.2916 | 18.52ab±3.194 |
|  | 5-iodosalicylic acid | 0.204a±0.0347 | 5.72a±0.613 | 0.486a±0.0437 | 4.42a±0.4332 | 28.50bc±1.612 |
|  | 3,5-diiodosalicylic acid | 0.138a±0.0308 | 4.16a±0.422 | 0.457a±0.0552 | 4.47a±0.3294 | **35.58c±6.727** |

Means in the column followed by different letters differ significantly at P < 0.05 (n=8; means from two cultivation cycles).

**Material and methods for this table - description and reference literature:**

The contents of the following wateL-soluble vitamins were analysed using the LC-MS/MS technique according to the procedure described in Smoleń et al. (2022): B1,B2, B3, B5, B6, B7, B9 and PP. The sample preparation method was as follows: 0.075 g aiL-dried, ground plant samples were weighted in 7 mL polypropylene tubes, and 5 mL of extractant was added. The extractant was a mixture of ACN/H_2_O/100 mM NH_4_Ac (90/5/5, v/v/v), pH 5.8. After mixing, samples were incubated for 15 min at room temperature in an ultrasonic bath, mixed thoroughly and centrifuged for 15 min at 4500 rpm. The supernatants were filtered through a 0.22-μm syringe filter (Santos et al. 2012). The contents of these wateL-soluble vitamins were determined in these extracts using HPLC-ESI-MS/MS. A mass spectrometer with electrospray ionisation (4500 Qtrap, Sciex) was coupled to a liquid chromatograph (Ultimate 3000, Thermo Scientific). See more details in Smoleń et al. (2022).

*References:*

Santos, J., Mendiola, J. A., Oliveira, M. B. P. P., Ibanez, E., Herrero, M. (2012) Sequential determination of fat- and wateL-soluble vitamins in green leafy vegetables during storage, Journal of Chromatography A, 1261 179–188. https://doi.org/[10.1016/j.chroma.2012.04.067](https://doi.org/10.1016/j.chroma.2012.04.067)

Smoleń, S., Kowalska, I., Skoczylas, Ł., Tabaszewska, M., Pitala, J., Mrożek, J., & Kováčik, P. (2022). Effectiveness of enriching lettuce with iodine using 5-iodosalicylic and 3, 5-diiodosalicylic acids and the chemical composition of plants depending on the type of soil in a pot experiment. Food Chemistry, 382, 132347. [doi.org/10.1016/j.foodchem.2022.132347](https://doi.org/10.1016/j.foodchem.2022.132347)

**Table S6.** Concentrations of sucrose (S), glucose (G), fructose (F) and sum of sugars (S+G+F), and free amino acids; activity of peroxidase (POX), catalase /CAT/, guaiacol peroxidase /POX/ as well as anti-radical activity DPPH, ABTS and FRAP in lettuce leaves.

| **Part of plant** | **Treatment** | **Sucrose**  **(mg·100g^-1^ F.W.)** | | **Glucose**  **(mg·100g^-1^ F.W.)** | | **Fructose**  **(mg·100g^-1^ F.W.)** | | | **Sum of sugars (S+G+F)**  **(mg·100g^-1^ F.W.)** | | **Free amino acids [mg N·100g^-1^ F.W.]** | |
| --- | --- | --- | --- | --- | --- | --- | --- | --- | --- | --- | --- | --- |
|  |  | **Concentrations in leaves** | | | | | | | | | | |
| Leaves | Control | **51.86b±6.764** | | 97.84a±20.342 | | 104.06ab±20.515 | | | 253.8ab±47.00 | | **11.59d±0.687** | |
|  | SA | 37.53ab±3.032 | | 64.96a±13.521 | | 79.26a±18.129 | | | 181.7a±34.45 | | 11.23cd±0.543 | |
|  | KIO_3_ | 40.54ab±2.848 | | 81.19a±14.487 | | 95.08ab±15.185 | | | 216.8ab±32.13 | | 10.28bcd±0.465 | |
|  | KIO_3_+SA | 21.32a±4.606 | | 84.52a±20.306 | | 93.19ab±20.926 | | | 199.0ab±36.65 | | 8.55ab±1.089 | |
|  | 5-iodosalicylic acid | 38.61ab±7.590 | | 110.88ab±11.936 | | 138.68b±13.477 | | | 288.2b±19.10 | | 7.34a±0.302 | |
|  | 3,5-diiodosalicylic acid | **59.06b±14.596** | | **154.71b±9.905** | | **200.52c±12.800** | | | **414.3c±11.88** | | 9.32abc±0.868 | |
|  |  | **Activity in leaves** | | | | | | | | | | |
|  |  | **Polyphenol oxidases (PPO)**  **(ΔE_420_·min·g^-1^ F.W.)** | **Peroxidase (POX)**  **(ΔE485·min·g^-1^ F.W)** | | **Catalase /CAT/ activity**  **(U·mg^-1^ protein)** | | **Guaiacol peroxidase /G-POX/ activity**  **(U·mg^-1^ protein)** | **DPPH**  **(μmol Tx/g F.W.)** | | **ABTS**  **(μmol troloxu/g F.W.)** | | **FRAP**  **(μmol Fe^2+^/g F.W.)** |
| Leaves | Control | 6.43ab±0.088 | 13.56ab±0.107 | | 0.0435a±0.01048 | | **0.0126b±0.00417** | 2.22a±0.064 | | 18.83a±1.826 | | 1.56a±0.074 |
|  | SA | **7.89c±0.128** | **16.89c±0.967** | | 0.1095ab±0.00769 | | 0.0055a±0.00130 | 3.46a±0.419 | | 19.57a±2.279 | | 1.78a±0.093 |
|  | KIO_3_ | 7.51bc±0.360 | 13.62ab±0.352 | | 0.0838a±0.00845 | | 0.0033a±0.00074 | 2.29a±0.258 | | 17.19a±1.486 | | 1.50a±0.041 |
|  | KIO_3_+SA | 6.30a±0.672 | 13.77ab±0.314 | | 0.1058ab±0.01078 | | 0.0035a±0.00072 | 3.75a±0.167 | | 20.02a±0.941 | | 1.91a±0.063 |
|  | 5-iodosalicylic acid | 5.27a±0.088 | 13.09a±0.207 | | **0.1253b±0.01838** | | 0.0034a±0.00070 | 3.30a±0.162 | | 20.44a±0.4  36 | | 2.14a±0.093 |
|  | 3,5-diiodosalicylic acid | 5.96a±0.532 | 14.68b±0.247 | | 0.0679a±0.01366 | | 0.0029a±0.00032 | **7.36b±2.067** | | **32.83b±4.824** | | **3.40b±0.768** |

Means in the column followed by different letters differ significantly at P < 0.05 (n=8; means from two cultivation cycles).

**Material and methods for this table - description and reference literature:**

Total sugars, as a sum of glucose, fructose, and sucrose, were measured in the ethanol extracts, using the reverse capillary electrophoresis technique with the PA 800 Plus system (Beckman) according to the procedure described in Rakoczy-Lelek et al. (2021). The level of free amino acids was determined spectrophotometrically after a reaction with ninhydrin (Smoleń et al. 2016). In order to determine the PPO activity, an aliquot of the extract was mixed with a catechol solution (0.07 mol dm^-3^ in a 0.05 mol dm^-3^ phosphate buffer solution) and the increase in absorbance was measured at 420 nm. The POX determination was carried out by adding phosphate buffer to the lettuce extract and simultaneously a solution of H_2_O_2_ (3 mmol dm^-3^) and p-phenyldiamine (1g/100g) and then measuring the increase in absorbance at 485 nm. Measurements of the changes in absorbance were carried out in 3 minutes in both cases. The enzyme activity was determined from the rectilinear section of the curve. To determine the activity of polyphenyl oxidase (PPO) and peroxidase (POX), a sample of lettuce was ground with 0.2 mol dm^-3^ phosphate buffer at pH 7. The appropriate sample was transferred to a volumetric flask and filled with 0.05 mol dm^-3^ phosphate buffer and left for 2 hours in the refrigerator (Beers et al. 1952, Reuveni et al. 1992). Activity of CAT and G-POX were measured according to the procedure described in Halka et al. (2020). Antioxidative activity against DPPH, ABTS and FRAP radicals were measured in ethanolic extracts prepared by weighing portions of lettuce and mixed them with 80% ethanol, followed by refluxing for 15 min. After cooling, the samples were filtered. Prepared extract was used for analyses with the spectrophotometric method using a Hitachi U-2900 UV-VIS spectrophotometer (Hitachi, Tokyo, Japan). Free DPPH radical (1,1-diphenyl-2-picrylhydrazyl) and ABTS radical (2,2'-azino-bis (3-ethylbenzothiazoline-6-sulfonic acid) was used for analisis the anti-free-radical activity of lettuce. Absorbance was measured 10 min after the free radical solution had been added to the sample, at wavelengths of 516 nm for DPPH and 734 nm for ABTS (Brand-Williams et al 1995, Re et al. 1999). The antioxidative activity of lettuce was expressed in w μM Trolox per g of lettuce. The analysis of the antioxidant activity by the FRAP method was performed according to Benzie et al. (1996). TPTZ solution was added to the lettuce ethanol extract and incubated at 37°C for 10 minutes. After this time, the samples were cooled and the absorbance was measured at 516 nm. The results are expressed as μmol Fe^2+^/g of lettuce.

*References:*

Beers, R.F., Sizer, I.W.A. (1952). Spectrophotometric method for measuring the breakdown of hydrogen peroxide by catalase. J. Biol. Chem. 195, 133–140, doi:10.1093/jxb/48.2.181.

Benzie, I.F.F.; Strain, J.J. (1996). The Ferric Reducing Ability of Plasma (FRAP) as a Measure of ‘“Antioxidant Power”’: The FRAP Assay. Anal. Biochem. 2, 70–76.

Brand-Williams, W.; Cuvelier, M.E.; Berset, C. (1995). Use of a Free Radical Method to Evaluate Antioxidant Activity. Leb. Wiss. Technol. 28, 25–30.

Halka, M., Smoleń, S., Ledwożyw-Smoleń, I., & Sady, W. (2020). Comparison of effects of potassium iodide and iodosalicylates on the antioxidant potential and iodine accumulation in young tomato plants. Journal of Plant Growth Regulation, 39(1), 282-295. doi.org/10.1007/s00344-019-09981-2

Rakoczy-Lelek, R., Smoleń, S., Grzanka, M., Ambroziak, K., Pitala, J., Skoczylas, Ł., ... & Kardasz, H. (2021). Effectiveness of foliar biofortification of carrot with iodine and selenium in a field condition. Frontiers in plant science, 12, 656283. doi: 10.3389/fpls.2021.656283

Re, R.; Pellegrini, N.; Proteggente, A.; Pannala, A.; Yang, M.; Rice-Evans, C. (1999). Development and characterisation of carbon nanotube-reinforced polyurethane foams. Free Radic. Biol. Med. 26, 231–1237.

Reuveni, R.; Shimoni, M.; Karchi, Z.; Kuc, J. (1992). Peroxidase Activity as a Biochemical Marker for Resistance of Muskmelon (Cucumis melo) to Pseudoperonospora cubensis. Resistance 82, 749–753.

Smoleń, S., Skoczylas, Ł., Ledwożyw-Smoleń, I., Rakoczy, R., Liszka-Skoczylas, M., Kopeć, A., ... & Sady, W. (2016). The quality of carrot (Daucus carota L.) cultivated in the field depending on iodine and selenium fertilization. Folia Horticulturae, 28(2), 151. doi: 10.1515/fhort-2016-0018.

**Table S7.** Concentrations of ash, crude fat, protein, dietary fiber and digestible carbohydrates in lettuce leaves.

| **Part of plant** | **Treatment** | **Ash**  **(g·100g^-1^ D.W.)** | **Crude fat**  **(g·100g^-1^ D.W.)** | **Protein**  **(g·100g^-1^ D.W.)** | **Dietary fiber**  **(g·100g^-1^ D.W.)** | **Digestible carbohydrates**  **(g·100g^-1^ D.W.)** |
| --- | --- | --- | --- | --- | --- | --- |
|  |  | **Concentrations in leaves** | | | | |
| Leaves | Control | 21.54±0.63bc | **3.27±0.56c** | **25.76±0.97b** | 26.73±1.42ab | 22.71±3.34a |
|  | SA | 19.36±3.58ab | 2.14±0.36ab | 21.93±1.71a | 27.5±1.35b | **29.07±3.87c** |
|  | KIO_3_ | **22.00±0.14c** | 1.67±0.36a | **26.43±0.85b** | 26.22±2.01ab | 23.68±1.49a |
|  | KIO_3_+SA | 20.15±1.18ab | 2.05±0.47ab | 24.03±2.28ab | 26.11±1.12a | 27.65±3.03c |
|  | 5-iodosalicylic acid | 19.19±2.8ab | 2.95±0.20bc | 24.01±0.83ab | **28.75±3.58c** | 25.10±5.71ab |
|  | 3,5-diiodosalicylic acid | 18.36±2.8a | 2.49±0.33bc | 24.22±0.49ab | **28.22±0.8bc** | 25.95±2.54b |

Means in the column followed by different letters differ significantly at P < 0.05 (n=8; means from two cultivation cycles).

**Material and methods for this table - description and reference literature:**

Determination of ash content: The mineral content form of ash determined the method (PN-A-79011-8: 1998). Samples were weighed, ashed over a burner, and finally in a muffle furnace. The time and rate of the process adjusted accordingly so that the organic compounds ashed and only the minerals forming the crude ash remained (AOAC procedure No. 930.05).

The total protein content carried the Kjeldahl method (PN-EN ISO 8968-1: 2014-03). The sample was previously lyophilized and mineralized in concentrated sulphuric acid (VI). Then the ammonia formed from the nitrogenous bonds contained in the protein was distilled off. Titrated with 0.1M hydrochloric acid in the presence of a Tashiro indicator to light purple color (AOAC procedure No. 950.36).

The fat content performed the Soxhlet method (PN-A-79011-4: 1998). The sample subjected to continuous multiple extractions with a Foss Soxtec 2050 Solvent Extraction System apparatus. The organic solvent in the form of petroleum ether was driven off and the mass of the extracted fat was determined (AOAC procedure No. 950.38).

Determination of total dietary fiber was based on AACC method 32-05.01 and AOAC Method 985.29, using a commercially available test kit company Megazyme. A sample of the lettuce has been submitted to enzyme digestion (including thermostable a-amylase, purified protease, and purified amyloglucosidase).

Total carbohydrates are expressed using the formula: 100 - the sum of total fat (g), total protein (g), minerals in the form of ash (g), and dietary fiber (g).

*References:*

Association of Official Analytical Chemists (1986). Changes in methods. J. Assoc. Off. Anal. Chem., 69, 370.

Association of Official Analytical Chemists (1987). Changes in methods. J. Assoc. Off. Anal. Chem., 70, 393.

Association of Official Analytical Chemists (2005). Official Methods of Analysis 18th ed.,Washington, DC, USA.

Association of Official Analytical Chemists. (1985). Official Methods of Analysis, 14th ed., 1st suppl. Secs. 43, A14-43, A20, p.399.

Hennigs C. (2020) Natural Enzymes for Ecological Detergents to improve biologically certified Washing products. 1848056 – 31/03/2020. Access 04.08.2022 https://ec.europa.eu/research/participants/documents/downloadPublic?documentIds=080166e5cd8d669b&appId=PPGMS

Prosky, L., Asp, N. G., Furda, I., DeVries, J. W., Schweizer, T. F. & Harland, B. F. (1985). Determination of total dietary fibre in foods and food products: Collaborative study. J. Assoc. Off. Anal. Chem., 68, 677.

Prosky, L., Asp, N. G., Schweizer, T. F., DeVries, J. W. & Furda, I. (1988). Determination of insoluble, soluble, and total dietary fibre in foods and food products. J. Assoc. Off. Anal. Chem., 71, 1017.

**Table S8.** Concentrations of ammonium ion, nitrates(V) and nitrates(III) in leaves and roots of lettuce.

| **Part of plant** | **Treatment** | **NH4^+^**  **[mg∙kg^-1^ F.W.]** | **NO_3_^-^**  **[mg∙kg^-1^ F.W.]** | **NO_2_^-^**  **[mg∙kg^-1^ F.W.]** |
| --- | --- | --- | --- | --- |
| **Concentrations in leaves** | | | | |
| Leaves | Control | 20.51bc±2.050 | 4 639.6b±158.27 | 0.793ab±0.3423 |
|  | SA | 18.67ab±2.566 | 5 061.5bc±76.94 | 0.512a±0.1863 |
|  | KIO_3_ | **28.17c±4.808** | **5 258.9c±210.63** | 0.554a±0.2112 |
|  | KIO_3_+SA | 19.21ab±2.929 | **5 295.1c±107.57** | 0.546a±0.2086 |
|  | 5-iodosalicylic acid | 13.16ab±1.209 | 4 690.7b±170.83 | 1.322b±0.1859 |
|  | 3,5-diiodosalicylic acid | 11.70a±0.435 | 2 401.5a±311.40 | 0.648ab±0.1671 |
| **Concentrations in roots** | | | | |
| Roots | Control | 5.84a±1.078 | **1 972.9b ±134.80** | 12.08a±4.676 |
|  | SA | 8.60a±1.553 | 1 744.1 ab ±105.82 | 7.06a±2.220 |
|  | KIO_3_ | 8.75a±2.322 | 1 775.7ab±39.58 | 8.69a±2.539 |
|  | KIO_3_+SA | 8.73a±1.787 | **2 005.8b±129.56** | **25.11b±6.490** |
|  | 5-iodosalicylic acid | 11.55a±2.682 | 1 876.0ab±164.65 | 14.78ab±3.840 |
|  | 3,5-diiodosalicylic acid | 13.81a±4.313 | 1 430.3a±69.76 | 10.65a±3.018 |

Means in the column followed by different letters differ significantly at P < 0.05 (n=8; means from two cultivation cycles).

**Material and methods for this table - description and reference literature:**

To analyse the level of ammonium ion, nitrates(V) and nitrates(III) were measure in lettuce samples, extraction with 2% acetic acid was conducted. All the ions were determined by an AQ2 Discrete Analyser (Seal Analytical, USA) using the methodical protocols provided by the manufacturer of this analyser (Smoleń et al. 2019).

*References:*

Smoleń, S., Kowalska, I., Kováčik, P., Sady, W., Grzanka, M., & Kutman, U. B. (2019). Changes in the chemical composition of six lettuce cultivars (Lactuca sativa L.) in response to biofortification with iodine and selenium combined with salicylic acid application. Agronomy, 9(10), 660. https://doi.org/10.3390/agronomy9100660

**Table S9.** Concentrations of N, P, K, Mg, Ca, S and Na in leaves and roots of lettuce.

| **Part of plant** | **Treatment** | **N% D.W.** | **P% D.W.** | **K% D.W.** | **Mg% D.W.** | **Ca% D.W.** | **S% D.W.** | **Na% D.W.** |
| --- | --- | --- | --- | --- | --- | --- | --- | --- |
|  |  | **Concentrations in leaves** | | | | | | |
| Leaves | Control | 4.37ab±0.199 | 0.707a±0.0511 | 8.12ab±0.242 | 0.394a±0.0222 | 1.32a±0.027 | 0.219a±0.0283 | 0.010a±0.0040 |
|  | SA | 3.85a±0.108 | 0.641a±0.0718 | 6.82a±0.896 | 0.353a±0.0433 | 1.25a±0.052 | 0.201a±0.0304 | 0.156b±0.0157 |
|  | KIO_3_ | **4.59b±0.099** | 0.688a±0.0567 | 8.17ab±0.467 | 0.371a±0.0293 | 1.30a±0.057 | 0.204a±0.0248 | 0.106a±0.0060 |
|  | KIO_3_+SA | 4.12ab±0.086 | 0.703a±0.0259 | **8.90b±0.400** | **0.509b±0.0122** | **1.90c±0.045** | 0.244a±0.0073 | 0.147b±0.0025 |
|  | 5-iodosalicylic acid | 4.37ab±0.339 | 0.668a±0.0360 | 6.47a±0.772 | 0.476b±0.0137 | 1.76b±0.032 | 0.254a±0.0082 | **0.218c±0.0271** |
|  | 3,5-diiodosalicylic acid | 3.96a±0.096 | 0.611a±0.0100 | 7.75ab±0.665 | 0.373a±0.0025 | 1.21a±0.057 | 0.247a±0.0076 | 0.081a±0.0077 |
|  |  | **Concentrations in roots** | | | | | | |
| Roots | Control | 4.39ab±0.230 | 2.24a±0.387 | **6.79b±0.082** | 0.196ab±0.0215 | **2.13b±0.545** | **0.854b±0.0460** | 0.085a±0.0073 |
|  | SA | **4.64b±0.099** | 1.61a±0.141 | **6.81b±0.178** | 0.172a±0.0087 | 1.35ab±0.197 | **0.915b±0.0741** | 0.081a±0.0036 |
|  | KIO_3_ | **4.65b±0.175** | 1.77a±0.202 | 6.48ab±0.310 | 0.170a±0.0096 | 1.63ab±0.311 | **0.852b±0.0592** | 0.077a±0.0011 |
|  | KIO_3_+SA | **4.54b±0.182** | 2.03a±0.297 | 6.32ab±0.376 | 0.180ab±0.0113 | 1.95ab±0.426 | **0.803b±0.0626** | 0.084a±0.0016 |
|  | 5-iodosalicylic acid | **4.57b±0.121** | 1.58a±0.136 | 6.28ab±0.404 | 0.182ab±0.0037 | 1.21ab±0.158 | 0.533a±0.0103 | 0.115b±0.0056 |
|  | 3,5-diiodosalicylic acid | 4.03a±0.087 | 1.67a±0.106 | 5.88a±0.201 | **0.214b±0.0054** | 0.92a±0.110 | 0.419a±0.0561 | **0.136c±0.0084** |

Means in the column followed by different letters differ significantly at P < 0.05 (n=8; means from two cultivation cycles).

**Material and methods for this table - description and reference literature:**

N-total was determined by the Kjeldahl method using oven mineralisation, a Foss Digestor 2020 by TecatorTM and a Velp UDK 139 Semi- Automatic Distillation Unit. Contents of P, K, Mg, Ca, S and Na were determined by the ICP-OES technique according to the method described by Kalisz et al. (2019). Next, 0.5 g samples were placed into 55-mL TFM vessels and were mineralised in 10 mL 65% super pure HNO3 (Merck no. 100443.2500) in a Mars 5 Xpress (CEM, USA) microwave digestion system. The following mineralisation procedure was applied: 15 min. (the time needed to achieve a temperature of 200 ℃) and 20 min maintaining the temperature. After cooling, the samples were quantitatively transferred to 25-mL graduated flasks with redistilled water. Contents of mentioned elements were determined using a high-dispersion inductively coupled plasma optical emission (Smoleń et al. 2022).

*References:*

Kalisz, A., Sękara, A., Smole´n, S., Grabowska, A., Gil, J., Komorowska, M., et al. (2019). Survey of 17 elements, including rare earth elements, in chilled and non-chilled cauliflower cultivars. Scientific Reports, 9(1). https://doi.org/10.1038/s41598-019- 41946-z

Smoleń, S., Kowalska, I., Skoczylas, Ł., Tabaszewska, M., Pitala, J., Mrożek, J., & Kováčik, P. (2022). Effectiveness of enriching lettuce with iodine using 5-iodosalicylic and 3, 5-diiodosalicylic acids and the chemical composition of plants depending on the type of soil in a pot experiment. Food Chemistry, 382, 132347. [doi.org/10.1016/j.foodchem.2022.132347](https://doi.org/10.1016/j.foodchem.2022.132347)

**Table S10.** Concentrations of B, Cu, Fe, Mn, Mo and Zn in leaves and roots of lettuce.

| **Part of plant** | **Treatment** | **B**  **(mg·kg^-1^ D.W.)** | **Cu**  **(mg·kg^-1^ D.W.)** | **Fe**  **(mg·kg^-1^ D.W.)** | **Mn**  **(mg·kg^-1^ D.W.).** | **Mo**  **(mg·kg^-1^ D.W.)** | **Zn**  **(mg·kg^-1^ D.W.)** |
| --- | --- | --- | --- | --- | --- | --- | --- |
|  |  | **Concentrations in leaves** | | | | | |
| Leaves | Control | 27.19a±1.889 | 7.17b±0.582 | 89.33a±2.393 | 128.6a±20.06 | 0.863c±0.0414 | **46.85b±3.823** |
|  | SA | 26.90a±1.787 | 5.57ab±1.106 | 89.23a±3.695 | 130.2a±6.40 | 0.170a±0.0584 | 44.32ab±3.558 |
|  | KIO_3_ | 25.91a±2.171 | 6.53b±0.304 | 76.13a±4.922 | 140.5a±15.57 | 0.648b±0.0356 | 44.74ab±1.767 |
|  | KIO_3_+SA | 34.61b±0.525 | 4.85ab±0.704 | 1 15.04b±1.417 | **223.2b±20.26** | 1.433d±0.1014 | 38.82ab±0.919 |
|  | 5-iodosalicylic acid | 39.97c±0.576 | 4.54ab±0.846 | **1 28.93bc±5.803** | 164.0a±3.02 | 0.572b±0.0556 | 44.16ab±1.071 |
|  | 3,5-diiodosalicylic acid | **42.59d±0.938** | 3.62a±1.221 | **1 45.65c±12.779** | 158.0a±31.24 | **1.637e±0.0329** | 35.93a±4.535 |
|  |  | **Concentrations in roots** | | | | | |
| Roots | Control | 8.13a±0.190 | 33.81bc±2.977 | **2 886.3c±183.58** | 742.9a±131.44 | 23.54a±3.141 | **123.15d±6.318** |
|  | SA | 10.34b±0.419 | 37.14c±4.187 | **2 852.3c±28.27** | 582.3a±90.49 | 28.65a±4.272 | 112.13c±6.061 |
|  | KIO_3_ | 9.60b±0.384 | 29.29abc±2.468 | **2 909.0c±26.25** | 666.8a±100.23 | 26.06a±4.055 | 108.60bc±2.544 |
|  | KIO_3_+SA | 9.40b±0.386 | 26.30ab±1.838 | **3 027.9c±183.92** | 719.9a±170.11 | 40.60a±9.922 | 99.16b±1.080 |
|  | 5-iodosalicylic acid | 14.77c±0.271 | 24.29a±3.148 | 1 330.5b±43.70 | 731.9a±95.12 | 32.14a±7.653 | 67.74ab±4.762 |
|  | 3,5-diiodosalicylic acid | **19.79d±0.733** | 16.05a±1.291 | 879.8a±45.26 | 786.7a±73.70 | 36.40a±10.192 | 52.72a±2.980 |

Means in the column followed by different letters differ significantly at P < 0.05 (n=8; means from two cultivation cycles).

**Material and methods for this table - description and reference literature:**

Contents of B, Cu, Fe, Mn, Mo and Zn were determined by the ICP-OES technique according to the method described by Kalisz et al. (2019). Next, 0.5 g samples were placed into 55-mL TFM vessels and were mineralised in 10 mL 65% super pure HNO3 (Merck no. 100443.2500) in a Mars 5 Xpress (CEM, USA) microwave digestion system. The following mineralisation procedure was applied: 15 min. (the time needed to achieve a temperature of 200 ◦C) and 20 min maintaining the temperature. After cooling, the samples were quantitatively transferred to 25-mL graduated flasks with redistilled water. Contents of mentioned elements were determined using a high-dispersion inductively coupled plasma optical emission (Smoleń et al. 2022).

*References:*

Kalisz, A., Sękara, A., Smole´n, S., Grabowska, A., Gil, J., Komorowska, M., et al. (2019). Survey of 17 elements, including rare earth elements, in chilled and non-chilled cauliflower cultivars. Scientific Reports, 9(1). https://doi.org/10.1038/s41598-019- 41946-z

Smoleń, S., Kowalska, I., Skoczylas, Ł., Tabaszewska, M., Pitala, J., Mrożek, J., & Kováčik, P. (2022). Effectiveness of enriching lettuce with iodine using 5-iodosalicylic and 3, 5-diiodosalicylic acids and the chemical composition of plants depending on the type of soil in a pot experiment. Food Chemistry, 382, 132347. doi.org/10.1016/j.foodchem.2022.132347

**Table S11.** Genes data and designed primers based on RNA-seq data used in the qRT-PCR analyses.

| **Gene name** | **Lactuca transcript from transcriptome** | **log_2_ Fold Change (based on RNA-seq results)** | | | | **Primers sequence [5’ – 3’]** | **Product length [bp]** |
| --- | --- | --- | --- | --- | --- | --- | --- |
|  |  | **R-KIO3 vs R-Ctrl** | **L-KIO3 vs L-Ctrl** | **R-SA vs R-Ctrl** | **L-SA vs L-Ctrl** |  |  |
| Probable NAD(P)H dehydrogenase (quinone) FQR1-like | XLOC_012062 | 2.1 | - | 1.8 | - | F:ACTCGATGTATGGGCATGTTGA | 131 |
|  |  |  |  |  |  | R:TTTCATCGCCGAGAGAGCGT |  |
| Flavonoid 3',5'-hydroxylase 2 | XLOC_005685 | 1.3 | - | 1.2 | - | F:GAAGATGAAGCAAGCAATACTC | 95 |
|  |  |  |  |  |  | R:AAATAGTCCGACACGTTGTT |  |
| Probable aldo-keto reductase 2 | XLOC_003743 | 2.6 | - | - | - | F:CTTTGAAGGGCGGAATGA | 101 |
|  |  |  |  |  |  | R:TACATAAGCGGGATCTCCA |  |
| F-box/kelch-repeat protein | XLOC_000898 | 1.5 | - | 1.2 | - | F:GTTTGACTCGTTTGCACTATGG | 119 |
|  |  |  |  |  |  | R:CCGTGTGAACCCACTTCTT |  |
| Probable LRR receptoL-like serine/threonine-protein kinase | XLOC_007779 | 1.1 | - | 1.1 | - | F:ACCAGCACAATCGTCGGAAT | 130 |
|  |  |  |  |  |  | R:CGACGTTGCCGTGCTTTTTA |  |
| Adagio 3 | XLOC_000115 | 2.6 | 4.7 | - | - | F:GGAGAGCGGTGGTGGATTTT | 176 |
|  |  |  |  |  |  | R:AACGACAGTTCCGACCAAGG |  |

Transcripts with FDR (False Discovery Rate) < 0.05 were considered as significantly differentially expressed and all selected genes for qRT-PCR analyses were characterized FDR < 0.05 .

| 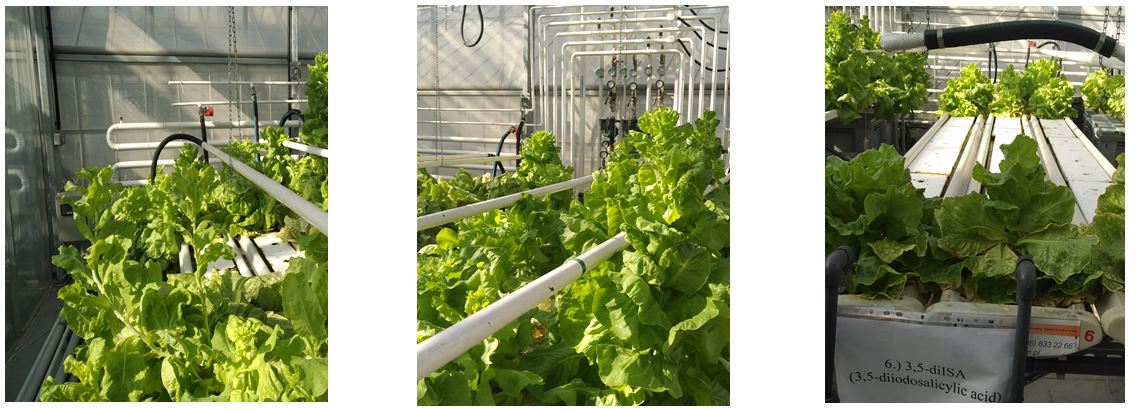 | | |
| --- | --- | --- |
| **Control**  **Initial phase of inflorescence shoot growth** | **5-ISA**  **Advanced growth and development of the inflorescence shoot** | **3,5-diISA**  **Strong inhibition of growth and development of inflorescence shoot** |

**Figure S2.** Lettuce plants left to grow after harvesting the heads. The appearance of plants on 36 days after harvesting heads and roots for biomass assessment and chemical plant analysis.

**A small number of plants after harvest and after sampling for analysis (described throughout the publication) were allowed to visually assess of the growth.**
